# Use of Genomic DNA as an Indirect Reference for Identifying Gender-Associated Transcripts in Morphologically Identical, but Chromosomally Distinct, *Schistosoma mansoni* Cercariae

**DOI:** 10.1371/journal.pntd.0000323

**Published:** 2008-10-22

**Authors:** Jennifer M. Fitzpatrick, Anna V. Protasio, Andrew J. McArdle, Gary A. Williams, David A. Johnston, Karl F. Hoffmann

**Affiliations:** 1 Department of Pathology, University of Cambridge, Cambridge, United Kingdom; 2 Rosalind Franklin Centre for Genomics Research, Hinxton, Cambridge, United Kingdom; 3 Department of Zoology, The Natural History Museum, London, United Kingdom; George Washington University Medical Center, United States of America

## Abstract

**Background:**

The use of DNA microarray technology to study global *Schistosoma* gene expression has led to the rapid identification of novel biological processes, pathways or associations. Implementation of standardized DNA microarray protocols across laboratories would assist maximal interpretation of generated datasets and extend productive application of this technology.

**Methodology/Principal Findings:**

Utilizing a new *Schistosoma mansoni* oligonucleotide DNA microarray composed of 37,632 elements, we show that schistosome genomic DNA (gDNA) hybridizes with less variation compared to complex mixed pools of *S. mansoni* cDNA material (R = 0.993 for gDNA compared to R = 0.956 for cDNA during ‘self versus self’ hybridizations). Furthermore, these effects are species-specific, with *S. japonicum* or *Mus musculus* gDNA failing to bind significantly to *S. mansoni* oligonucleotide DNA microarrays (e.g R = 0.350 when *S. mansoni* gDNA is co-hybridized with *S. japonicum* gDNA). Increased median fluorescent intensities (209.9) were also observed for DNA microarray elements hybridized with *S. mansoni* gDNA compared to complex mixed pools of *S. mansoni* cDNA (112.2). Exploiting these valuable characteristics, *S. mansoni* gDNA was used in two-channel DNA microarray hybridization experiments as a common reference for indirect identification of gender-associated transcripts in cercariae, a schistosome life-stage in which there is no overt sexual dimorphism. This led to the identification of 2,648 gender-associated transcripts. When compared to the 780 gender-associated transcripts identified by hybridization experiments utilizing a two-channel direct method (co-hybridization of male and female cercariae cDNA), indirect methods using gDNA were far superior in identifying greater quantities of differentially expressed transcripts. Interestingly, both methods identified a concordant subset of 188 male-associated and 156 female-associated cercarial transcripts, respectively. Gene ontology classification of these differentially expressed transcripts revealed a greater diversity of categories in male cercariae. Quantitative real-time PCR analysis confirmed the DNA microarray results and supported the reliability of this platform for identifying gender-associated transcripts.

**Conclusions/Significance:**

Schistosome gDNA displays characteristics highly suitable for the comparison of two-channel DNA microarray results obtained from experiments conducted independently across laboratories. The schistosome transcripts identified here demonstrate, for the first time, that gender-associated patterns of expression are already well established in the morphologically identical, but chromosomally distinct, cercariae stage.

## Author Summary

Infection with parasitic schistosome worms causes schistosomiasis, a major neglected tropical disease currently affecting millions of individuals living in the developing world. A virtually unique characteristic of the schistosome life style in comparison to other members of the Phylum Platyhelminthes is that schistosomes are dioecious, having both male and female individuals. While much is known about the adult dioecious state, virtually nothing has been established with regard to the dioecious larval stages such as the snail-infective miracidium and the human-infective cercaria. To shed some light on this under-studied area of schistosome biology, we first developed a new long-oligonucleotide DNA microarray and used this tool to perfect an indirect hybridization strategy utilizing schistosome genomic DNA for large-scale transcription investigations. Second, we used this tool and strategy to characterize the gene expression profiles of infective male and female cercarial life-stages. This led to the identification of several thousand differentially expressed transcripts and demonstrated that gender-associated patterns of transcription (some associated with adult-specific activities) are surprisingly already established in this short-lived larval life-stage. Further functional interrogation of these transcripts will generate a more complete picture of factors and processes underlying the schistosome's dioecious state, which may help in control strategies attempting to prevent sexual maturation, inhibit male–female interactions and limit the production of tissue-damaging eggs.

## Introduction

High-throughput molecular helminthology studies of biomedically important trematodes have steadily increased in number throughout the last decade. This has been particularly evident in the field of schistosomiasis, a deadly parasitic disease currently affecting greater than 200 million people worldwide with an annual death rate of approximately 300,000 individuals [1]. Due to major breakthroughs facilitated by the successful elucidation of transcriptomes [2], the increasingly broad use of DNA microarrays [3], the extended utilization of modern proteomic/glycomic tools [3] and the exploitation of post-transcriptional gene silencing (PTGS) and transgenesis technologies [4],[5],[6], the schistosome research community is now poised to make important contributions in varied biological disciplines including vaccine development and chemotherapy.

DNA microarray analysis of schistosome transcriptomes has proven to be a major contributor in the identification of novel biological processes, pathways and associations. Most schistosome DNA microarray hybridizations are based on the concept of two-channels, where fluorescent signal intensities originating from the co-hybridization of two different cDNA samples labelled with one of two dyes (Cyanine, Cy3/Cy5 or Alexafluor, AF555/AF647) are compared. After a series of iterative filtering procedures, absolute intensity values, derived from each channel, are often used to calculate expression ratios for downstream statistical analyses. The expression ratios are a relative measure of transcript abundance within the two samples for each of the probes on the DNA microarray and are utilized to determine differential expression of genes between parasite samples. While direct hybridization of two different schistosome cDNA samples on the same slide has been routinely performed (e.g. [7]), indirect comparison through the co-hybridization of a test sample with a common or ‘universal’ reference standard is becoming more widespread (e.g. [8]). The value of a common reference sample is in facilitating the comparison of expression ratios across datasets and across samples when large (multiple sample), pair-wise, two-channel experiments are performed. Universal cDNA references derived from stoichiometric equivalents of all RNA samples being compared in an individual experiment (e.g. [9]) or created from a well-chosen panel of unrelated cell lines (e.g. [10]) or tissues (e.g. [11]) have all been utilized, but the lack of a single standard has hindered cross-comparison of studies, especially those involving schistosome parasites. Two limiting factors impeding the common adoption of a universal schistosome cDNA reference are the lack of parasite cell lines (from which material could readily be obtained) and the availability of parasite material for hybridization. Schistosome life cycles are difficult to maintain (requiring two different hosts), the parasites are relatively small (requiring large numbers for sufficient RNA) and *in vitro* culture of certain life-stages is laborious, with day-to-day reproducibility being highly dependent upon maintaining uncompromising culturing conditions. These factors make it extremely difficult for any research group to reproducibly generate the quantities of parasite cDNA required for maintenance of a universal reference to enable long-term data comparisons across experiments and importantly between laboratories.

Recent studies have demonstrated the feasibility of using gDNA as a universal reference for both prokaryotic and eukaryotic two-channel DNA microarray hybridization experiments [12],[13],[14],[15]. The attraction of exploiting this material as a universal schistosome reference is evident. Schistosome gDNA is relatively easy to obtain in large quantities, is more stable than RNA and is less prone to biological or experimental variations during its isolation that could directly affect microarray data acquisition and interpretation. It was, therefore, hypothesized that schistosome gDNA would be a superior reference to a mixed cDNA pool (derived from seven distinct *S. mansoni* life-stages) during indirect DNA microarray hybridizations.

Utilizing a new long-oligonucleotide *S. mansoni* DNA microarray of 37,632 elements, we demonstrate here that schistosome gDNA displays superior hybridization characteristics compared to a complex pool of schistosome cDNA. Importantly, we were able to exploit these qualities to confidently, and for the first time, identify 2,648 gender-associated transcripts in the morphologically identical, but chromosomally distinct, cercariae life-stage. Amongst numerous novel observations, male-associated transcription profiles were linked to processes including cytoskeletal or muscle organization, homotypic adhesion, male germ cell development and immune response modulation. In contrast, female-associated transcripts were represented by numerous repetitive or mobile genetic elements and some egg-associated proteins. Together, these transcriptional results suggest that morphologically identical cercariae have gender-associated patterns of gene expression already established and subsets of these genes are: **1)** necessary for the development of functionally distinct and dimorphic adults, **2)** likely instrumental in explaining male-biased sex ratios commonly observed during definitive host infection and **3)** possibly associated with processes involved in maintaining long-term female genomic stability.

## Methods

### Parasites

A Puerto Rican strain of *S. mansoni* was used in this study. All procedures performed on mice in these studies adhered to the United Kingdom Home Office Animals (Scientific Procedures) Act of 1986. Mixed-sex worms were perfused from percutaneously infected TO (Tuck Ordinary) mice (Harlan, UK) challenged either 3-wk (immature worms) or 7-wk (mature worms) earlier with 250 cercariae [16]. Cercariae were shed from *Biomphalaria glabrata* intermediate snail hosts. Schistosomula (18 hr) were prepared by mechanical transformation [17] and cultured at 37°C in DMEM (Sigma, UK) supplemented with 10% fetal calf serum, 2 mM *L*-glutamine, and 100 µg/ml penicillin/streptomycin in an atmosphere of 5% CO_2_ for the timeframe indicated. Lung-stage schistosomula were obtained from TO mouse lungs six-days after infection with 2,500 cercariae. Miracidia used to infect albino *B. glabrata* were hatched from eggs collected from TO mouse livers seven weeks after infection. Mother sporocysts were obtained from infected *B. glabrata* snails as previously described [18] and provided by Dr. Timothy Yoshino (University of Wisconsin, USA). Single-sex male cercariae were obtained upon shedding *B. glabrata* infected with a single male miracidium as previously described [19]. Single-sex female cercariae were isolated by an analogous method using snails exposed to a single female miracidium. *S. japonicum* adult worms (Philippine strain) were obtained from The Danish Bilharziasis Laboratory after being passaged through *Oncomelania hupensis* snails and NMRI mice as previously described [20].

### Genomic DNA isolation

*S. mansoni* genomic DNA (gDNA) was prepared from mixed-sex cercariae using a commercially available kit (Qiagen DNeasy tissue kit). *M. musculus* gDNA was prepared either from spleens of uninfected TO mice (6–8 wks old) using the Qiagen DNeasy tissue kit or purchased commercially (Strategene, UK). *S. japonicum* gDNA was prepared from mixed-sex Philippine strain adults using the Qiagen DNeasy tissue kit. All gDNA was qualitatively assessed by agarose gel electrophoresis and quantitatively measured using a NanoDrop ND-1000 UV-Vis spectrophotometer.

### Total RNA isolation

*S. mansoni* total RNA was isolated as previously described [21]. Briefly, parasite life-stages were homogenized in TRIzol reagent (Sigma, UK) using an Ultra-Turrax T8 dispersing tool (IKA-Labortechnik, Janke & Kunkel, GmbH & Co). Isolated total RNA was subsequently treated with DNAse I (Ambion, UK) to remove contaminating gDNA. All RNA was quantified utilizing a NanoDrop ND-1000 UV-Vis spectrophotometer and visualized for quality/contamination using an Agilent 2100 bioanalyzer.

### Construction of long-oligonucleotide DNA microarray

All *S. mansoni* sequences held in Genbank/EMBL as of May 2005 (152,749 EST- and 494 full length cDNA- sequences) together with 11,739 unpublished *S. mansoni* lung stage EST sequences from The Wellcome Trust Sanger Institute [22] were assembled using the CAP3 assembly package [23] with default parameter settings. This analysis identified a 37,918 element, non-redundant (NR) sequence set (13,339 contigs and 24,579 singletons). Since oligonucleotides synthesized for DNA microarrays are 50-mers, any sequences less than 50 bases were then removed from the original dataset, leaving 37,659 sequences. It is important to note that many of these NR sequences probably represent non-overlapping portions of the same gene or alternative splice variants and therefore, offers an explanation for why there is a large number of clusters (in relation to the predicted gene complement [24]) describing the parasite's expressed sequence repertoire.

To determine which sequences were already represented as 50-mers in our previous long-oligonucleotide DNA microarray library (EBI accession number: A-MEXP-134 [21]), each oligonucleotide in our existing set was compared to every sequence in the NR clustered sequence set. From this analysis, 6,388 sequences were identified as matching the existing oligonucleotides, with 31,271 not matching. However, as part of the process, 1,093 oligonucleotides matched more than one sequence in the NR dataset. Inspection of multiple sequences in the NR dataset matching single oligonucleotides indicate that some represent alternately-spliced transcripts (which, correctly, should not be assembled together), whilst others appear to represent minor single nucleotide polymorphisms (SNPs) or sequencing error-based variation, which should be assembled together, but were not due to the strictness of the CAP3 assembly. In order to assess the likely effect of such minor sequence variation on the true size of the NR sequence set, we re-assembled the 31,271 sequences from CAP3 using TGICL [25]. This analysis generated 28,863 unique sequences (27,333 singletons and 1,530 contigs).

At most four oligonucleotide representations for each final *S. mansoni* clustered sequence were identified by a series of iterative procedures, previously used successfully for the selection of the current 7,335 *S. mansoni* oligonucleotide set [21]. The T_m_ of these possible oligonucleotides was determined using the program ‘dan’ on The European Molecular Biology Open Software Suite (EMBOSS [26]), and the oligo representations of each parent sequence with a T_m_ closest to 72°C were initially selected. If multiple oligos were selected on this basis then those with the most unique BLASTn score (when compared to the complete 50-mer set) were subsequently selected. If multiple oligos were selected on this basis, then that with the highest % GC content was ultimately selected for synthesis (Invitrogen, UK).

Of the 27,333 singletons, 26,576 sequences passed all criteria and 50-mer oligo representations (6-C linked 5′ amino modification, 50 nmol scale) were synthesized. Similarly, of the 1,530 contigs, 1,369 sequences passed the same criteria and 50-mer oligo representations were additionally synthesized. These newly selected *S. mansoni* 50-mer oligonucleotides, along with 7,492 previously synthesized ones [21] and control elements (2,195: comprising 768 70-mer non *S. mansoni* oligonucleotides obtained from the Centre for Microarray Resources, Department of Pathology, University of Cambridge and 1,427 non-DNA elements [buffer]), were printed on CodeLink Activated Slides (Amine-Binding Slides) (Amersham Biosciences, UK) at a concentration of 250 ngµl^−1^ by the Centre for Microarray Resources. The DNA microarray, therefore consists of 37,632 elements (35,437 *S. mansoni* elements and 2,195 controls) and has been deposited in EBI's ArrayExpress database under the accession number A-MEXP-830. All *S. mansoni* 50-mers have been aligned against the latest gene models (genome annotation v4) and the current genome scaffold (genome assembly v3.1) using Exonerate [27]. Sequences of each 50-mer and positions of stringent alignment on the *S. mansoni* genome can be visualized using the *Schistosoma mansoni* genome browser at GeneDB [22].

### DNA microarray hybridization

AlexaFluor647/AlexaFluor555-dCTP (Amersham Biosciences, UK) labelled cDNA targets were generated through a modified version of the procedure first described by Petalidis *et al.* [28]. Five hundred ng of *S. mansoni* total RNA was used in a mRNA amplification reaction using template-switching PCR. Optimal PCR cycle number was established empirically by evaluating yield of PCR product with increasing cycle number. Amplified cDNA or 400 ng un-amplified gDNA was labelled overnight at 37°C, re-suspended in hybridization solution (5× SSC, 5× Denhardt's solution, 1 mM sodium pyrophosphate, 50 mM Tris (pH 7.4) and 0.1% SDS) and denatured at 95°C for 5 min then held at 50°C for a further 5 min. Microarray hybridization was performed in a humidified chamber at 45°C for 16–18 h. Three successive, post-hybridization stringency washes were performed at room temperature for 3-min/wash, with agitation (2× SSC, 0.1× SSC/0.1% SDS and 0.1× SSC respectively). Image acquisition (16-bit tiff) for the DNA microarray was performed using a GenePix 4100A (Axon Instruments Inc.) dual channel laser scanner at 10 µm resolution, 100% laser power and photomultiplier tube gain levels ranging from 580 to 730. BlueFuse for Microarrays (BlueGnome Ltd., UK) image analysis software was used to extract fluorescent signal intensity data.

### Male cercariae vs female cercariae data analysis (direct method for determining gene expression)

Gender-associated cercarial transcripts were identified by analyses utilized in previous studies of gender in adult schistosomes [21]. Poor quality spots and low intensity data were filtered and removed by a succession of applied statistical criteria. Initially, the arithmetic mean was calculated for all non-*S. mansoni* control elements (2,195 negative controls including non *S. mansoni* control sequences and blank/buffer elements). The mean signal intensity for each hybridized *S. mansoni* element was required to be greater than one standard deviation above the mean of the negative controls in at least one channel (AF555 or AF647). All data below this value for each independent experiment were excluded from further analysis. Oligonucleotides passing these filtering criteria had to display natural Log normalized expression values outside of the 90% confidence interval in at least three out of five (including dye-swap experiments) replicate DNA hybridizations to be included in the final list of differentially expressed, gender-associated transcripts.

### Genomic DNA data analysis (indirect method for determining gene expression)

Each scanned DNA microarray was manually inspected for low quality spots and these elements (along with the 2,195 negative control elements) were removed from further analyses. All remaining spots were utilized for data analysis. All gDNA (AF555) co-hybridized with cercarial cDNA (AF647) were normalized by dividing the median fluorescent gDNA intensity (n = 3) obtained from hybridization to an individual 50-mer by the value of the fluorescent cDNA intensity obtained from the hybridization to the same 50-mer oligonucleotide in each single experiment. Student's *t-test* (n = 3, *p* = 0.01) was used to identify gender-associated cercarial transcripts (indirectly via the reference gDNA). Correlation coefficients (R) for biological replicate hybridizations were derived from a goodness of fit measure of a linear model where values approaching one indicate a high degree of agreement. Box-plots were generated for median signal intensity values and display the intensity values for 25% of the oligonucleotides above and below the median. The whiskers show those oligonucleotides lying within 1.5 deviations of the median and the outliers are beyond 1.5 deviations.

All microarray data is MIAME compliant [29] and has been submitted to ArrayExpress at the European Bioinformatics Institute, Hinxton, UK (Array accession number, A-MEXP-830, experiment accession number, E-MEXP-1259) [30].

### Gene ontology classification of differentially expressed transcripts

Transcripts identified as differentially expressed by both DNA microarray hybridization strategies (156 Female and 188 Male, see below) were submitted to GOblet [31] for autoannotation with Gene Ontology classifications (using GOblet's invertebrate database and *E* value cutoff score of 1E^−10^). The percentage of transcripts falling into various Molecular Function, Biological Process and Cellular Composition categories were collated and depicted as bar graphs.

### Real time PCR analysis

Relative gene expression (fold difference between samples) was quantified relative to alpha-tubulin [32] using a MiniOpticon Real-Time PCR Detection System (Bio-Rad) and SYBR green chemistry (Bio-Rad). Total reaction volume was 25 uL using 250 nM of each primer (sequences provided in Dataset S1) and 2 uL of 1/10 dilution from the original cDNA microarray targets (before labeling). PCR efficiency (E) of each primer pair was determined by plotting cycle thresholds from a 10-fold serial dilution of cDNA (1/10, 1/100 and 1/1000 dilutions) and inputting the slope in the equation E = 10^(−1/slope)^. The fold difference of each transcript (between gender) was calculated relative to alpha-tubulin using the Pfaffl equation [33]:
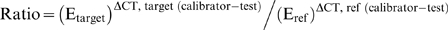
where E_target_ is the PCR efficiency of the target gene, E_ref_ is the PCR efficiency of reference gene (alpha-tubulin), CT is the cycle threshold, calibrator represents one cercariae gender and test represents the other gender. Melting curves were generated for each real-time PCR reaction in order to verify the amplification of only one product. All amplicons were subcloned in appropriate vectors (PCR2.1 or PCR4.0, Invitrogen) and sequenced at the Department of Genetics, University of Cambridge in both orientations using Big Dye v3.1 fluorescent chemistry and an Applied Biosystems 3100 Genetic Analyser in order to confirm their identity.

## Results

### Construction of a *S. mansoni* long-oligonucleotide DNA microarray composed of 37,632 elements

A new long-oligonucleotide DNA microarray was created from *S. mansoni* sequences held in Genbank/EMBL as of May 2005 (152,749 EST- and 494 full-length cDNA- sequences) together with the 11,739 unpublished *S. mansoni* lung stage EST sequences from The Wellcome Trust Sanger Institute (WTSI, [34]). After sequence clustering (CAP3 [23] and TGICL [25]), sequence filtering (*Methods* and [21]) and sequence comparison to the 7,492 long-oligonucleotides (50-mers) previously created for the first generation DNA microarray [21], a selection of 27,945 new *S. mansoni* 50-mers were produced for this second generation long-oligonucleotide DNA microarray (Table 1). Additional elements selected for printing include 768 non-*S. mansoni* sequences (comprised of *M. musculus*, *Arabidopsis thaliana*, *Methanococcus jannaschii*, *Escherichia coli* and *Bacillus subtilis* 70-mers) and 1,427 buffer controls (Table 1). All 27,945 new *S. mansoni* sequences (from which 50-mers were produced) were submitted to BLASTx analysis at the NCBI and the top five hits were extracted. This led to the annotation of 12,337 sequences (44%) that contained significant database similarity (defined as *E*≤10^−05^) and 15,608 sequences (56%) displaying no significant database similarity (Table 1). As the previously synthesized 7,335 oligonucleotides were annotated during prior bioinformatics analyses [21], they were not resubmitted for further BLASTx database queries. All 35,437 *S. mansoni* 50-mers and 768 non-*S. mansoni* 70-mers were covalently attached, via 5′ 6-C amino modifications, to amine binding Codelink slides. Together with 1,427 buffer controls, this second generation *S. mansoni* long-oligonucleotide DNA microarray contained 37,632 elements. Although a degree of redundancy (multiple 50-mer oligonucleotides corresponding to non-overlapping clusters or alternative transcripts from the same *S. mansoni* gene) is expected within the new DNA microarray sequence-set, the exact level is presently unknown. Completion of *S. mansoni* genomic sequence annotation will resolve this issue. All 50-mer *S. mansoni* oligonucleotides have now been aligned against the latest gene models and the current genome scaffold (each 50-mer sequence and its location on the genome are visible using the genome browser on SchistoGeneDB [22]) to facilitate linkage between transcriptomic data and genome assembly.

**Table 1:** *S. mansoni* long-oligonucleotide DNA microarray information.

Total number of microarray elements	***37632***
Total number of oligonuceotide elements designed from *S. mansoni* sequences	*35437*
Number of existing oligonucleotides from 1st microarray [21]	*7492*
Number of new oligonucleotides	*27945*
Number of non *S. mansoni* control sequences	*768*
Number of buffer/negative control elements	*1427*
Total number of new *S. mansoni* sequences submitted for BLASTx analysis	*27945*
Sequences (44%) displaying significant similarity (expect value≤10^−5^)	*12337*
Sequences (56%) displaying no significant similarity	*15608*
Total number of old *S. mansoni* sequences previously submitted for BLASTx analysis from 1st microarray	*7335*
Sequences displaying significant similarity to identified proteins (expect value≤10^−5^)	*2714*
Sequences displaying significant similarity to hypothetical proteins (expect value≤10^−5^)	*3601*
Sequences displaying no significant similarity	*4621*

### *S. mansoni* gDNA binds to long-oligonucleotide DNA microarrays with species specificity and generates fluorescent signal intensity of greater value and less variation than a complex pool of cDNA

**Figure 1:**
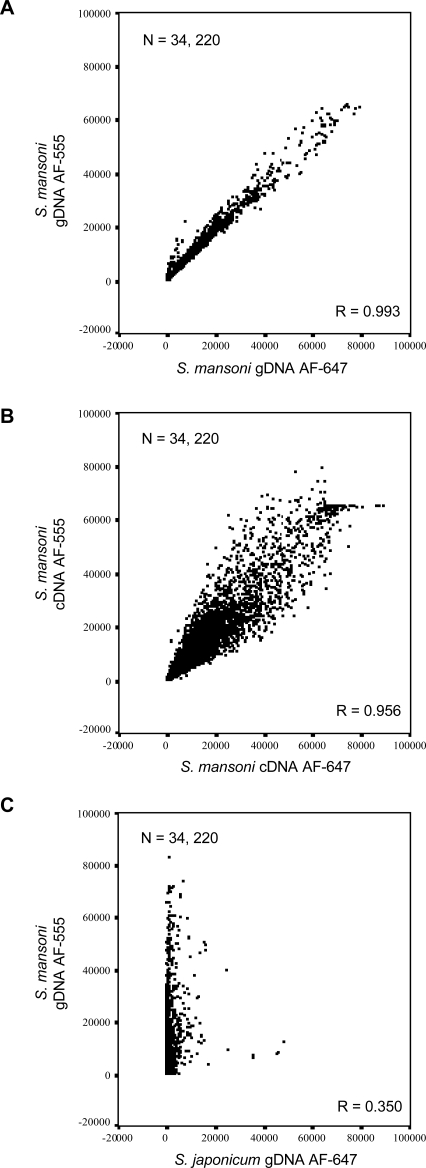
Long-oligonucleotide DNA microarray is specific for *S. mansoni* nucleic acid material. *S. mansoni* gDNA hybridizes to the oligonucleotide DNA microarray with greater specificity and less variation compared to *S. japonicum* gDNA or a mixed pool of *S. mansoni* cDNA. Scatter plots display signal intensity for all oligonucleotides passing manual exclusion filters in all three displayed experiments. Correlation coefficient values for each comparison, R = 0.993, 0.956 and 0.350 indicate a high degree of correlation between AF555 and AF647 signal intensities from gDNA and cDNA, but not from *S. mansoni* AF555 labeled gDNA compared to *S. japonicum* AF647 labeled gDNA. A) Scatter plot for AF555 labeled *S. mansoni* gDNA compared to AF647 labeled *S. mansoni* gDNA signal intensities. B) Scatter plot for *S. mansoni* AF555 labeled cDNA compared to *S. mansoni* AF647 labeled cDNA (as described in *Methods*) signal intensities. C) Scatter plot for *S. mansoni* AF555 labeled gDNA compared to *S. japonicum* AF647 labeled gDNA signal intensities. All negative control microarray signals were removed before analysis, microarrays were filtered for manually excluded spots and each experiment was subsequently filtered to contain the same number of spots (n = 34,220).

To address the utility of using *S. mansoni* gDNA as a suitable reference during two-channel DNA microarray hybridization experiments, the binding characteristics of this nucleic acid to the newly created DNA microarray were ascertained. A series of experiments were thus performed to test the specificity and binding capacity of *S. mansoni* gDNA as compared to those of *S. japonicum* gDNA and *M. musculus* gDNA as well as another commonly used reference, a complex pool of *S. mansoni* cDNA (created here from stoichiometric equivalents of cercariae, 18-hr cultured schistosomula, 6-day lung-stage worms, miracidia, mother sporocyst, 3-wk worm and 7-wk worm RNA). First, co-hybridization of two mixed-sex *S. mansoni* gDNA samples (one labelled with AF-555 and the other with AF-647) to the DNA microarray was contrasted to the co-hybridization of two *S. mansoni* cDNA complex pools (one labelled with AF-555 and the other with AF-647) to the DNA microarray (Fig. 1). Although *S. mansoni* cDNA hybridized to the DNA microarray with good characteristics (correlation coefficient R = 0.956, Fig. 1B) during these ‘self on self’ hybridizations, *S. mansoni* gDNA outperformed the complex cDNA reference pool with a correlation coefficient approaching 1 (R = 0.993) (Fig. 1A). When *S. japonicum* gDNA was co-hybridized with *S. mansoni* gDNA, the correlation coefficient dropped dramatically (R = 0.350) (Fig. 1C), suggesting that the DNA microarray is species specific and that related *S. japonicum* gDNA did not appreciably bind to *S. mansoni* 50-mer oligonucleotides.

**Figure 2:**
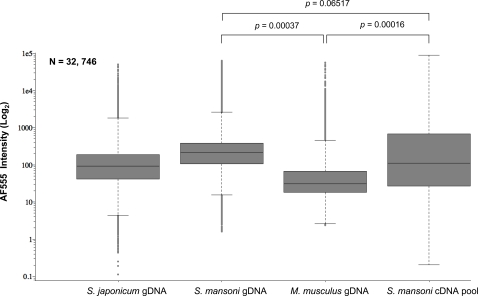
*S. mansoni* gDNA has superior hybridization characteristics compared to *S. mansoni* cDNA. The use of *S. mansoni* gDNA as a common reference reveals higher median intensity values and narrower overall intensity distributions than a complex *S. mansoni* cDNA reference. Box-plots display signal intensities for the AF-555 channel for *S. japonicum*, *S. mansoni*, *M. musculus* gDNA and complex reference *S. mansoni* cDNA pool (as described in *Methods*). Boxes represent three independent experiments for mouse gDNA, *S. mansoni* gDNA and *S. mansoni* cDNA pool, respectively. *S. japonicum* gDNA is represented by a single experiment. All negative control microarray signals were removed before analysis, microarrays were filtered for manually excluded spots and each experiment was subsequently filtered to contain the same number of spots (n = 32,746). Statistical significance was defined using a Student's *t-test* between three independently replicated experiments. Horizontal lines represent median oligonucleotide intensity, boxes display oligonucleotide elements 25% above and below the median value and whiskers show oligonucleotide elements within 1.5 deviations of the median. Outlier oligonucleotide intensity values (beyond 1.5 deviations) are illustrated as individual spots.

Focusing additional analyses on one fluorescent channel (AF-555) during these two channel hybridization experiments also illustrated that *S. mansoni* gDNA binds to the DNA microarray with a greater affinity and less variation than the complex cDNA reference pool (Fig. 2). The median fluorescent intensity of bound *S. mansoni* gDNA (209.9) labelled with AF-555 was higher than that of bound *S. mansoni* cDNA (112.2) labelled with the same fluorochrome. Additionally, the majority of acquired fluorescent data (25% above and below the median) was less variable (tighter box plot) than that observed for bound *S. mansoni* cDNA (wider box plot). Furthermore, the range of oligonucleotide elements within 1.5 deviations of the median was narrower (whiskers) for AF-555 values obtained from hybridization experiments using *S. mansoni* gDNA when compared to hybridization experiments using *S. mansoni* cDNA pools. This was also true for outlier oligonucleotide intensity values (beyond 1.5 deviations of the median) (Fig. 2). These comparisons approached statistical significance (*p* = 0.065) and suggested that *S. mansoni* gDNA had improved hybridization characteristics (higher, less variable, overall fluorescent intensities) when compared to a complex, mixed pool of *S. mansoni* cDNA. *S. mansoni* gDNA labelled with AF-647 (dye-swap experiments) also demonstrated these superior properties (data not shown).

Neither AF-555 labelled *M. musculus* gDNA or *S. japonicum* gDNA hybridized to the DNA microarray with strong affinity (Fig. 2). This further supports the results obtained in Fig. 1 regarding the relative lack of detectable *S. japonicum* gDNA fluorescent signal intensity when co-hybridized with *S. mansoni* gDNA. In the specific case of *M. musculus* gDNA, median AF-555 fluorescent intensity values were significantly lower (30.8) than those recorded with either *S. mansoni* gDNA or complex cDNA reference material (*p* = 0.00037 and *p* = 0.00016, respectively). Together, these results suggest that the developed DNA microarray resource is specific for *S. mansoni* nucleic acid material and that gDNA is better suited than a complex, mixed pool of cDNA for use in two-channel DNA microarray hybridization experiments.

### Gender-associated transcription profiles are clearly established in morphologically identical, but chromosomally distinct, *S. mansoni* cercaraie

**Figure 3:**
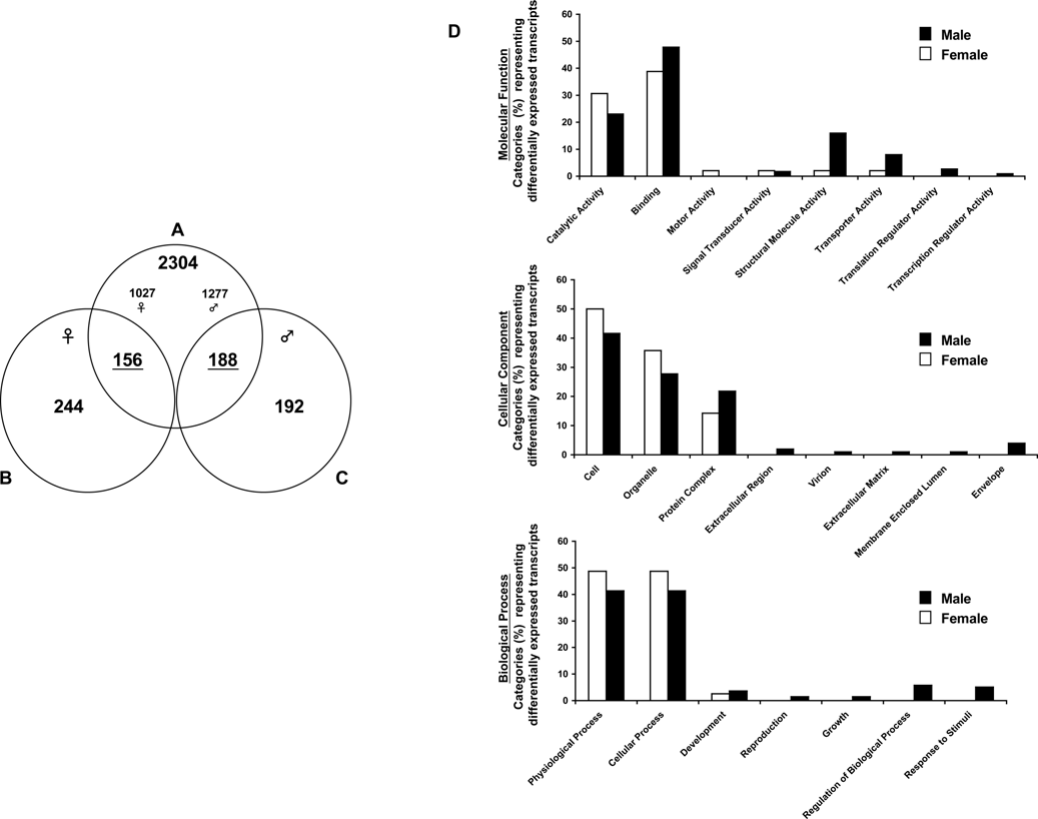
Increased number of gender-associated, differentially expressed transcripts are identified using *S. mansoni* gDNA as a common reference. Venn diagram demonstrates numbers of differentially expressed gender-associated cercarial transcripts as revealed by utilization of two different DNA microarray hybridization strategies. A) represents an indirect method for calculating gene expression ratios where gender-associated transcripts were identified through a statistical Student's *t-test* comparison of male cDNA v reference gDNA (n = 3) and female cDNA v reference gDNA (n = 3). B) and C) both represent direct methods for calculating gene expression ratios as identified by analysis of five independent experiments of male cDNA v female cDNA targets in both dye combinations. B) denotes female-biased transcripts identified. C) represents male-biased transcripts. All differentially expressed transcripts identified here are listed in the Dataset S2. D) Lists of the concordant, differentially expressed male (188) and female (156) transcripts were analyzed by Gene Ontology classification and the percentages of transcripts defined by Molecular Function, Cellular Composition and Biological Process categories are illustrated.

**Figure 4:**
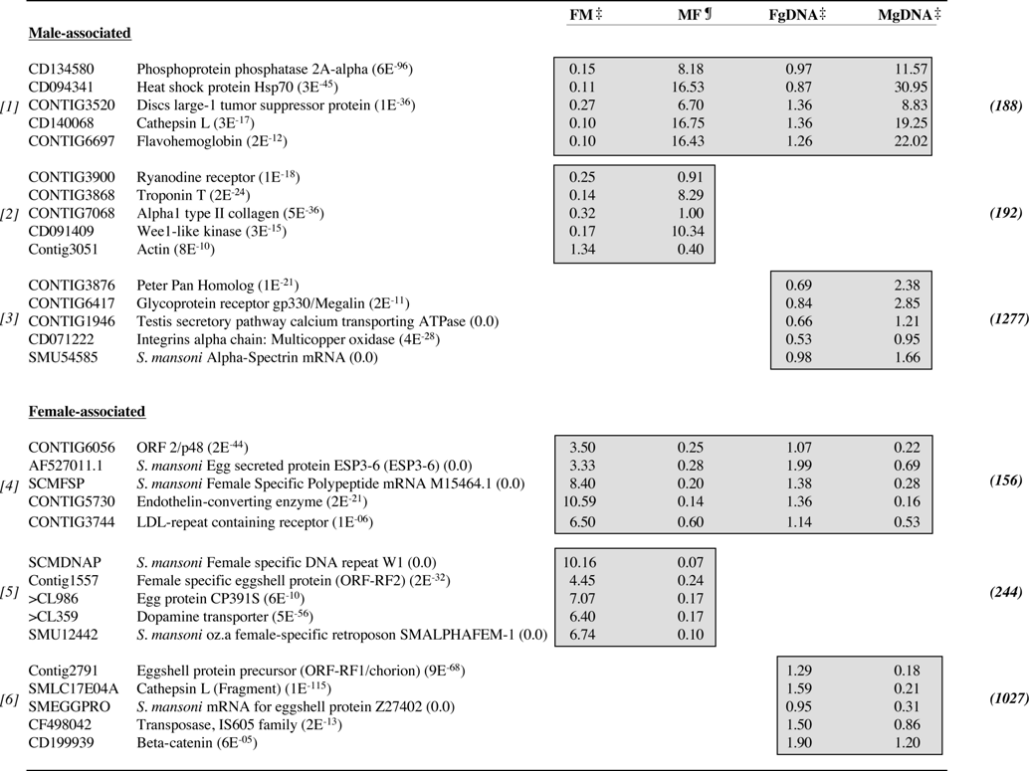
Representation of some differentially expressed, gender-associated cercariae transcripts, as identified through two distinct hybridization methods. Numbers in parentheses represent BLASTx NCBI nr database annotation, numbers in bold parentheses represent the total number of transcripts identified as differentially expressed using that particular method and relate directly to Fig. 3-Venn diagram of differentially expressed transcripts. Gray shading highlights differentially expressed ratios as identified through the statistical methods indicated. *[1]* Male-associated transcripts as identified through direct ratio comparison of male and female cercarial cDNA samples and additionally identified indirectly using gDNA as a common denominator-male cDNA v gDNA compared with female cDNA v gDNA. *[2]* Male-associated transcripts as identified though a direct comparison only. *[3]* Male-associated transcripts as identified though an indirect comparison only. *[4]* Female-associated transcripts as identified through direct ratio comparison of male and female cercarial cDNA samples and additionally identified indirectly using gDNA as a common denominator-male cDNA v gDNA compared with female cDNA v gDNA. Gray shading highlights differentially expressed ratios as identified through the statistical methods indicated. *[5]* Female-associated transcripts as identified though a direct comparison only. *[6]* Female-associated transcripts as identified though an indirect comparison only. FM: Female AF647 v Male AF555 cDNA; MF: Male AF647 v Female AF555 cDNA; FgDNA: Female AF647 cDNA v gDNA AF555; MgDNA: Male AF647 cDNA v gDNA AF555. Expression ratios within columns represent a mean AF657/AF555 ratio for all experiments. ‡ denotes mean of three replicate experiments, ¶ denotes mean ratio of two replicate experiments.

Direct DNA microarray hybridization strategies have been successfully used for identifying adult male- and female-associated schistosome transcripts [7],[20],[21],[35]. Whether other life-stages (e.g. cercariae or miracidia) also harbour detectable gender-associated expression profiles has not previously been addressed. Therefore, to identify gender-associated transcripts in the cercariae life-stage, we first applied the commonly used direct hybridization DNA microarray approach. Here, comparing the co-hybridization of male and female cDNA on the same DNA microarray, 400 transcripts were identified as differentially expressed in female cercariae whereas 380 transcripts were found differentially expressed in male cercariae (Fig. 3 and Dataset S2). However, when an indirect hybridization methodology was employed using gDNA as a common reference, significantly increased numbers of transcripts were identified as being differentially expressed in cercariae (total of 2,648 with a split of 1,465 male- and 1,183 female-associated gene products; Fig. 3 and Dataset S2). Interestingly, when data obtained by both hybridization strategies were compared, a concordant subset of gender-associated transcripts was found. Here, 156 female-associated transcripts and 188 male-associated transcripts (Fig. 3, Venn diagram overlap and Dataset S2) were identified from a union of data collected by both hybridization methods and analyses. Further examination of these specific transcripts by Gene Ontology nomenclature revealed a range of diverse classifications (Fig. 3 and Dataset S2). Relatively equal proportions of differentially expressed male and female transcripts were found for many of the Gene Ontology categories, however there were several categories (eg. Molecular Function-Structural Molecule Activity; Cellular Component-Extracellular region and Extracellular Matrix; Biological Process-Regulation of Biological Process and Response to Stimuli) that were dominated by male-associated transcripts. A subset of selected gender-associated cercarial transcripts (and the hybridization strategy by which they were identified) is listed in Fig. 4 with the complete dataset being found in Dataset S2.

Careful inspection of all identified female-associated transcripts led to some interesting observations. Firstly, many (12%) of the 1,183 transcripts identified through use of gDNA as an indirect common reference (Fig. 3) had database similarity with schistosome repeat (W1 and W2 [36],[37]) or mobile genetic elements (see Dataset S2, S4 and S5) but were sufficiently distinct from each other to not have assembled together in the clustering process. This also held true when data collected from direct (male cDNA vs female cDNA) microarray hybridizations were compared (Dataset S2, S4 and S5). Secondly, several transcripts previously found in the adult female that presumably are associated with egg biology [21] were also found to be differentially expressed in the non-egg laying, female cercariae. These gene products are represented by ORF-RF1/2 [38], egg protein CP391S, mucin-like protein [39], p48 [40], female specific protein FSP [41] and ESP3-6/IPSE/alpha-1 [42] (Fig. 4 and Dataset S2). Together with the multiple transcripts harbouring unknown or hypothetical database annotations, these gene products serve as a rich dataset providing novel insights into the biology and behaviour of female cercariae.

Amongst numerous male-associated gene products with ‘hypothetical’ and ‘unknown’ annotations, several transcripts having similarity to database entries were broadly aligned to biological categories including cytoskeletal (and muscle) organization (eg. fimbrin/plastrin-, troponin T-, troponin C-, intermediate filament like-, myosin heavy chain-, myosin XVIII-, myosin light chain kinase-, ankyrin-, spectrin- and myophilin/calponin/transgelin-like orthologs), male germ cell development (orthologs of testicular microtubule-related protein 4, cyclin A, and translin-associated factor X interacting protein) and immune response modulation (flavohemoglobin-like protein) (Fig. 4 and Dataset S2). In addition, transcripts associated with glucose transport (glucose transport protein 1 and glucose transport protein 4 [43],[44]) and storage (glycogenin [45]) as well as proteolysis (EOS serine protease and cathepsin L isoforms) were also found differentially expressed in male cercariae (Fig. 4 and Dataset S2). The biological role of these transcripts (and others) during definitive host invasion and male developmental processes remains unknown at this time, but demonstrates that gender-associated patterns of gene expression are already established in both sexes of this larval life-stage.

### Quantitative real time PCR analysis confirmed cercariae gender-associated transcription profiles

**Figure 5:**
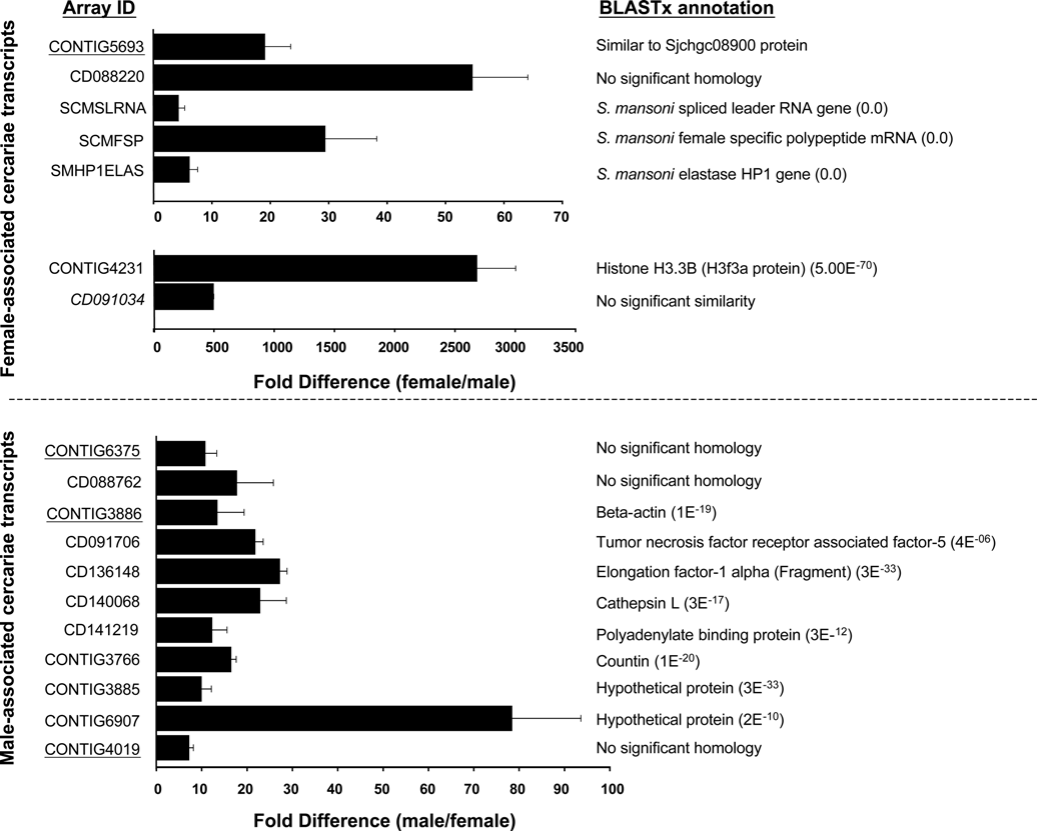
Real-time quantitative PCR analysis confirmed cercariae gender-associated DNA microarray results. PCR was performed using a MiniOpticon Real-Time PCR Detection System (Bio-Rad, UK) and SYBR Green I chemistry according to manufacturer's instructions. Briefly, real-time PCR parameters included 40 cycles, fluorescent reading after each cycle and melt curve analysis of individual products at the end of the 40 cycles. Unique oligonucleotide identification number is shown along with BLASTx annotation and gender association. Significant similarity is defined as (E) value≤10^−05^. Fold-difference was calculated as described in *Methods*. Primer characteristics (Tm, annealing temperature and sequence) along with amplicon size are included in the Dataset S1 (RT-PCR Primers). Underlined unique IDs represent gender-associated transcripts identified by the indirect hybridization strategy (parasite cDNA versus parasite gDNA). The *italicized* unique ID represents a gender-associated transcript identified by the direct hybridization strategy (female cDNA versus male cDNA). Plain text unique IDs represent gender-associated transcripts identified by both hybridization strategies.

Eighteen transcripts (Dataset S1) were randomly chosen for quantitative real time PCR analysis in order to confirm the gender-associated patterns of gene expression identified from the DNA microarray hybridization experiments (Fig. 5). Although differences in magnitude of expression were expected [46] and observed between results obtained from quantitative real time PCR compared to DNA microarray based methods, all eighteen transcripts displayed the expected gender association, regardless of the DNA microarray hybridization technique employed.

## Discussion

Standardization of DNA microarray hybridization protocols to facilitate inter-laboratory comparisons of global schistosome gene expression experiments is essential to make sense of the wealth of information originating from increased utilization of this technology. One of the most important experimental issues for investigators utilizing two-channel DNA microarray hybridization methods is the selection of a renewable, abundant and stable reference material for comparisons across experiments, within and outside their laboratory. Other experimental factors including RNA isolation, cDNA labelling, DNA microarray hybridization/washing and slide scanning are comparably less important and usually differ amongst laboratory groups. Data filtering and statistical analyses may also vary, but the availability of primary, MIAME-compliant [29] schistosome datasets (housed at EBI or NCBI) allows for comparisons to be made using identical statistical criteria. Therefore, provided that a similar hybridization methodology and a common reference material are employed during two-channel DNA microarray experiments, cross-dataset comparisons can be performed, thereby increasing the power of this technology for generating new insights into schistosome biology. Here, we demonstrate that schistosome gDNA fulfils many requirements for utilization as a standard reference material in two-channel DNA microarray experiments and additionally show its usefulness in identifying gender-associated transcription profiles in morphologically identical, but chromosomally distinct cercariae.

Schistosome gDNA is an easily obtainable reference material and was, therefore, examined for its suitability to hybridize to long-oligonucleotide *S. mansoni* DNA microarrays. During these experiments, we additionally determined whether gDNA isolated from other organisms (*S. japonicum* and *M. musculus*) was capable of hybridizing to the newly generated *S. mansoni* DNA microarray. All of these gDNA materials were compared to the hybridization characteristics of a mixed schistosome cDNA pool, which served as a representative, complex reference during two-channel schistosome DNA microarray hybridization experiments. The findings from these experiments (Figs. 1 and 2) conclusively demonstrated that *S. mansoni* gDNA hybridized to the newly constructed *S. mansoni* DNA microarray with much better characteristics than any of the other tested nucleic acid materials. These characteristics included: 1) higher correlation coefficients when ‘self vs self’ hybridization experiments were employed, 2) increased median fluorescent intensities across all arrayed elements (compared to all nucleic acid material) and 3) decreased spread of outlier fluorescent intensity values obtained from all arrayed elements (when compared to the mixed pool of *S. mansoni* cDNA). Along with the ease with which gDNA can be obtained, these characteristics make *S. mansoni* gDNA a greatly superior reference alternative compared to a mixed pool of cDNA during two-channel schistosome DNA microarray hybridization experiments. Arguably, it is possible that if the cDNA pool contained a greater life-stage representation (e.g. egg and other schistosomula stages), the hybridization characteristics between *S. mansoni* gDNA and cDNA might be more similar. Keeping in mind the difficulties in obtaining sufficient RNA from these life-stages, even a modest increase in hybridization characteristics would not justify the effort/animals required to generate enough material for multiple, replicate hybridization experiments.

It is interesting to note that, while most long-oligonucleotide DNA elements failed to hybridize to either *S. japonicum* or *M. musculus* gDNA with high affinity (Figs. 1 and 2), some elements did hybridize with a degree of selectivity. Fluorescent dye biased effects can be ruled out here as the majority of *S. japonicum* and *M. musculus* outliers in the AF-555 channel are also present in the outliers of the AF-647 channel (96% and 99% respectively, data not shown). Therefore, these outliers likely represent sequence conservation between the *S. mansoni* 50-mer long-oligonucleotide and *S. japonicum*/*M. musculus* gDNA or poor-quality arrayed elements that escaped filtering (e.g. merged arrayed elements or non-specific auto-fluorescence). Careful inspection of the highly fluorescent outliers in the *S. japonicum* (Fig. 2) sample revealed that 33% had database annotation to *S. mansoni* mobile genetic or repetitive elements (data not shown). High sequence conservation between certain *S. mansoni* and *S. japonicum* mobile genetic elements (e.g. SR-1 from *S. mansoni* with pido from *S. japonicum* [47]) supports our interpretation that many of these highly fluorescent outliers cross-hybridize due to sequence similarity.

Taking advantage of the characteristics associated with using gDNA as an indirect reference during two-channel DNA microarray experiments, we identified (for the first time) gender-associated patterns of gene expression in morphologically identical, but chromosomally distinct, *S. mansoni* cercariae. As we have previously examined gender-associated transcription between sexually mature, dimorphic adult schistosomes using direct DNA microarray hybridization techniques [7],[20],[21], we decided to compare strategies (direct versus indirect) to obtain novel biological insight for the larval cercarial life-stage. Supporting previous DNA microarray cross-comparative investigations [48],[49], different unique sets of gender-associated transcripts were identified in cercarial samples when distinct hybridization strategies and statistical methods were employed (Fig. 3, Fig. 4 and Dataset S2). This strongly supports our argument that standardization of schistosome two-channel DNA microarray experiments is absolutely essential when cross-dataset comparisons are envisioned or required. However, this does not lessen the impact or validity of those differentially expressed genes identified by the two distinct hybridization strategies employed in this study (full list in Dataset S2). Additional confirmatory experiments would be required before advanced biological investigations are initiated, but this would be the case for any specific, differentially expressed transcript identified from global gene expression studies (regardless of the hybridization strategy employed).

While large numbers of non-overlapping, differentially expressed cercarial transcripts were identified by the two distinct hybridization strategies, concordant lists of gender-associated gene products were also detected in this study (156 female and 188 male, Fig. 3 and Dataset S2). This finding was not dependent on transcript abundance as these lists included gene products that were both highly expressed (e.g. CD091706, CD136148 and CD140068, CD088220, CD091034 and CONTIG4231) and weakly expressed (e.g. CONTIG6375 and CONTIG3886), based on fluorescent intensity. These concordant lists of differentially expressed transcripts (several confirmed by real time PCR analysis, Fig. 5) likely represent those displaying the most divergent gender-associated patterns of expression in the cercariae and may be useful in transcriptional characterization of this life-stage.

Together, assimilation and interrogation of all gender-associated cercarial transcripts identified by both DNA microarray hybridization technologies provided some interesting information related to the biology of this free-living, definitive host life-stage. These data reveal that cercariae, surprisingly, do express gender-associated gene repertoires (across diverse Molecular Function, Biological Process and Cellular Component Gene Ontology categories, Fig. 3). In the specific case of female cercariae, our findings demonstrate that several transcripts involved in egg biology were detectable; these included ORF-RF1/2 [38], egg protein CP391S, mucin-like protein [39], p48 [40], female specific protein FSP [41] and ESP3-6/IPSE/alpha-1 [42] (see Dataset S2 for complete list). As female cercariae do not produce eggs, transcription of these egg-associated genes (as well as others putatively involved in maturation, i.e. LDL-repeat containing receptor (CONTIG3744), Fig. 4) suggests that female parasites harbour the capacity to transcribe a steady-state subset of genes in preparation for further developmental/maturation signals.

Male cercariae also differentially transcribe (in comparison to female cercariae) a subset of genes that have previously been found in adult male worm expression profiles [7],[20],[21]. Structural transcripts in this category (subset listed, full details in Dataset S2, also illustrated in Fig. 3, Molecular Function Category) include putative troponin T-, troponin C-, intermediate filament like-, myosin heavy chain-, myosin light chain kinase-, fimbrin/plastin-, myosin XVIII-, ankyrin-, spectrin- and myophilin/caloponin/transgelin orthologs. Interesting, we also demonstrate the male-dominated expression of the gynecophoral canal protein [50], a developmentally regulated molecule putatively involved in homotypic adhesion processes between adult male and female worms. As adult male schistosomes provide a structural/supportive role for adult females during conjugal biology [51], differential expression of these genes in male cercariae may ensure that the necessary components are present at some basal level.

The transcription of genes involved in male germ cell development and reproduction (Biological Process, Fig. 3 and Dataset S2, eg. testicular microtubule-related protein 4 [52], cyclin A1 [53] and translin-associated factor X interacting protein [54]) also suggests that the process of male sexual maturation may be initiated as early as the cercarial life-stage. The operative mechanism responsible for these male-associated transcriptional profiles in the cercariae is currently unknown. However, transcription of these particular gene subsets suggests that male cercariae already harbour pre-defined regulatory mechanisms necessary for the development of adult male-specific roles leading to the successful initiation of conjugal interactions.

Male-biased sex ratios have been reported during experimental and natural schistosome infections with a range of hypotheses accounting for this observation subsequently being experimentally tested [55]. An extensive study in *S. mansoni* demonstrated that this biased sex ratio could be explained by greater male infectivity and survival in intermediate and definitive hosts [55]. Our male-associated gene expression profiles offer some support for this explanation. Specifically, transcripts associated with glucose transport (SmGTP1 and SmGTP4 [43]) and storage (CONTIG5503, glycogenin [45]) as well as immune response modulation (CONTIG6697, flavohemoglobin-like protein) could contribute to greater male infectivity and survival in definitive hosts.

The transcription of SmGTP1 and SmGTP4 in cercariae has been described [43], with our results now adding a male bias to this observation. SmGTP1 and SmGTP4 encode functional glucose transporters [43] and are likely responsible for trans-tegumental transfer of glucose in the developing schistosome. Their differential transcription in cercariae (as well as adults [21]) could prepare the male parasite for greater utilization of definitive host glucose immediately upon infection, which would facilitate more rapid and sustained developmental processes. Indeed, SmGTP4 translation is dramatically up-regulated one hour after cercariae transform into schistosomula [44] and suggests that the SmGTP4 transcripts already present in cercariae contribute to the rapid presence of functional protein essential for energy production mediated by glycolysis [56].

The differential expression of glycogenin in male cercariae also supports the ability of this gender to utilize glucose for energy production at a greater capacity than female cercariae. Glycogenin is the priming glycosyltransferase responsible for glycogen storage in eukaryotes [45]. As cercariae rely on glycogen to meet their energy requirements for definitive host infection [57], the preferential expression of glycogenin (providing more energy reserves) in male cercariae may offer another explanation underlying male-biased sex ratios in experimental and natural schistosome infections.

Flavohemoglobin, a nitric oxide (NO) scavenging protein, is widely expressed across many bacterial and fungal pathogens to defend against host reactive nitrogen intermediates [58],[59]. The differential expression of a flavohemoglobin-like molecule (CONTIG6697) in male cercariae (possibly acquired through lateral gene transfer [60]) may serve a similar function by dioxygenating NO [60] released from classically activated macrophages. As these cells can kill newly transformed schistosomula [61],[62] by a NO-dependent mechanism, neutralization of anti-parasitic NO by a flavohemoglobin-like molecule represents an effective strategy to increase male survivability during infection and skin penetration. Although flavohemoglobins have yet to be described in metazoan organisms, this potential schistosome ortholog is also highly expressed in 3-hr mechanically transformed schistosomula (but not 24-hr, 3-day or 6-day schistosomula, data not shown). This argues against bacterial/fungal contamination and suggests that schistosome flavohemoglobin might be a key defence product needed early in the parasite's life cycle.

Differential transcription of cathepsin L isoforms in the cercariae (male-associated-CD140068, SMLC14B04 and CD136140; female-associated-SMLC17E04A) suggests that this life-stage has distinct cysteine protease-dependent mechanisms employed for definitive host invasion. This supports findings by Dalton *et al.* [63] demonstrating that the process of definitive host invasion involves multiple cathepsin L-cysteine proteases. However, our data now adds a gender-associated element to this process. Serine proteases are also involved in skin penetration [63] and our identification of a novel member (EOS serine protease, CD136179) preferentially transcribed in male cercariae adds information to the biology of definitive host invasion. Whether the EOS serine protease ortholog is related to those serine proteases previously characterized [64] is currently unknown.

One of the most striking findings uncovered in this study, however, is related to the female-associated expression of numerous transcripts related to repetitive and mobile genetic elements (Dataset S4 and S5). Mobile genetic elements (MGEs) are one of the most influential driving forces in shaping eukaryotic genome evolution [65] and numerous types exist within the genome of *S. mansoni* [66]. Interestingly, a recent study demonstrated that at least four MGEs are more highly expressed in the cercarial life-stage when compared to schistosomula, adults, eggs, miracidia and germ balls [67]. Our data suggest that this transcriptional pattern is restricted to females, although why is presently unclear. It has been demonstrated that the absence of homologous recombination between the sex chromosomes leads to the accumulation of transposable elements in the Y chromosome of *Drosophila* and mammals [68]. However, whether a similar accumulation of MGEs occurs in the schistosome W chromosome (easily identified by its high degree of transcriptionally silent heterochromatin [69]) is currently unknown. Even if this were the case, there is currently no explanation as to why this would lead to differential expression of these accumulating MGEs on the W (or any autosomal) chromosome. One tempting possibility is that endogenously transcribed small interfering RNAs (siRNAs) originating from female repetitive elements (especially the W1 and W2 sequences, Dataset S3 and Figure S1) within the genome help to maintain schistosome chromatin structure and genome integrity as well as participate in post-transcriptional gene silencing [70]. These detectable, repeat-associated small interfering RNAs (rasiRNAs) could efficiently protect the female schistosome germ line from the adverse effects of increased retrotransposable expression and genome integration as has recently been proposed for *Drosophila* [71]. That this mechanism may occur as early as the cercariae life-stage (lacking ovaries) is remarkable. These findings need to be observed in the adult female (containing ovaries) as well, but if true, rasiRNA-mediated epigenetic control of gene expression represents a novel mechanism to ensure female schistosomes harbour the regulatory capacity to protect future progeny from deleterious genomic insertion events. Studies to detect these MGEs in adult schistosomes, using the new DNA microarray, are currently underway.

### Conclusions

A standardized method for conducting two-channel schistosome DNA microarray experiments is described, which utilizes gDNA as a highly suitable and easily obtainable reference material during indirect hybridization strategies. Comparing indirect to direct DNA microarray hybridization strategies led to the identification of discrete as well as concordant sets of differentially expressed transcripts in the morphologically identical, but chromosomally distinct cercarial life-stage. Further functional interrogation of these transcripts will generate a more complete picture of factors and processes underlying the schistosome dioecious state.

## Supporting Information

Dataset S1 Provides details regarding the real time PCR experiments including primer sequences, Tm of each primer, annealing temperature used for PCR and product size for each amplicon.
(0.01 MB XLS)

Dataset S2 Worksheets provide details of the differentially expressed transcripts identified in this study. Column A indicates unique identifier for each 50-mer sequence on DNA microarray (sequence available at SchistoGeneDB [22]). Columns B-P represent best BLASTx matches of parent sequence (including names and E values). Where cell is empty, no hit was found upon BLASTx analysis. Columns Q-S (where present) represent Gene Ontology classification categories as determined by Goblet [31]. Worksheet 156 female dual provides details of the differentially expressed female transcripts identified by both DNA microarray hybridization methods (156 transcripts, Figure 3). Worksheet 244 female direct provides details of the differentially expressed female transcripts identified by direct comparison of female cDNA to male cDNA (244 transcripts, Figure 3). Worksheet 192 male direct provides details of differentially expressed male transcripts identified by direct comparison of male cDNA to female cDNA (192 transcripts, Figure 3). Worksheet 188 male dual provides details of differentially expressed male transcripts identified by both DNA microarray hybridization methods (188 transcripts, Figure 3). Worksheet 2304 all indirect provides details of the differentially expressed male and female transcripts identified by indirect comparison of cDNA to the common gDNA reference (2304 transcripts, Figure 3). Transcripts coloured blue represent the male transcripts (1277 transcripts, Figure 3) and transcripts coloured yellow represent the female transcripts (1027 transcripts, Figure 3).
(1.09 MB XLS)

Dataset S3 RE (repetitive element) RT-PCR primer information. W1 and W2 repetitive element primer sequences, Tm of each primer, annealing temperature used for PCR and product size for each amplicon.
(0.01 MB XLS)

Dataset S4 Mobile Genetic Element (MGE) Oligo information. Worksheet provides details of the differentially expressed MGEs identified in this study. Column A indicates unique identifier for each 50-mer sequence on DNA microarray. Column B provides the corresponding 50-mer sequences deposited on the DNA micoarray. Columns C-Q represent best BLASTx matches of parent sequence (including names and E values).
(0.08 MB XLS)

Dataset S5 MGE sequence information. FASTA description of each differentially expressed MGE sequence found in female cercariae. 50-mer oligonucleotide sequences of each of these MGE are included in Additional file 5.
(0.13 MB DOC)

Figure S1 RT-PCR confirmation of W1/W2 from mixed-sex cercariae cDNA. Mixed-sex cercaria RNA was used in a reverse transcription reaction as described previously [20]. -RT indicates PCR performed from cDNA samples prepared in the absence of reverse transcriptase, +RT indicates PCR performed from cDNA samples prepared in the presence of reverse transcriptase, gDNA indicates PCR performed from mixed-sex cercariae gDNA. PCR utilized the following cycling parameters: 95°C for 3 min, 94°C for 1 min, 59°C for 1 min, 72°C for 1 min. Steps 2–4 were repeated 39 times (total of 40 cycles). PCR amplicons were electrophoresed on a 2.0% agarose gel. W1 and W2 amplicons were subcloned into TOPO 4.0 (Invitrogen). Representative clones were sequenced at the IBERS gene sequencing service unit (ABI3130xl sequencer and BigDye chemistry). W1 and W2 expressed sequences were deposited in GenBank (W1, still awaiting and W2, EU980106).
(7.00 MB TIF)
