# Supplementary material for: Use of Genomic DNA as an Indirect Reference for Identifying Gender-Associated Transcripts in Morphologically Identical, but Chromosomally Distinct, Schistosoma mansoni Cercariae
Source: PLoS Negl Trop Dis. 2008 Oct 22;2(10):e323. doi: 10.1371/journal.pntd.0000323 (PMC2565838; doi:10.1371/journal.pntd.0000323)
Supplement: Dataset S5 — MGE sequence information. FASTA description of each differentially expressed MGE sequence found in female cercariae. 50-mer oligonucleotide sequences of each of these MGE are included in Additional file 5. (0.13 MB DOC) [file pntd.0000323.s005.doc]

>gi|623342|emb|Z47549.1|SMLIBREPF S.mansoni Liberian strain repetitive DNA

CAACACAGTGAAATTCTTCCTTCACACATATCTACCATCCAATGTCTTCGCAATATTTTGGAGTGAAATT

TGCTTTTCTCATTATATTGTGCATGATGACTGATGTGACAGGAATGAGGATTATGTTGATATCGTCTGAG

TCAATGAGAATTGTGAATCGGATGTGCAGATGAGAGGTTGTGCATACTTGTTCCTTGTGACACAAAGGAG

TGGTGATGCCAGTTCGAGTGTTTGTGGATGCGATGGTGTTCACACGTGGATTGAATAAGGGATGAACAAA

TGCGATGATGCAAATGAGTGTGTGGTTGTGCTGGACCAATGTGCATAATGGAATCGTTGCTTGTGCACAT

GGACCACCACAAATAACACACTCAATTCATACTCCGTCCATTTAAACATGCATTGCGTTTTCATCACCAT

CACAGTTCGCATTATCATTTCGAACATTGAGTTGAATGTCGAGTGGTGAATTC

>gi|1885342|emb|Y11480.1|SMW2REPM S.mansoni male W2 repetitive sequence, clone pUC18-37

TTGCTGATGTGCAGTTTGCCGATGTCTATTCAAGTGATCAATCAGTATGAGTAGAAGACAATCATGCACA

ACGATTGAACAGTCAACAGCTCAAATGATGAGCCGAGTGCCGAGAATCGTTGCGCATTTCCTCAATCAAT

AAACAGTGAAGTGTGTTCACGAACGATTCCCGTTCGGTTGAGATCGGGAAACGCTGAAATTGTGTGTTAT

GCTCTTTCGGACATCAGTTTCCACGTGACATTGATGAAGTGAGACTGTGTTGAGGTCGGTCGGGTTGAAG

GAAATCTAAGTGTCGGTGGAAATGCACACTGTGTGTGGTAATAGAACGACGAAAATTACGAATGCGTCTA

TTGAGCTATGTTCTGCATATTGGTCTGAACAAGTGATGATCGAAGGAGCTGTGATTGTGCATGTGTATGA

GTGCATAAGTCAACAAAGTTGATAACGAACAACCTGGTGAATTGACCGCATCTGTGTGATGTACAATTAG

ATGATGTAGATTGTGGAGAGGTTCTACTGGTGATTGGTTGTGACGTGTCGGAAGAACGTTGGGTGTCAGG

CCACGAAATGTGTGTAAGGCGAAACGCGTACGCCGTCAGAACGTTGTTGTGGTGGGCTGTTTTCGGACCT

GCATCATACTCGGAAGA

>gi|624965|emb|Z47548.1|SMLIBREPM S.mansoni Liberian strain repetive DNA

CAACACAGTGAAATTCTTCCTTCACACATATCTCTCATCGATTGTCTTCGGAATATTTTGGAGTGAAATT

TGCTTTTCTCATTATATTGTGCATGATGACTGATGTGACAGGAATGAGGATTATGTGGATATCGTCTGAG

TCAATGAGAATTGTGAATCGGATGTGCAGATGAGAGGTTGTGCATACTTGTTCCTTGTGACACAAAGGAG

TGGTGATGCCAGTTCGAGTGTTTGTGGATGCGATGGTGTTCACACGTGGATTGAATAAGGGATGAACAAA

TGCGATGATGCAAATGAGTGTATGGTTGTGCTGGACCAATGTGCATAATGGAATCGTTGCTTGTGCACAT

GGACCACCACAAATAACACACTCAATTCATACCCCGTCCATTTAACCATGCATTGCGTTCTCATCAACAC

CACAGTTTGCATTATCATTTCGAACATTGAGTTGAATGTCGAGTGGTGAATTC

>CONTIG11025

ggcgactaaatatgcccacatcgtcggtgcactacctatagatgtagcaacggaagtcagtgacctgatagataacgtaccagaaacggacccctatgacaaaataaaagctgcagtgatccaccgtacttcgcagtctgatgaaaaacgcttgcagcaactactcaccgcatgcgagttaggggacaaaagaccttcacagttgcttagacatatgagacaactatcaggcccttacaagttagacgaagcattacttaagcagatatggttacaaaggttaccatataacgtcagacaaatcctcagcatatcaggagcctctgtcagtcttgatgatttagcagacatggctgataagatgatagaaatatatcctgatagtcacggcgttagcgcgatacaatcctcaaatgcagaaaacatgagcgacgcaatcaacatccaacaacaaataacactgttaacacagcagctagcaacactgcaggcgactgtttctaccatccattctcgacctcccagatctacgtccagaaggagatcagtatccagacatcgtcttaggtcacctaagcgggcagccggaatttgttggtatcattcgaattatggtgagaaagcacgttgttgcacaaaaccgtgcaactttaagacaaacaacccaatctatcagggaaacgaagtggccagacagtaacggcggcagctgctactggccaacatgttagccgcctatttcacgtcagggatcgcatttctggctcggactttttagtcgatactggagcagaaatcagcatcatcccactccatctctcccgcagacaacagacaacaagcactaaactgtccctaatagcggctaacgaatcagttatcaaaacttacggagaacaatctcttatattagacctcgggctccgca

>CONTIG10772

tgaacgttattctgtaaggttatttccagtgccagagcgagttattgataattatctacaactagagaaagttagataaaagcaaagagcacggaacaacaaaacaatgattgccaatacatgtctgtcaggttatcactgttcatgttagatattattcaaatgtgttagagttgatagtgacagtattaatgtaaagtgacagcaaaaaaatggttaaacatctaaagttcctataatctcacttttggagtaagatacacttacagacgcaagagcatttattgcaatttcagaggagcagtatccatgaaaggagcgaacaaataatgagagagattttatctaacggatagtttgtggtagattattgaggaaaccagaaaatttttcaatgaagtaattcatatagagagaagatctggagtatgtttgtttcgctgaagacaaggagttacgaatgtcgtaatgtgaagcttcagcatattgagtcgcctgcctgtttgtgaagaaaagtttgttaagtataatgagagagattttatctaacggatagtttgtggtagattattaaagaaaccagaaaatttttcaataaagcaattcatatagaaagaagatgtagaatatgttattttcgctacaaacaatcagtagacacttcgttcaaacgtcaaaggag

>CONTIG5844

aagcaaaggtaaacagttgcttagaattatcagttataactaaacaaccattaattaagccaaacttttgtgcacatctatagcaaactacagaataaataagacaagagacctatattttagaccacaaatttaatttccataagtatccattagactcggcttataaggagcacatactgtaaatgacctttgtggtttgtaggttgaataatctattattattattattattaatggctttattcagtattataatctggtacaatatagaattctcagcacgagttatttcaacaagttttttaccaaaaaaaacaaaaaaaaccaaaaaaaaaacacagaagaggaatgaaaaaggtttaagaaaaactgagtttatataaattatcaaatttgaaacatgcttaagaagacaggttatttactaggtcaactctaacagcctgttcgtcacaaagtaaatttgctaagtaaggtattacagaacttttttagacttgcttgcgtgtatggattttaatgtatttacgtcttatttattttatttatttaaacacataaatattggtacaaggaagcaccagatacatatgcgccatacaaatctcatccgatttgcttgagggctgtgatactgcccaggtgcccgaactgaagcaggtggttttcttagagggccacacccagagcctttgacctaaaggtctaaccaacaaggcagtggagcatcgtaagaagatgcagtcccatggtagccggtgacaaacaatcgactcatacgccatttgttccctcaggacactacagcccatgtgcaccattgatttggaatcagatttttccaactcgcagtgtccaccaacccggttaaagtgccggacatccgcttttcgtcctctcaatttcgtaaacaacagcagtgcaacgagaaggcagtgagtaggacttcctggcagaggctacatacgcgtgaccatgtc

>SMLA27E06

gccagtgtgctggaaagattcaaaatcctttaatatcaaataaagtcattataactcactcaagcttgctacccctctttggccagttttgtcttgggcttccctttccagttattccattgggtcactcataatcctaatttctacctcccattatttgctccatatattctttagtttatctcttacctcttgaactcgactcgtgatggaattcagtggttttactaaaatgtgctctatctacatccatcacttttacttgatttcatccacaattggttgttagtttgttctttaccagaaagggttattgctaatggtctctcacgaatagatctttggtatcttgcataggcatttgatgataaatgcctgcacctcttttggtagtgatgttagcattgggactgacagtcatgtagttcctctgtagttcctatatttactgagatctatttctttcagtatcttcatcaggtatccttttttcaatcagatggtacttattcttcttcacaaatctttctaaacaaaacttaggacctccttcaagatacctctatatctaactgcaatttttcagctagcatgttgtctgatccatattttaatattctttcagttttttgactgtggtttccaggtctttacattttcgcatgttatctatagtgcacctcttcgcttgctcgttcgcttctgaactttcggcctatgtcttgaatttatccgctcttgttcggttgttgttaactattatcttctagcttttcctttcttgaatcttgcctacgggtatcaatagggacctgttctttatcaaggtgcatcttacgaccgagagctttctatcatgttgattttattacagctttaattcttttccnagtatcgncactgtggttnccccttttctactacatcttgtgtgggatggagttactnacgagagtatcttgaattcgctcagttatagcattttgaagaggatgtatcaaactttataaagtgttttgtcagntgtccatcacttctaatcttcattttaacctgccactgtgcggaagttatttgagctaattcactctattccttttacgtttctgagacctgntatgccggat

>CD060968

gagacagacattagaagaatgaataagagttggatatagctagaaaggaaggcctaggacagagtggattgttgaatgctggtcggcggcccatgttccatcgggagtaacttacgtaagtaagtaaaaaattgatactgctgaaactcattttcatcgactgtacgatcattcatcgagtaggaaatttttttctttacagtaacttgaatcagagcattgtcgactgttattttacactttatatgatacagacattaatatttactttacttttgtttgtatttatagaatcaccaagtaagtcaacttccatagatatatatcattgatttattattactcttaattacttacaaacagtaggtaacataataagcaaagatggatagtggctagcagtggaatccaggaggagtgtttcgtcttatttcgtactcgtcatctagatgtgtctgaacctcagagttgatgctcactccgggactcgaacgcagtactgttggcttcaaacgccatcacgttatccacttagttactgagt

>CD114188

tgttgtcaggtcctactgctttcctactcttgatttgtctgatgaccatgttgatttcgccgatcgtttgtgggatgacaactatgggaagagctgtgtgtgttgcttcgatatcttgtatgtttagtgtgactgttctattcaagatttttctaaagtgttctacccgcctattcctctgttcttgaatataagtgattggtttttctttctttgtccttgaccgttctctccggttcactatccttgcctggtagtttcttcgttgtgtcatatagtcatctcatatttccttctcttgaacctttttccgctgtcgttgctaggtcttccacgtttttccgcctttagattccaatgctcctccttacccatttgctgtgtattctattcgtgccttggctttcactattcttgttcggctattgtcaattgctgtcttgttcttaacttcttgagtcttgttcaggatttctatagagatccatcttatacgtcaatgcttcttgcggcccataacctcctgg

>CD192752

cccacaaaatcttcagcattttaaatccacattactggactgtagtcatggatatgttaatatacaatgtggtaagaacaacttattaactaagaaccatctggatcaactaatgctcctgagaagaaataaggatttaatactaagtaaacctgacaaaggagctggtgtagtcttacttgaccgccaagaatatttggaaaaatgagattaatcttaaac

>CF500709

gtgttaccacttagctatacattggctaaaagttgatcgatcgtctgctggtcagagagaaatgtaacacgtatgacctctatttttgggcaatcatatggtgagtcaggatccggtttaaaaatgacgtcaggcacttgatcaataacgtcatccgacagcaacgaatttgcgtaactaaatttcgtgatctggcttgtgattttgtttgtaacaaaaaagctttctaagcgcgcaaaccagagttctgcctttcgaggattaaaaacaggtaaaggtacagatatggcacccacggaaatggacttagatgaaaggcttgttgaattattgagtgacatgatacaaagaaataataacagtagcaataataataagctgtgaactaagacatacaatatgaagaaataacaaataacaacaagaaatatatgaaagactcaccgaataaaaaaactgttactttaggtacaaatatgcgaatatatcaatataatcccaaaggaacaaagttatgagtaaatagattatgactttcaatgaatttttaataattgtaacagtaatttcaat

>CONTIG12200

cnnnnnncgggtgnnnnnnnnnnnnnnnnnnnnnnnnnnnnnnnnngnnnnnnaagggtcgtagagtcaatctctctgtcgcgtgttagatagatagccttgcttgtcacgtctcggcttgtgtctgtgagactgncatggtaactttgattgcagtttcagttaagtacagttccatcgactgatgaggaacattccagttcaatactgtaacgtcatcatcataagtcatctacatatgatcactgtctctgataaaacaaaatgatggaatgtaaagattttaaaaagatcatagaccatgttanatgagaggacaagaatatacatctgaagatataaagtatatatgacaaatagntcgaaggaattactttccgagataatacaatgtaaagtttctattctatacaaagtaataaggttccagaaagaaggtattcatatttgaaggtgtacagtctaattacattgtcttgcagatataactcagccagctacaatgtaggacccgagacatatatgcatcggtccaagttaccatatcacagatataactcatgctggttaagcattcactggccggatactatcagcaacagcgttttatgggagaagacaaatcagcttccagttgaagaggaaattaagaaaagacgatggaagtggataggacatacattatggaaatcaccaaactgcatcacgaggcaatccctaacttgaaatggtgaagggaagcgaaaagaggaaggtcaaagaacacattacgccgggagattgaagcggatatgaaaaggatgaataaaaactggaaagtactggaaaggaatgcccaggacagagttggatggagaatgctggtgagcggcctatgctcctcgacgaggggtaacaggcgtaagtaagtaataattgttgttagtgatatcaggtacttggaaataaccaacaggaaaacttgaacctaaatttcgttctattcggtaccggtcagaaaggtgggcctttaagatggagggaaccggtgctccttggtcgattcgatcgcacgttacccagatacatagtctaagatgttacgaccaagttattcgggctgcgagtgatcttctgcgggacaggaatacaattgattaaccactgagtaagtggccaagtccacgtttatttattcctttagtg

>CONTIG3811

ttaggagtgccccctacccacctgtttgtctgatcttgaatttcagtcatttacttgcctactttgtacttgaccggtctctctggtttactatatttccctgtatcatatagttgtttcatcttttcttctcttgcaacattatccactgaggttactaggtcttccccgtatttctgtttgtcggctataattttccttctcacttgtttgtttgcttctgtgtatttagcctgtgccttgacttcctctgctcttgttcgactgatgttaattgctgtcttgtttttcttcttccttcattgaatcttgttcgaggtttcgattgagatgcattccttgtgataattcttcttgcggttgattatctccttgtacgacaaaggtagtg

>CONTIG7540

agggatcttttttgttaacttacaagggaaaaagaatgataagtctgaaaatcgcaaaataaacgggacactttcagttaacttacaagagaaaaacagtggtaagtgaaactaatgatcaatttcattccagtcaagataaaacaaacgttttcgatgcaaagacttcaggagaaaggctaaaagtaaacatgcgatgacagtcacacatcatattttcataccgactgagatcagatgttgttatattccggttttaactgcagtcaaatacatatctacgactgttacccctcgtcgagtggcataggccgcataccggcattctccatttaatcctgtcctggacaaccccttgaagtttcgctcagttgtttttcttccatttcatatctgcttccataacatggcataatttgttctttggctttcttttctacacttctcttccggattccaagttaaagcttgcctcgtgatgcagtttggtaattttgttaatgtatgtccgacccactctcaacgtgttttcctgaattgcccttcagctgaaagctgattcgtcttctcgttgctaaatcttccacatatttacgtttgtcggttctgatgttcctcttcacttgcttgtttgcctctgtatattcggcttgtgcc

>CD168619 (>CL1422)

ctgggagtagaaggaataattatgacgtttcatgatgaaagccgaattccttcggaagtcatgaggccgatgcactttcttacaactagagcgacaatttttataggtacatggaatatccggacaatgtgggagaccggaagagtcttccaaattgctgcaaagacataaacttgtatgtagaactagtaaaatgcaacaagtctgtgataagaactaaaggcgagactagaatttattgaggaactaatcaggtataagataagaggctgatatatgaagagattacaatattcactgttcagtaggagtagtcaggttgtgacaggtttcatgaaagtcaggttgagtaagagtaacagacgttcggtgatggatataattgaaaatcaaccgacatcaagatcacaattttcaatacaaatgtcaacacagttctactgtatggggtggaaacctgaagaattacagaagccatcattaagaagatacaagtgtatattaatagttgtn

>gi|833801|gb|U10109.1|SMU10109 Schistosoma mansoni female-specific repetitive DNA, repeat W2

AAGCTTGCTGATGTGCAGTTTGCCGATGTCTATTCAAGTGATCAATCAGTATGAGTAGAAGACAATCATG

CACAACGATTGAACAGTCAACAGCTCAAATGATGAGCCGAGTGCCGAGAATCGTTGCGCATTTCCTCAAT

CAATAAACAGTGAAGTGTGTTCACGAACGATTCCCGTTCGGTTGAGATCGGGAAACGCTGAAATTGTGTG

TTATGCTCTTTCGGACATCAGTTTCCACGTGACATTGATGAAGTGAGACTGTGTTGAGGTCGGTCGGGTT

GAAGGAAATCTAAGTGTCGGTGGAAATGCACACTGTGTGTGGTAATAGAACGACGAAAATTACGAATGCG

TCTATTGAGCTATGTTCTGCATATTGGTCTGAACAAGTGATGATCGAAGGACCTGTGATTGTGCATGTGT

ATGAGTGCATAAGTCAACAAAGTTGATAACGAACAACCTGGTGAATTGACCGCATCTGTGTGATGTACAA

TTAGATGATGTAGATTGTGGAGAGGTTCTACTGGTGATTGGTTGTGACGTGTCGGAAGAACGTTGGGTGT

CAGCCCACGAAATGTGTGTAAGGCGAAACGCGTACGCCGTCAGAACGTTGTTGTGGTGGCCTGTTTTCGG

ACCTGCATCATACTCGGAAGATAGCAAATCAGTTGTTCATCGTAGTAGAGGAGTACACACGTTGGACGAC

CAAGTGCGTAAGCTT

>gi|1885341|emb|Y11481.1|SMW2REPF S.mansoni female W2 repetitive sequence, clone pUC18-116B

TTGCTGATGTGCAGTTTGCCGATGTCTATTCAAGTGATCAATCAGTATGAGTTGAAGACAATCATGCACA

ACGATTGAACAGTCAACAGCTCAAATGATGAGCCGAGTGCCGAGAATCGTTGCGCATTTCCTCAATCAAT

AAACAGTGAAGTGTGTTCACGAACGATTCCCGTTCCGGTTGAGATCGGGAAACGCTGAAATTGTGTGTTA

TGCTCTTTCGGACATCAGTTTCCACGTGACATTGATGAAGTGAGACTGTGTTGAGGTCGGTCGGGTTGAA

GGAAATCTAAGTGTCGGTGGAAATGCACACTGTGTGTGGTAATAGAACGACGAAAATTACGAATGCGTCT

ATTGAGCTATGTTCTGCATATTGGTCTGAACAAGTGATGATCGAAGGAGCTGTGATTGTGCATGTGTATG

AGTGCATAAGTCAACAAAGTTGATAACGAACAACCTGGTGAATTGACCGCATCTGTGTGATGTGCAATTA

GATGATGTAGATTGTGGAGAGGTTCTACTGGTGATTGGTTGTGACGTGTCGGAAGAACGTTGGGTGTCAG

GCCACGAAATGTGTGTAAGGCGAAACGCGTACGCCGTCAGAACGTTGTTGTGGTGGGCTGTTTTCGGACC

TGCATCATACTCGGAAGA

>gi|160956|gb|J04665.1|SCMDNAP S.mansoni female-specific DNA repeat W1

GAATTCGTTCAACACAGTGAAATTCTTCCTTCACACATATCTACCATCCAATGTCTTCGCAATATTTTGG

AGTGAAATTTGCTTTTCTCATTATATTGTGCATGATGACTGATGTGACAGGAATGAGGATTATGTTGATA

TCGTCTGAGTCAATGAGAATTGTGAATCGGATGTGCAGATGAGAGGTTGTGCATACTTGTTCCTTGTGAC

ACAAAGGAGTGGTGATGCCAGTTCGAGTGTTTGTGGATGCGATGGTGTTCACACGTGGATTGAATAAGCG

ATGAACAAATGCGATGATGCATTAGGGTGTGTGGTTGTGCTGGACCAATGTGCATAATGGAATCGTTGCT

TGTGCACATGGACCACCACAAATAACACACTCAATTCATACTCCGTCCATTTAACCATGCATTGCTTTCT

CATCAACACCACAGTTTGCATTATCATTTCGAACATTGAGTTGAATGTCGAGTGGTGAATTC

>gi|833802|gb|U12442.1|SMU12442 Schistosoma mansoni OZ.A female-specific retroposon SMAlphafem-1 repeat region

AAGCTTTATCGAGGCAATACGCACACTATGCACATATGTCAATAACACACTGATCAATTGCAGTGCTCAA

CATCAGTGAGAACATGCATGTAAACAATGCTGATTGAATTTGAACATGACCAGTTTGCACAAGTGAGTGG

CTGTGGGAAATCAGTAGGTGAGTGGATAACTTGATGGCGTTTCAGGTGAATGTTACTGTGTTCCAGTGTC

ACAGTGAACATGAACACGGGATGCAGGTACATCCAGATGGTCAGTGGCAAATCGAACGAAACACGCATCC

TGCATTCCACTGAAAACCACTATCCATCTCCCCATTAAATGCTTGTCAATTGAAGCTT

>CF493654

tagtattgtcagcgaaggtcaactcgccttcgaggtgccgatggtttgtcgtggactgtgagatatgttcaaaatgtctgcactctcatagtgagtagccgtcggattttgtatgcagagcacacgtcaaaggtttattgccattgaaaaaggatgaaattttgaccttctactgcgggcatgaaatgtttgacggcacagtaggatgctagccaatctctactgaaagagctataactcgtctccgagagggtgaggcatcgtgagaaaaattcgagaggt

>Contig5532 EST cluster from Schistosoma mansoni 28/06/2002, 172bp

ggacggggggtcacaattccaatcgcacctcttcaacgaatttacgcgca

tcttgcgaatccatcacatcactacaactgcataccatccagcagcaaat

ggactagtggaacgattccacagacaacttaaaagctcattgatggtaca

gcctgatgtaactagatggagt

>gi|2636684|gb|U66334.1|SMU66334 Schistosoma mansoni SR1 non-LTR retrotransposon, internal fragment E3, pol pseudogene

GAATTCCTAGTTGACGACCTCTCAATCCTGGCTTCCCTGGGGAAAAGTGATCACGCCGTCTTATCATTCA

GCTTTGTCAGCAAAACGGAGCTACGATATCCTACTAGCAACAAGCGCTGGAACTTCAAACGGTTGAATGT

GTTAGCTTTACAGGACTATCTACAACAGGTGGATTGGGATGTTCACCCTCAACTTGAAGTGGATGCTCAT

TTGGATTTTTTACTGCACACGATCTTATGTGCTACTGAGCATTCAGTTCCTAAAATGGTCCCAAAAAGCT

ACAAGCAACCTCCAATCATCAAGAACCGCACTCGTCGTTTGCTAAGCCGCAAAAGGCACTGTTGGGCTGA

ATATAAACGAACCGATAACAACGGCGCGTACAGGCAATACAAACATATAAGGAACATATGGAAAAAGGCA

ATAAGAGAAGACAGGCTTCAGTTCCAGACCAAGCTTATCGATAAATTCGTCTCCAATCCGAAAAGCTTAT

TCAGTTACGCAGCTTCTCTTCGACAAGGCAAAACTGGAGTTTCCCAACTGCTTGGTCCCAAAGGCCCGAC

CAATAACGACAGTGATGCCGCCAACCTTTTGGCTGAACAATACTCCCAGACATTCTAGCTGACCCACATC

AACTACACTGACGAAAGCTTCACCTGCACCTGTACAGGACTTTCCGAAGTGGATCTGAGTGCTGACCTGG

TGCTCCGTAAACTGTAGCACCTAAGAAAAGACACTTCTCCTGGTCCAGATATGGTTCATTCCGCTGTTTT

GAGGGAAGCAGCTTCAATCCTGGCGACACCACTTAGCGTGATGTTTGCACACTCGCTAAGCAGAGGCAAA

CTACCGGAAATTTGGAAGCTGGCCCACATCACACCAATTTTCAAAGGAGGTCGACGCAGTGAACCCTCAA

GCTACCGACCAGTGGCCCTTCTCTCCATACCTTCCAAAATTATGGAATCCCTAATATACGACGGTATATT

ATAATACTTATCATCCTCAAAGTTCTTCTCACCTCAACAGCATGGTTTCAGAAAAGGTCATTCTTGTATG

ACCAACCTGCTGACTGCGGTGGATAGATGGACAACCATCCTTGATCGCAAGGGGAAGGTTGACATCATCT

ACCTGGACTTCTCAAAAGCTTTTGATAGGGTCAACCATATGTCTTATCAAGAAGCCTAGACGATTGGGTA

TAAAACCCCCTTTGATTGATTGGCTCTCTTCATATTTAGAAAACCGACACTTTAAGGTCAGGGTTAATTT

CACTCTCTCTCAGGCTATGGAATGTCCTAGTGGGGTCCCCCAGGGCTCAATACTAGGACCTCTTCTCTTC

TTGATTTACACTAACGATCTTCCTCGACAAGTTTTATCTGACTTATTGCTTTTTGCTGATGATGTGAAAC

TTTGGAGAGAGATACGTAATCATAATGATATACTAGTTCTTCAGGAGGATCTGACTCGACTTCAAAGTTG

GGCAGACGACAACGGACTTACCTTCAACACTTCAAAGTGCAAAGTAGTCCATCTGAGACATGTTGCAGAC

CATAGTTATAACTTAGGTAACTCCCCTCTAGAAATTTCCCAAGTCGAAAAAGATTTAGGAGTGTTGGTAC

CCTATGACCTGAAATCGTATGCGAACTGTGACAAAAATGCCTTTCGAGCAAACTTTGCACTGGCAACATT

GAAGCGCATTTTTGGCCAGTTTGACAGTAGAACCTTCCACATAATCTTCAACAGTTTTATTCGTCCCCAT

TTAGAGTATGGAAACATAGTATTTCCTCCCTCCCTCCAAAAGGATAAGGACACTCTGGAACGTATACAAC

GTCGAGCCACGAAATCAGTTCGGGGACTCAAATTCAAACCTTACGAAGAGCGCCTCCAATCACTTAACCT

TTACCTGTTAGAGTACAGGCGTCTTAGGGGTGACCTTCTTATGACTTACAGTATACTTAACACTTCTGGT

CATCCCCTTAAACATCTTCTTAAGCTTAGTCATAACACTAACCTCAGAGGTAACACCCAGAAATTGGAGA

CCCTATATAGCAGAAGAGACTGCAGACACAACTTCTACTCCGTTAGAGTTGTCAAGTGCTGGAATTC

>CD066571

cgactccatgtgagggcttgactttcgacgacgttgtgtgatttcctcagagtttgtcctatacacttctagcgcttcttcttgatttcttcctcaactgaaatctggtttgttgtctcgcacagtagaatgttgctgatagtatctgaccaacaaatccgaagtatctttcgtacacaactgttaataaacacctgcctatacttattaataaaaaataaggtatttaattgttctcttcatttctacattgataaacataaaatggatcatttaaaaggatcagccaaaactttgtttaaactatgaggaaaatataacagccaaataaatgatgcctacgtaaaaatgaagattatgaattaagtatggttattgtttacatcttacaaaatatatgcttgaacgtgaataactaaagacaaataaagagatgttaaattatagaacgagttctattacactatataccttttaataaatattcct

>CD089057

aggcacacaagttgttagacttatccttcctccacaaacattgaaaattcttggattatgtaacaattgtagtagtaataaggcggttgtactttacgttcaagagtcacaaacgccccacaatgatgaaaattccccaatattttatcctgataagtttatcaatctgttgtgcaatagcagtgattattgtggaattgatcttcctggttattattgtcacttaattacttgccaacttattcctcttacgctccatggagtggcgtatgtcgtttatcagtcttgacctgcacgtgttggtagtaaatcagagacccatcaatgacaaggaaggaaagacaaccatggagattgaagaagagaggaaaagatgggcagaatatttcggggaactgctgaatataacaaatccattgattccaccggacatcgaagcagcacacacacatcttcctttag

>CD167034

gtttccattcatcattctcatatctgcttcgaattgtccatgtagtgtgttattcatccttcctcttttctatttaccttcaggattttaggttagcacttgcctcgtgatacagtttaatgatttcctcaatgtatgtccgatccacttccaacgtcttttcctaatttcctcttcagatggaagatggtttgtacatgtttgatgatgtttgtagtggttctcaaagtttcagctccatacagtggaactaactgccttgaaattcgtattgaagattgtaactttgttgttgtctgacagacaattattttgagttccgtatgttgctcaactgtagtaatgcagtcgttgctttgctcatcctttcgtctacttttgcatccgatcttccttgtttatcgatgatgctgagcagtcaggtacgtgaaagattccacatcttccagagattccgtatcacgtgcgattagg

>CD167979

aggccagagaacactttacgccgggaaatagaagcagatatgaaaaggatgactgttaactggaaagaactgggaatgattgcccaggacagggttggatagagaatgctggtgtgcggcctatgctccttcacgaggggtaacaggcgtaaataagtaagtaacaaacagattgaataatacaggttttggtttaaagataagactttagtttattgttagtgacctcgatggtatataataatataaatatcaaattttatacgttttggggtgtaatgaaataaacttaaaatgtgaggtattgcagttcagtaagatgattgttttgttttcattcttattgcatgtacaatttaataatttgtctctttattaattgctaaattattcctttatgcaacatggtagcattgactaagcgctatttatagaggtaaaatccatgacagttac

>CF498163

atatatctatgcgtatacctctgtcaaacctctaacaacctgatcaataaatcccttaaaggtttgagctgcattagttagaccaaatggcattcgtaaaaactcaaataaaccaaatggagtgattaccgccgttttccgtacatcttctggggccactggaatgtggtagtacgcccgaacaagatcaattttcgaaaaaatcgtagcaccttccatctcggtagtgatgtcatgaatatgtcgcactgggtatctatctgctatcgtttgcttggtaacggctctattatct

>CF499068

tagccactaagtgcctgaactgtttaagacttagagctatacctaccaagccgaaaccgcgagaaatttctttcgcatctccagatacacgcgaccgatttccatacaggctaagtcaagtcaagtggatgaccatgacagaatcttgtattcgtgtttcgaagaaacactatacaaagatgacacaagactctcgtaccttagttagtaatccctgtcgtccggtttggcacacgatca

>CF501265

tcaaggcctgtagagaaatctgcacctttgttgtgttttgtgccatttgccttcttgataaccacagttagtgctgtccaagcagattcagaaacaggtgtaataactccttggagttgtagacgattgatttattcatcaacttttgataatgtcacaatatggcactggtctcttcgggcggaaaacaggtttagcccctggcttctatcgaagggttgttttcatagaagagcactgatccaacacaagctggaagacggttgagaactgcgcaattaaattcgttgtatatgcttccggttcatggggttgtcttcccagatggcatacagtacttagcgggacgtcagctagctgtagctggtcgaaccaatctaaaccaagtaaatttaaatcggctgacgttatgttacataccccagtaaacgtcgattctcagaaagaaactttaaaatctaattgtcccaccacg

>CF503259

agatagtcgacgtcctcacaaaacagatagctcggctacaactttcgaatgcacaccactgcaggtgtagaggaaggtccagaagtcgtacacgtcgtagttcaagcaccatccgacataacatttgctggtaccataaaacgttcgataaacaagctaaaaagtgtattcctccctgtaattttgcaagcacacggaaaagggaacatgaaggccgacaattagtgacgactgcggtcaccggccacaactcacatgaaagtcgtttgttatatgtcacggacagaagaaccggaacacatttattggtcgacaccggagcaggtgttagtgtagtaccacctactgcactcgaaaaaaagacacctgaccccatattgaaact

>CF502958

taatgtagccaactacgtgccaagcctgaagaaacacatgaaagatctaaaaatagactacagcaacgacgagtattcatacctcaggaactacataatagcattcacgtattcatcggacgaaacaatgtacaatcacctccgcagccgaattatgaaggtccatttaaagtgaatgcgaagacaaataagaccgtcacagtaggaaaagctaagaggaccgatgtgattagcatagatcgaatgaaaccagctctcattgatagggattttcaatagacaagtacaaacacttcgaaggcgatcacaccgacgaaacacgagtctcaagaatcaacgacgctacaagatctgaccgcagagtacgatagccacaactctatg

>CONTIG10455

agtagttctccacgtttcaaatctgtacagtaggactgtctcgacgttcgtattgaagattctcacttcgaagttagttgacagttgttctgagttccatttgttcttcaattgtagaaatgctgctctcgctttgccaatcctcgccttcacatctgaatccgatcctccttgtttatcaatgatgcttcccaggtacgtgaatatttccatctcttccagagcttctccgtcaagtgtaattcgattggtgcatgccgtattgtatctgagagtcttgcttttccctttgtttatattgagacctactgctactgaggctactgttacactggtcgtcttcttctgcanttgtcgntgcgtgtgtga

>CONTIG10955

taagcggtatcatatagctggtaaccgctagtggtgtgataaggactgtccattattcccattttcattttactttgatatgtccataaggagggattttgcagacgcatttaaacctcattgcattatcgaatgtcaaaaagaatagattgcgtacatgtttcgtgaaagtgtaacgaccaattcttacgcaagtatacatccattaatgttagtgatcacattatcacattgctattggacattgaatccgatgttacgtcgatttctcacaaaacttggcacgaacttggtcgaccaagcattcatcctactacctattgcatagagaacgcatcaggcaccccaattaaagcttctggaattatccaatgtattgttaaatttaatgacctaataatcaaatctaaatgttatgttactgacgaatctttgaggctatagtgtagtgactggatcacacattttcgaagggtctgattacccatcttatttgtaatctcgactt

>CONTIG11158

ctctaagggactctcagatcctgctatgtgctggtaccatcaaacatttgggtacaaagctcgaaaatgcagatctccatgcaaatttaaacgatcgcaccgattctcagtaacgactacggaaatcaacgcagctcccaggagcagtcgtttattctatgttcaggacagagtaacaggcgcgcaatttttagtagacacgggagcagagataagcgtcgtccccccattgagcaacgaaaaacaaaatatcgatactgcgctaactcttatagcagcgaatagatccgacttaaagacatatggcaagcgaaaactaaccttgaatttcggccgtaatatttcacttcgctgggagtttgtgatcgctgatgtgtccgtacccataattggtatcgatttcttacagaattttgacctattagtagattctcgacgaaaccaactagtgaatggagaacgctcagtagtcataagaggagtgaccaccgatcatgtatctatgaatttagtagtgtcgaaagataagaacgagcaatataagtcacttctaaataagttcgccgatttgacgagggcgtcgtacgacaacagagatcttaaacatacagtacagcaccacatcgtcacgaaaggtccaccgactagagtaagagcgcgaaggttgaacccaaaaaggttaaacattgcgaaacgagaattcgaaaaactaatgaaacttggtatcataaggccatctaatagtccctgggcatctccgttacatatggttcccaaaaagaatggagaaatcagaccatgtggagattatagagcccttaacaagcanacgatagcagatagatacccagtgccac

>CONTIG8539

agtacccaatgagcttcggaacatcacaaccaatcactaagtaaaacttcgcctgaatctaggatatctaagggcacatcgtctaaatgcggccacttgcccaagctgttcattaatgaccttgtcggtttatgttctggtatctgcgacactatcagagctccttcaatgttaacatgctcggattcatctaaagagtatacttcgaaagatgctcttgtaacccctgtagccttgttaccgcctacggtttgtactaccgctgacgtctgatcctccttcaacccgagaaacttcaaacaatctgatctaatcaatgttacatcagatccattatctaaaagggcatagcctacaacttcagtattccccgatttcaaccgcacgggaatcgttcctaaacacacatgaccgtctaaggatttagtgaatccacaataattttccgcagtcgaatttttctccgagccattaattgtcggataactgtgcaatagtgggtgatgtttcttcgtacatttgtcgacggtacagcgcttgctcaatctacattcgttaactttatgacctcgtctaagacatgagaagcagatacccttgcttttaacttgagaccaacgattttccactttaagcgctaaaaaacgtgtgcatttatcgacggagtggttgccagaacatatactgcacttagtactcacagtcatagtatcacagtgctctgataacgcatgacaattcaccttagtgctacatcccttttttggacgctctgctaaccgtccaaatctgcttgtagccactcttgcgcgggcggctacaaacttggtaagctcggcaaaatttggttctctatcgtcctcggtcaacttgtctggagta

>SMLA37C12

gccagtgtgctggaaagaaccaagtcatatttgtacgcgtaatcgaatagtagtggtgatcatagtccgtccacgactcgacatccactacaactggttccaccgtgctttcggagccggtgcccgtcattgtcgagctccctgctcatacaaggcgggaaacttcccagccggcgagtaaattcggccgtactcgccggcacttcacctcaggttggccgtttattttatgtgcacgattatcgcaccaatgctaggtaccttgtggatacgggtgcccaagtttctgtcgtacctatcggtaacagtaagtctcaagccactatgcttcgactacgcgctgcgaatggctcagtcattcccacctatggtacacgacaacttacggtcaacctgagcaaccgacgacagtatctgtggacgttcatcattgccgatgttcccacagctatactcggtatcgatttcctacagcactatgaattgctagtcgattcacgtaggctgcagctaattgatacttcgtcgaacagcaactttatgggctctaaagcccacacaaacgcgtaccgaatcacaggtgtatttaattcgcgtgacgatttattccacgttttattccagaaattccccaaattaactaaacctctcgaggagactccatcggtgaccaatcgtgtggtacaccacatagtcacccgcggaccaccagtcacggcaagacctcgccgactggcaccggacaaattagctttcgctanacgtgagtttgacaatttactagctactggtattattcgtccttctcacagtccttgggcctcacctctccatatggttcgcanaaaggatggggttagttggagaccatgcggagactaccgagcgttaaacgcagctacacgtttcgatagctatcccatccccacatacatgacatcacggcatcactcaaaggcacgacaattttttcaagaatcgatctggtacgagcatatcatcagatcccagtcgctcttgaagatataaaaaaactgccctccgaactccttccgggtaatttaattccctccaaagcctttttattaacgaaatcttctcaacttttccaaaggttttttaataacttttttaaaaaccaaaatttttttcccccctttttattaaccccctcttcctcctccaccgcaaataaaattttttaaccctatgtttatttttccacccttttt

>CD095971

ctgtcttgacgttcgtattaaagattctgaccttgaaattggttgagagttgtttttagttccatatgttcctcaattgcctcacacttcattgttccttcatttccacgacaatcattcattattctcgttctctttacttcgatcttcttaatcttctgatgtcaggtatttctctttcgaatgatgatacatactacttatatctatcgtcattagtatatatgcatgtatatgttaaccttagatttggagtaactcctacctaaaaaacagacattatatacaaatatttatagcaaatatgtatatactaaagaaaatcaaagagtttcattaaccggataactgtcaacatcagtattgttattattatttcatgaacacgataaacaattaagtgtttataagcaaagatgaatagtggctagcagtaaaatccaggacgcacgtttcgtcctatttaggactcatcaactggatgtgcctgcatctcagaactcatatttactctgagatgcgtacccagtacttttcacttcanacgccattacatta

>CF499598

tcagttttatgagaggctgcaatcaatcataatgaagtacccaataaacgacctaacacttctgatgaacgatctaaacgccaaagtcggaatggaaaacaacggttatgaagatatcatatggtgacatggactaggagaaaataatgaaaatggggagagcttcgcagatctatgtgcattaaacaaactggttataggtgctacaatatttccacacaaacgcatacacaaagctgcatcgatctcaccggatcgcactgcacagaaccagatagaacatatttgcatcactaaaaaattcacaaggtcagtggaagacttgagaaccttaagaggagctgacatagtttcagatcatcacctcgtggttgccaagatgaaactgaagctaaaggggcattggaaaactggacaaacagcattacgaatgatcaatacat

>gi|2636686|gb|U66336.1|SMU66336 Schistosoma mansoni SR1 non-LTR retrotransposon, internal fragment BS6, pol pseudogene

AAGCTTGGACGATTGGGTATAAAACCCCCTTTGATTGATTGGCTCTCTTCATATTTAGAAAACCGACACT

TTAAGGTCAGGGTTAACTTCACTCTCTCTCAGGCTATGGAATGTCCTAGTGGGGTACCCCAGGGCTCAAT

ACTAGGACCTCTTCTCTTCTTGATTTATATTAACGATCTTCCTCAACAAGTTTCATCTGACGTATTGCTT

TTTGCTGATGATGTGAAACTTTGGAGAGAGATACGTAATCACAATGATATACTAGTCCTTCAGGAGGATC

TGACCCGACTTCAAAGTTGGGCAGACGACAACAGACTTACCTTCAACACTTCAAAGTTCAAAGTAGTCCA

TCTCCGACATGTTGCAGACTATAGTTATAACTTAGGTAACTCCCCTCTAGAAGTTTCTCAAGTCGAAAAA

GATTTAGGGAGTGTTGGTACCCTATGACCTGAAATCGTATGCGAACTGTGACAAAAACGCCTTTCAAGCA

AACCTTTCACCGGTAACATTGAAGCGCATTTTTGTCCAGTTTGGCGGTAGAACCTTCCATATAATCTTCA

ACAGTTTTATTCGTCCCCATTTGGAGTACGGAAACATAGTATTTCCTCCCTCCCTCCAAAAGGATAAGGA

CACTCTGGAACGTATACAACGTCGAGCTACGAAATCAGTTCGGGGACTCAAATTCAAACCTTATGAAGAG

AGCCTCAAATCACTTAACCTTTACCCGTTAGAGTACAGGCATCTTAGAGGTGATCTTCTTATGACTTACA

GTATCCTTAATACTTCTGGTCATCCCCTTAAACATCTTCTTAAGCTT

>CD065344

tagaacaacgaataatcagacaggcaaatatggtaaatcgtaacgaccgttcaacgacaaagaaggcaggtcagtcgctgagaccccaaaacgctgacagacgcatttcaagtgaggatcgaagtcagacaagattgcctactctcccaatttctctttctcttggtggttgactggattatgaggacctcaacatctgaggagaagcactgaagtacaacggacagctcacaatcg

>CONTIG10814

taggcagggttgaccaatctcaccattcctcttcaactttgctattcatgacgttctggaaacagctctgatggatataggtagcgatggtgtggacctgttgtctagagaaagacttctcgacctggaatatgagggtgatattgtcttactgtgcgatgatacccaagacatgcaatccgcacctaatcagttggcaattagtgtccgttggtatggcatgtgctttgcaccttcaaaatgtaaagtactttcacaagactggtagtcgagaagttcacgtatctgggtagctgcataagtgcaggtggtggaacgtgtgatgaaaccaattcatatatggtgaaggtcaaagcggcttatgccaatctggaccatctttggcgtcttcgtgatgtcagtcaggctgtaaaagatcggatccacaacgcaacggtgagagcagttctgctctatgtctgtgagacatgccctcttctaattgaggatcttaagcaacctatcaacattgttgtctccgaaaggttgttaacatccaatgacaacaccatgtcagtaatgagcaagttcggcatcgtgtgcat

>CD078705

ggcacgaggcacacattgaggaaagcagccaattgcgtcacaaggcaagcccttacacagaatcttcatggcctaaggaggagaggaagaccgaagaacacattactccgagaaatgaagacagacatgagaagaatgagtaataattggatagaactagaatggaaggtgcaggacagagtgggttggagaaagctggtcggcgacctatgctccattgggagtaacaggcgtaagtaagtaggtaagtatttacatatgagcttatttgtttactctaaagactgaacaaagtgttataaattaacgaatgaaagttaaagtgggtttgtgaccgaattaaataaattgaactgatattattctgattaaattattttcatcagctatgttaattagtaaaatagcagtttctaaaattttaccattgtaagttaaatactttcttttgtttgataaattagaatatactacttttaatttctatgtcatgaaactaatataaaatagattcaacttataatatacttaggcggcttatgctccattgngagtaacaggcgtanataagtatgatatactatattaaatctttcctaaagtagaatttaatatttanagtgtt

>CF499794

aacgatgagaacaacagcctggattccgtaaggatcgatcgtgttcaaaccaaattgctactacacggatcattgtggaacaagcaatcgaatggatttcatcactctacatcaatttcattgactatgggaaagcatttgatagcgtgaacaggacaacactaatttagcttcctcgacactacggtgtgcccgagaatatggtcaatatcatacggaatacctattatggattaaactgtcaaatcgttcataggagacagttgacagactcgtccgaggtctagaccggtgtcaggcaagcttgcttactctcaccctttctctttctcctggtgatcgactggatcacgaagacctaaacatctgaagagcagcaggggataaaatggacagctaggatccggctgtatgatttaaacttcacacatggaacaaaaaatgcagaagaagattagtgtggcgaaagcctcagcggcagtacgtctcagtatacacaaagggaaaagcaagactctgcgatgcaatacaacatgcactaattgaattacactagacggagaagctatggaagctgtgaaaacctttacatttctg

>CD161137

ttggtcccagacacaatcccattgtcctatgtgagacgcgataacggaataatagtgatgccgttgttcacgacaatcggaaatgttaacaaaccgagtggaggccttttttagggctaggcattacttaattacttaggcccgtcactctcaatggagcataggccaccgatcaacattctccaaacccattctgtcctgggccttcctttctagttctatccaacttttgttcattcttctcatgtctgtctccatttctcggagtaatgtgttctttcgtcttcctctctccttcggccttcaggattccaagtgggggcttgtgttgtgacgcagttgggtgcttccttcaatgcgtgccctatccacttccagcgcttcttcctccactggaatctgatttgttctctcccacagtagaatgttgctgatagtgtcgtcccatgatgtcttcatattcggtattgtccaatc

>CONTIG10829

ccctagtttacgttaatcttatcttacgttttagtggccaacaaattcatcgtcccaaacggatcgatgtcggcaaattggttgcagcttctgttgcaaataaatgtcagactgaactaggttcaaggttagctaccatccaccgaaaagtataggtgagcattgatggcaattgcataacgccatgaaaatggtaagtaaagttgcttgcggcttcgcgaaacgtcccacttataggtacagggtttctacaggctcattacaaatcattgaagcccgttggtctactctgtgtgactgcgagtttgatcataagcgaaggctgttacgtaatgaagttgtgtaaagcttgcgtaaggaccgagaggcctggtggtcggagcgtgccactgagttggaaacagcagttgcatctagtaactgctagaagctctctcaactcatccgagtcactggcagtaagaagtctgatgtgatcaaaacaatctgtagagatggcgggatgccaatcactaaaatctatcgacatcttggactatggacagagttttttgaggagcagtttaactggtctgctgccccggcaacatcgatcagactgtccttctctccatgacctgtgactactgatccaccaaatgaggcggaagtccgcaaggaactccaactcttgaagcgctacaaatcaccaggcccagatgacctaccactggctctttttaaagacgatggtgactttctggctaagaaatcgactgagttgtttacaaagttctgggagctggagagtgttccaaagttatggaacgagtcgatagttatccctatctttgaaaaggcttcacgtcatcactgtaacaattatcggggtatgagtctactttcgactgcgtccaaaatattgaattctgtcatacttcgtagactgtttaatatcacattaagattgacttgtgaggagccagctggttttcgttttggtcgaagatgtattaaccgtatcttcaccctccacaaaatgttagaacaccgtcatacttattacagaccgancatagtagtgtctcttgacattagggccattttcgattcgttggataagactgtgctctgagattgtctattgaagaagggtttgcctgaaaagtatattacatcttaaagccctatatacanacactcaagcaaaggtgaagcatacaaccaccccctcaccatttgttcattc

>CONTIG9989

gcgacatcctaccaacgtatgtgaaagtttccacctgttgcagagattctgcatcaagtgcgattgtgttggtgttgtatgtcttgtatttgaggatgttgatttttcccttgtgtatgctgaggcctactaatgtagaggtttctgccacactggttatcttgatctgatcttattcgtgtgtatgggatagatctgtgaaatccaaatcatctagttgcatgcattctatccattttattccgttcttcctctcc

>Contig3227 EST cluster from Schistosoma mansoni 28/06/2002, 239bp

gtgtagcaccngtacggtatgtgctttgaaccctccaagtgcaaagtact

cctacaagactagcaggattctaatcctgtactcaccctgagtggcgagc

agactgaagtagttgagaagttcgtgtatctaggtagctacataagtggt

ggtggtggcgtgagtgagatcgatgcacgtatgatgaaagccagagcggc

ttatgccaatctgggccatctttgaagccttccctcgtg

>CF498042

cctagatgctacattgaaggaatatctgttatatctgctctttgttggtgtgaattgaactaatttaacactaaataaagtgaataaactattcttatttcacacagttacacaatggacttcgttaacgcacctcatgtttccatggaattgggccaataaatcttatgactaattagcttacttcagcggtactattaagtccttcattatcccggaggattcgaggtggagcccacctcaatatttctcagatatctctgaggtttgttgcagcgttttggtcgcgatcttgaatatgaccacatacacaactatatacacgttgagataacttcaacttttgcagccctttcttggtgtttccacacgctgagcatttctgtgttgatggaaatactggactcgcgactacaagattatagccatacaaattacttttgtcttcaagcatcaatctgaaagtgtaaaagcatgactgatggatagacctagatatgttcttgaacttgatcat

>SMLA29B10

gccagtgtgctggaaagcaaggaggcaattgccatttgtgccgacgttgaagaaatgttcatgcaagtaaaggtccctgaatccgatcaaggagccttacgattcctatggtggcaagagacagacatgtcgaaagaaccatcggagtttcaaatgaccgtccatccattcggagcaacgtcttcgccattctgtgcaaactttgccttgatcaagaccgctcaaacattctccgatggatttgatagctacatagtagaggcggttaagaacaatttctatgtggatgactgcctagtatctttttccactagtaatcaagcaaaaaactttgtcaagcaagtaagtgaattactgtgtaaagggcggtttacattnaagaagtggnataacaactcngtagaagttaggtccgttttgccctgggtatgcnaagaangggctgtgantgaaatgtctagagattgtgatgtcattcatcgtaccttgggggtacatgggatgtgaaagagatggtttccagtttcaccttgacgctccagaaagaacggtgactaagagaagtgatctatctgtggcatcttcctttattgaccccttggggtcttattcttccgtctgtctgaccgtcaacttctgctgcagagatatgtaagtccaaataagcttggaacgacgatccatgagccctatacgtcgatatgggtaaactgggggaacttttgcgacagaataatcccgttaaatacctcccggggatccgaattaattttaatgaacttatgccccgggggaattgccttggttaaagatgctcccaaaattggtatggagccataccttttcccgaatcacttatttgacaaaacgcccaattgtttttgttgtacacaagtccaaggtcgcacctataaaaacagtcactgttccgaagcttgagatggcagcacagttttagccgtaagctaatgaagtgctacagagaagcttaccaattttttctgcgaaggaaattttctactgatcctatgatagggtggattaaattaaaatccggaaaccgatttagaacctattttgctaacgcctgctacctacccacccactaaggtgaccatggactacgtcaatgcccgggaaccacggatggattcgaaggatcaaaatggcgaccttaaccggataaggccttttacgtaagcaatcggaaatttccatgccaaatttccgaaatagtcaaaacttgggtattgaaatttaaatcccatttttacagggaatct

>CF497731

gggttcagagtggggttattgttccatctttttcaaatagtttcagtaattggcgaattcttaatgtttctttgataaatttgtaaagttgtctcctgtcgcatattgccactgacatttttattgattttgatttatcggtacaccaatgctcatgatcactgtgtagactttttcccagtctacgttcaagttgcgttcgctatttattgtgtttgcagcctgaataatctaagagcatcaatgggacaggactctacagtcctagccatggaagtttgttgataaaatacttggacattgagagtttggttgatgagtttcaggtgagttgacgggtgtttgtgtgatgtgtgtcgataggcgttgcgatgtgatttgatttgagttgtgtagatgtgtttgcagatggtttgattgtttgattgttgatttt

>CD061177

ttgctttggcaagcagcagagtacacagaagcaacaagaagtgaagaagagcattggagccgacagacagaaatacatgggagacatgtaagaacagcggaaaaagctgcaagagaaggaaatatgaaacaactgtatgacacgacgaagaaagaagagaagatcgtcaacatcatccagaattcatacgacggtctacactccaaagtgatgcacagaggacagctgacagacgcattctgagtgaggatcggtgtcagacaaggcttcttactttctcttccttctggtgattagctggattatggaaacctccatatctgatgggaagcgcggcacacaatggacagcttaaatgcaactagacga

>CD147614

atggatgtggtgtattttgtgtagaaagctgttaaaaaatgaatgtattgttattggtgtgtatgagacagctgagctaagtgatctccaagtggaagcgacgcagctgagttaaagctatccaccgtactccgtgcttctcgtaaagtgtggaggtcttcataatcaagccgtcagctagcagaaagagaaaatgtgacagtaggcagccttgtctgacaccagtcttcacttggaatgcatcagtctgtgagt

>CF501417

aaatcaaatgtggagagcataatacaagaagaacgaaccaaacaagtttaaattattctcaaactcaggatctagcggtagacaaagaattaatgtatttgtaatattataagaaattatgagccaattaattcaacgtcttcaaaaattggccacaacaatcgtgaagactctaatcaggtattctgcacttgccaacatggctcaaaccaacagtcaaagacgtcattaaccaacatgatgtctcgacctaaccttcaccaacttattcccttgctaaccaacaatgataagagcgactatttatttttatgccattaaaaaaattacaaggattacctgatgacattgttgactttgtttgagtttc

>SMLC35C09

tatccggcacgaggccgacatggttggccgccatcacaagatacttacgcgatgcaatcttataacatgagtttaagccaagtcagtgaaagagctcgtagacgtagggcaaccgtgaacttgtcaagagtcctcggatgtcatagatctacttataacagatgttggaataaattacgtaccgattttttgaggagacatgacttgctttcgtttagtaactttgctattgaaagcacaactacagttgacagagaaagtaattgtcatgagattccttcaactgtattaccttccacaagctgtaccaatgaaaggcttctggaagataatgaaacgtgtatgaagttcatacgctatgctgcgtcgcgcgctgccaaatctggatgaggagtcggaggcacagttgttggcgtctcagtttatcgagagcgtgccagttgccgtttcccagcagctgagattggtacacgccgcgcataccagacttgccgacgagcattagaagtctcttgaggacgtgcacaagttgttggcagcgttgcaagtgtgctatgctactaatgagaactcttcaccggaagaaaatacgttgatcttaaacacggataagcaaaaaagaagtggcttggaacggggaagaaagttttcctttacatttccggggaactgggatgtcgcagagatgcaggctgggaataaccgcgtccaatatccaaaaaattcggaaggatggaaaaggtggtcctctcgaattgccaaacgtttggatccccaatgggaggggaagaaaaaagggcggacatgtcctccaaagccgggaaatataagggaaaattaactacatgggaaatgacatagtttatccgagaaagaacacaataatataaaaggagtacttcgagccaacgaaactcacaatactttcgaacgcgcgcggggctcaccaacgcttgaggagggccaacgtcgccccgccaacgcgcaacgcacgggagttcgaagagcggcggcagaagagcgccccgacgggcagcaagcgcgagagcgcagactgaaagaacccgcaaaagcgagccacgaaaagtagaacgacagaccgcactcagggagagcacgagagagcggtagcaggaccggaccgacaccaagcgcgaccacacgag

>CONTIG1505

tataacgaaatgcgcgtcctcgattccagtgctagccactattcatctttgctgagaattatttattgtcattccggccagaagttagattcaatgattgtaatcggataactattactgacaggcagatgtcggaattcttccaatttgttttcagtcgtgttcacgatataaaagccttgataacagagggacaatactttttctcttcctgcttaaagggatctaaatatggttctctaaacattagttatatgtgtgaaattattgagcaaaatgtattgtgcaaccagtctgtgatgtttaaataaatcaatgacttcttcgtattgatgactgttgtaattttgaattccaaatacaatacattcgttcgggctcatctaatacttcttgattacattgcgttattccggggattactagttcatactacaattcaaatacttctaattatcatattggcgtcgcgattgagcctgtgatggaacggttggatataaattcagattttaatgcttttgaggagtacatggagaggtttgagatatggagcatgaccaagaaagatatgaagggtgacaaaattgtggcacattttcttacattcatcggtcaagaagcctacagtttattaaaaaccttggcatatccagataagccgatttcactcccatatacaactctcaaggaattactattaaaccatgtgaagtgcaccagttttgagtgtcgtgagagagcaaaatttcataagatgattcgtcaggataaccagaaggttaaagacttcatccttgaattgcaaagacaagctgccaaatgcaattttggtgatcagcttcatgtacagttgagagatcggttaattgctggaattaacataccaggtctggaaaaggagctgttaagaatgccgaactgttcttttcaagatgctagaactgcatgtattaactacgaagcagtgaatgaacttgatattcaatcgatgaaaatttctaatactttgcttagtcgtcatgatgaaatacaatctcagggtcgatcaaatttgcgttcgttcaatcgtgattgctattcccgtggaaatatgaaaggtggtttaacaaggaactgcaaagcaaacaacaaaggtattggtgcagttttggaacaggaaggtaggccagttatatgtgtttcacgcaaactttctgtttgtgaacaaggttattcacaaacgcagcgagaagcgttagctgtgttttgggctgttaaaagacttcataaatatttatttggaaagaagtttaccattgttactgatcatgaagctttaaagttcatttatcatcctgacaaatccttagcacgttcctcagctgctatggttcaacgatggagtattgctttgagtgcttatgactatacgattcagcacaggagtgcaaaacaaattcaacacgttgattacatttctcgacaaccattacaagatagacctgttaatacttcagactgcttgttagtgcaacctttaccggtgagacgtccagatctcattagagaaactcgtagatactttggatgtatactcagtgctatacggaagggttggaatgctaatctgaaacgtagatttcctgtctatttctcaggccagggacgagttatctactactcctgacggtattttgtgtttaaatgatcgtgttgttattcctccttcattacgcaaacctgtccttgatgatcttcatagtggacatcttggtgttgagaaaatgaagtccttagcaagactcacatgttggtggccagagataaatgcagatatatgtcgtacagcaaacaattgtgagaaatgtcataagctaaaaagtctgccttcgaagtggactccatggccagtatcgtctgaggcctggcagagaattcatgcagactactgtggtccatttcttggcaaatactatgcacttgtaattattgattctttttctaagtggcctgaagtttttttcacaacttcaccgaattctgacttcacaattcaagcattaaggaaggtgtttagtcgagaaggagttcccatggtgttggtgactgataatggctctcattttgctgcagatgcagtcactaattggttaaatggtatagggtgcagacatctgtttacggttcccaggcacccttgttccaatggtcaggcagaaaatttcgtaagaacattgaaaattgctattgattctattgctgcttcgacattcaatgaactggagaggggagtagatacatttctacttcnatatagaaatgcta

>CD178149

cggataccattagcaaaagcatattataggagagaacaaaccaacttacagttgaagaggaaatttggaaaagttactgtaggtggaaaggacaaacattgtggaaatcatcaaacttcatcaagaaacaagcaccaacttggaatcctgaatgccaaagaaaaaaagacttggaccaagaaacacatttcgtcgggaattgaaagcagacataaaaaagttgaatagcaaattgattgtccttatgtactacatcatcatctccattaccattatcatatcctacgtcagtttgaagtaggattttaaaatgtcattttacaagtttgtaagcaattcattttgaatatgtttataattgattctt

>CD085463

tattcgaaataactggtaaattgaatattttatttcggcgaaagagatgttctattccattatcataacaacattaaattattgttttcagaccattatgactatatatatatgcgtatgttaccactcctgcagcataggccactgacaaggattcgccaaacaactctctcctgaactctcctctccagctatttacacatactattcattcttttcatgtttgtctcacatttccgactcaatgtgctctttggtcttc

>CF499013 (CL1156)

catgtttagactctgatgacgctttaatacgagcacgcatttgtttgcgcctcaatggacgcagaaaaactacactaagaagacccattagaattgaacttggggacaataaagccaaaagtaggttacagaaacaaatgagttcacattaggcagttctgaaaatgaggctgacccagacgttgcttggaaagatatacgaacagctgtgaaaacagcagcaacatctattagtaatttaaaccaatggattcctacttagtctattgaattgatggattctcgtaaactcgtcctatcagcctctgaacacgatgaagagcgtaaacaaatcagatctaagttaaccaatagtctacggaacgatcgtgagtagtggtgggcagcaaaagcaaacgaaatggaaaaggcagcgtttgtaggcaacaccagacaactcttcagactaataaaagaaaccggaatcaagaagtcaagtgtaggcgagacaatctcggaaaaatgagactcacctgctctcagcccagacgtttgaaacgatgggcggaacactttagagaacattttagctggccctcaact

>CONTIG10886

acagatccgcaccactataacctacattcatcacagctgtttccagaaagtcgtcgatggcaaagtgaagaggaagaggttgggcaaccccgcctaaccccactgtttgaatggaacagtagagagaggtggttgtatcccatcactctgcctgaggtgttagtaaataaggcttccaagatgtcaataaaattttcaggcacacccttcttcagtagatacttccagagaacagtcctgtccaatgaatggaagacggccctaatatcaagaaacactatgattgttggcctgtgataagtatgacggtgttctaacatttggcggagggtgaaaatgtggtcaatacatcacagacaagaacaaaaaccagcctgcttctcgctagtcaatcttcctcgggttttgaataatctacggagtatgatgaaaacttacagtttggatgtaaccgaaagtagacatattccccgatagtttttactagatgacttgaaccccttttaaagatggggatgactatcgattcattccacgatgttaaaaccctgtaattgccagacatttgtgaacaactta

>SMLA45H10

gccagtgtgctggaaagtttttttttttttttttttttttaataataataataatatattcctgcaatagcaggtacacgccatttttacaggcatgatctacaatttacaacatatactggttcacagtatgcatcacaagcaaggttgtttagtaattataatctataaaagttgatagcagttcattcgttcagatataatgtgttttcataagaatagtttccaaaggggcatagcgtccacgttacataccagatccaatctcgacaaaaagtgtaatagggtttcggtcgtgctaagtatttatgtaatgcagctttttaagtttattgttgttataaagtaggcttgtggagcgcctggttcgagctatgaccttgggaaagcgcgtgcgtcgagcttgttccagatgtccgaagttacatagcttcaccctcagagcaagattctgaagggaaaaaaaagaaaccgaggagcagcaaggcatttggatatgaacgataaccaatgcttaacatttattagtggaacagatggaggtatgataaattattagattaaattgatactagctaggtatacgtcacacgtggcccaaccatctcagtcaatgtagattcacaaccttatcaactgatttagcattattctctaataccctgtgtctaagcactccattacttacccggtgatcccagcagatgcgggcaatatttctaagacatctgtgatcgaatactagtaatttacgagtatcttctactcttaatcgccatgcttcacagccgtaaagtaaaacagagcgaactgctgcgcagtatacttgtccctttattgatagacggatatctcgtgtttgccataggttgacgtaatttggcaaaagccaaacgagccttttgaatccgtgctttggaattttgtcagacaccaaccccattaaggctgatcattcttttcatgggggaatttaatcttaataatctatttctaaaccttgtgtaccaagatatttttctttaactattacaacaatttttataaagttatttattattctacccataatccacccattatttcttttttttatcaagggtcaaccataactacttaccccaaaatttatactttcctttaaatttcat

>CD068213

acttgaagtcgacggtactgagttcgaatcacggaggtgaacatcaagtgagatcccggtatatctggctgacgaatcctgagtaagacaaaacaacaagcgttctggatcttactagtcactgcagtccatctattctatgactaactgaagagatgacagttggatgagcacaggctacacaccgatattctagaatgttccactagcggtgattcgatgatatgtcttcggttgtttctggacttcttgaggcttctttgagtttgacaacgagccatccttacagatgagcataggattcttatcagaattttctatccaatcttgttctggacaatcctttccacttctttcgagttgatattcatccttttcatgtctgcttctaattatcgactcagtgtgttctttaaccttactctttccgtttccattaaacattctcagttagagattatctcataatgcagtttgcatgatttcgatagaccccatccccacatggatggatgaatgtacctttcaccca

>CONTIG1185

gaggctgtggagttgaaggcctgctacttccccattcttgatttatctaatggccatcctgattccttcggttgtggtggagtgacatctatagggaaggtctataggtgccgcttcgatgtccggtggattcagtagagctggtctactcaaccgttcgtcgaagtgtcttactcatcttttactctgctcttgaatcccagtaattgattttccttgtttgtccttgaccagcctttctgtcttactatgcttccgaagcagtttcttcattatgtcactttatttttcatatttcgttctcttgcagctttttcctctgtcgctgctaggtcttcgatgtatttctgcttgtcggccccaaggctcctcttcactcacttgtttgcttctgagtattcaccttgtgccttgactacctctcttcttgttcgactgctgctaattgctatcttcttgttcttcctttcttgaatcttatctacggtcccgatagagacctattacttacgatgatgcttattgaggcccagaacctcctgacacgttgaaagcagtgcttttttcatccctttctagttgttttctatagcagtttcctctttttgggtagatcttttacgacttggaaccagttgttcagttatcttgaattggttgagtttgtcagtatctcgaaggaaggttgtattgaaccttagtaatgctgttaccccaattgtccagtgtttctttagcttcagtttcaccttggcaaccaccaggtggtgatctgaagctaagtcagctcttctcctggttctcacgtcttccattgacgttatgaatttttcgctgacgcaaatatgatctatctggttctctgtgatgtggtgtggtaagacccatgtagctttatttatgcgtttgtatgggtatacttttctacctataaccaatttgttgaatgcgcataaatctgcaaacctgtcaccatatccgtccttttctcagtttatgtcatcccatgataccttcatatcccgtgttattcattccaacttgggtgtgtaagcctcccaccaagatggccagttcctttttttgagcacgtcgctatgatcaattgcagtttctcgtanaactgatcgttatcgtcgtcgttgctatcatttgtgggtacatanctatggataaattcattgtg

>CONTIG4517

ctgcccaccttagtacctctcacctgtagtcgaattcttgatctatatgactttgttatcagtataatagcactgaatcattgattaattactgattcgttgatttgttttcgatctcagtccgattcatacctgctataatcgttatgtaatgtcactggtgcacgtaaacactccataattgatttaattgctcattgagaagcttaggctgcccaccagcattccccatcgcactatgtcctggtgaatcctttacaattcctccagttgttattcattcttttgatgtctgattccaattaccgatacaatgtgttctttggtcttcctctttcttatttcctttcaagtttccaagttagctcttgcttcgtgatgtagtttgatgatttgtgcaatgtatatcctatccaccaactccatcatgttttcctattgttggggatgatatccggtcaacggacattgattatcttgagcgtacaattgtttataaatatttgtgtctttttgatgttggttctagtagttcatcgaagtttcagttccacaaaatagaactgtcttgacgttcgtattgtagattgtgtgtttgatattggttgatagttgttttgagtttcatatgttcttcaactgtaggaatatcgcatcttattcggcacatgtctctattgtgacaatataaaaagtacctgtttgtgtgta

>CD094029 (CL1177)

ccaaattccattcagcggaggaagaaatcaggaagaagcactggaactgggtacgacacacattaaggaaatcacccaactgcatcacaatgcaagccgtcacatggaatcctgaaggccaaaggagaagaggaagatcaaagaacatattacttcgagaaatggagacagacatgagaagaatgaacaaaaaactggataaaactagaaaggaaggaccattgtagtgtggattggagaatactggtcggcggcctatgctccattgggagtaacaggtgtaagtaagtaagtaaaaagcttaaaaagctatttggtctagtttgattctctgtaatcctgtctacaaactcttagacagaaattcatacctattacataatctattgactactcaaactttgtacttcaatatcaaatgcgttgtcnttgttgtaggggggaggagggctatttgaaacaaaacctatcaaacttcacacgacatagtaaccattcaccctacacttcatctacattcaatctccaatacttcaacttctgtacaattttttttgtatttnttctaatttttttttgttttttagtttaaaaatggatc

>CONTIG7383

ataatgatgactgaaaagaactcttgaatcaaccagccatattgaacccaccccacatcaaagaaacacccacggatctcccaaccaatgttggcccaccaataattgaggaaatcagcatggccatcagacaaatcattagcggtaaagcagcggggcaacatcgcagcagggacactgaaggcagatgtagcagtaactgtaaagaaactccacattgtcttcagtaggatatgggatgaaggacaagtaccaacagactggaacgaagggctcacaatgccaaagaaaggcaatctcagcaagcgtgatatttactacgtctaagtctctgatcttcaaagtaacacagataaactttaacccaaaatcttagctagttgattcaaagaatggttacacatatcatcatagtgttttcataacgtggaatctagaaacgatagatacgcaactctttattgcattatagatgatattcaagcgtaatcgaacttaaatgcaaatttaacataatacttaccgaactcattccattgtgagcatccccagcacttttgggacgaatcgacgttttgtgtggaactttcaattgattgttatcattataataagacagagtatgaaggcgattctcagcaatatcatctaactcatggaaatcggatgttcttggtgtttt

>CONTIG10814 (CL123)

taggcagggttgaccaatctcaccattcctcttcaactttgctattcatgacgttctggaaacagctctgatggatataggtagcgatggtgtggacctgttgtctagagaaagacttctcgacctggaatatgagggtgatattgtcttactgtgcgatgatacccaagacatgcaatccgcacctaatcagttggcaattagtgtccgttggtatggcatgtgctttgcaccttcaaaatgtaaagtactttcacaagactggtagtcgagaagttcacgtatctgggtagctgcataagtgcaggtggtggaacgtgtgatgaaaccaattcatatatggtgaaggtcaaagcggcttatgccaatctggaccatctttggcgtcttcgtgatgtcagtcaggctgtaaaagatcggatccacaacgcaacggtgagagcagttctgctctatgtctgtgagacatgccctcttctaattgaggatcttaagcaacctatcaacattgttgtctccgaaaggttgttaacatccaatgacaacaccatgtcagtaatgagcaagttcggcatcgtgtgcat

>CD201986

aatgtataggagaacaattgggcatgaaaatggcgaacccaacccgtgggaacctatttctctttaaaaacgctcaccagaaaaactaactgaaaccatctaaactctaccctgaaagtcgaatgaagaattatgacgcctcaggataaggccggaaagtacgaggccgatgctattcttgcaaccggatcaaaaatttttataggtacattgaacgtcagaaaaatgtgggacaccagcataccagtcaaatagccatggaaaggaggagatacaaactggcagtactttgaatcagcgaaactcattgaactcaagctgaacagtagaagctagatacgggagagatgatgctgtacttcggtcacgaagaggaaaatgctccacaggatgtttctccaatgatgtccaaagaggcacgaaacttacttgttggatg

>CONTIG12858

cactgggtcctcttttttttcaattggggtggaatattcctttggataagttccgttggggggttggtacagactgccgtcatttggagtcctacctggcttttaaaatgcccggttggggccaatattcccttattttcagtggccaagaattgatcgtcatcaccggattgatgtcataaattgatgcaacttccgttgcccgtagtatcgaaccgagttagcttntaggctagcccctatccccttgaaagaattagatgagcatggttgcaactgcatgacggcatgaaaatggggagtaaagccgcttgcggcttcgcgaaacgtcccgcttatgagcactgggtttcttcttttttctcatatgcacctcgctcttctcttttcccttcccanttttctcatttttttgtgtggcgcatttatatctggtgccactctgtaccaatgtttatgtgttaaaataaataagtgaatgagaacacaagtggggacaatcgaatgtattttaatacattgcagaatctcttagtaaaatctgagaaccatataataaatccttcatttgcaaatgtcaatcattcgtctcagacttcaccgttccttcatttccactgcaatcattctctgttttcgttctcttttcttcgatcttcttaaccttctgctgccaggtatttcactttcgattgatgatgcatactaattatgtccatcgacatcagtagcacacaccacacagaaacagtaacactgctggatattaattattaaacagaaaagcaaattcggtttttttttcgatatttaaattgcttattttattttttttttatcgcaaatatgtagcgtctatcttcccaaaatgtataaaatgttgcagttattccattcattctctcccccccccaattttatctgtctaaatctgttaagctttttgtttacaataatggtaattttatagacttacgaaatttatagttaatattatgtgttcatatattcactcaccattagtttatttttttttgaaaagaaaggtgttatgccctacactgttaacaactttatcagaattgttcagtctaaaataaaccacacactttaataatatgctgcctttcactatgattagtattattctatatccttttggtaccaccctaattatgcatattagtctctgtattctataatcacttccagaacgtattcttctttcaccaatctcagcttgagtatccaagccttatcgttggctttgtaacaagttaactatgactcaaacc

>CONTIG10810

tggatacatatgcgctacacaagccacttgatctatgtcggctatgatactgcccgggtacttagaccgaagcagttggttttcttagtaggcgacatccagagccttcgacctaaaggtctagtccacaagacagtggaggaacgtaagaagatgcagtcccatggtagccggtcaccaacaataggttcatacgccatttatcccctcgggatcctggagcccatgtacacacttggtttggaatcagggtttttcaactcccctaggtagacttcccgtgttcaccaacccggttaaagcgccggacattcgcttttcgtcctctcaatttcgtaaacaacacccatgctgcgagaaggcagtgagtagaacttccgtgacagtgtgtccaaccaatgcctaccttgacggtcagtgttttctggcgtaggagtaccctatgataaagctaggggcggccaaaccaaaacaggatatgaccccatgaaaccactaacaattaaactgagccatggtaataggtacagtttacctggttggagttcggttgattatcgtaaccagtagttagatactttaaataacatggttcaaaattgtttgcaattgtgtaggtttattcattctttgttttgtccccaaa

>CONTIG9225

catgtttgcagttacttcaatgtctgacttcagtggttcaggtggtatattgtcaggttccgctgcctacccactcttgatttgtctgattgtcaacctgatttcttcgatcgttgatagggtgtcatatgtaggaaggtctgtgtgtgctgtttcgatgtttggtggattcaatgaatctggtctattttagagttcctctaagtattctacccattttttctctatccttgaatttcagtgattgtcttgacttctttgtttgtccttgactgacctctggtttactatatttccctgccagttacttcttcgtgtcatatagttgtttaatatttccttctcttgaagctttctcctcttgtcgactttaatgttctcccttctttgcttctttgctttagtgtattcagcttgtgttttgactttctccgttcttgttcggctgatgttaattgttgtcttcttgtacttcctttcctgaatctggcccatggtttcgatagagatccatttcttatcatgatgtctcgttggggcacagaacctcctgacacgctgaagttaatgattctttcatccctatccaggtgtccgccagaatagtttctccttgttt

>CD064574

accccggtatcagtggttgtaagacgcggatcgataaaacaagacagcatatccacaatttaagaacatttggaactcaaaccaattgtcaaccaacatcaagatcagaattttcaatgcaaatgtcaagacagttctactctaccttgagaactacgaaagccatcatccagaagatacaagtatttattaacagttgtttacgcaaaatacttcggatccgttggccagacattatcagcaacaacctactgtgagagagaacaaaccagattttagtggaggaagaaatcaggaagaagcactggaagtgaataggacatacattaagggaagcacccaactgcgtcacaaggcaagccc

>CD094930

gaatgtgattgtttttattacgaaacccgtagtttcgttgaatcatattgcagttcgtacagtcgacttcgtctatgtttgtcagaatgaaaataatctaaaggcattttagtttaacgattttggagttttgacgtttttcacttcattgaattaaaacagttgtggatacacaatacagtggacagcttggatgaaactatacgaattgaaattcacaggtaacctagtccttctattccatacacacgagaaaatgcaattcaagaaaaccagtataaaagcagtgtctgcaacactgggcctcaaagtaaacaagggaaaaaccaagatcctaaaataaaaca

>CONTIG2057

gaggatgaatgaaatttctatatttatatttcaaaattagccacacatcaaatacaagcaatttattaaatcatacagatatcacatgtgcctcagcttgatttatattctaaatttatgtagtctatttatcttctccttctaggttcattgtaagtctgccaaactgtgttttcgatacagactttgtattgtctaaaatatgttcattataatgaacattgtaagtctgccagactgtgttttcgatacagactttgtattgtctcggtcttggaattgtacaagatcaagtggtcggttgtttactggaatcagtgatatatagtattcggaccacagatttcagtttcagtttggaaaggacaggaggcttgatggcctgtgttagtcctggcaatgatggtctctcagctgatccaactttcttttgaaggagtcgacggatggagcctcaaccacgtgctgaggtaatgaattccactcgttgattattcgatgggaaagtcggtagtcagctgacaagtaattcgttcggggcttgtgaacttttttggagtgtcctcgtagattttctgttttggaagacaagaaaaatgagggcatatcaggtgcaaatttgtcattaagcaatttgaaaattgtaatcaagtcgcctctgattcttctatatgacagcgggaataggtttagcttggtcagtctagtaccatacgggagcttcgctatccgggaatcag

>CONTIG11098

catggtagaacctacagcaacatatgtcccagccaacatagctcggttgagtcatcacgataggcaagaccgaccaccacgtcaagttatcagctacattcgagtataagtggtcctcttgatagatcaatgcccaaaaattcagttgatgagaaagttatttccagcagtaggtttatggtgaaattgaacgaaaatggggatagtggacagccttgacggacaccacttgaggttgcaaaatcagatgacagttcgccataagctctgactcgactggtagtgttcgagtaaagagccttcacaaggtttatgtacttctgaggtacacctttcaatgaaagacactgccacaaaacctctcggtctacagaatcaaatgctgcttttaagtcaagaaaaactatcattgtcggatgccgataagcatgtctgtgctctgcatgtctgtactgccacataacctctcggtctacagagtcaaatgctgctttcaagtcaagaaaaacattgtcggacgccgataagcatgtctgtgctctaaaacttgacgaatggtgaatatgtggtcgatacagccacgaccaggtctgaagccagcctgattttctcgtgtttgcagttcacgagtcttagttaggcgcccgataattattgaggctagtattttagatactatgtcaatcagactgatccctctatgg

>CD097038

acgactaactgatgcaagtttgctgtcgttgactttgacagggatcgatgatctgttcaacatttagtgaccggatgacttggctagtgctcagtgacattttactgccccttcaataacattcattctgattccctcattctttgttttgaaggatgatttggtgatcctgaatccatgagattcccatctcacaagtatttcttttttatgaccggagtacaacagcatctatcccaaatctatccttttttgtccaatttggatccactgggtttcactaaatttgagcaccgtcaagttgtgcctccttaataccgcagttatttgaaaatcatcatcgacttgaatagattgatcaaggtcaaaaatcgatatacttatatgtaaaaatagatatatcaaggtcacttgatcatgatgcttaatatgtttaggacaatgaagagaagaaaaacaaattggatcatatttttctgatttgatcagaaaaactgactcctataagcagcataacatattatttcgaatgagtactgttcccttggcatcaa

>SMLA22B05

gccagtgtgctggaaatcgcaataaaactttaccaactaagaaaggtataatcaatcactggtagaagtagtaagagtataagtattatgtgaaagataagggtttgaagatgttattgaaggagtaaaatacggtgaaacaaatttggagagagaaaaaggatacgaacatgaagaattcagaagattagaatttggtggaacacaaagagtgtatgcacctacgccattgcaaacgattttgagccatgtccctaaagtgctctgctcatcgatccaatctcctggattgagaaggaatgatatgcccatccttgtctgagatagtttcgctaatagttgggttcctaataccggtttctttaacgagtctgaatagctgcctactgttacctattaccgctgccttttccatctcttttgctttcgctacccaccactgttcacgatcattgcgtaggcttcttattagccttcgcttaagctgactccgctcttcgttatgttcagagccgggtgggatgagttttcgagcatctatcagtgtggtagatgctgtcgagatccattgtttctccctgaccttatggtttaccttaactacaaaagccttcgacactttaccaaagcaactaatacagaatgctctattaaaaacatgccgatcccgaaacataaacttgtgcatgaataaactactagacaatccaaaaggcttcccttcggggacgtaacccatatatgcgacgatgtgctctggctggagactttgaggtcactaccatctaatgtgccatatagggcgcgttttccacattccgtatacgtcatcagaatgtgtttatcgtcctcccgccatgagacacaccgcctaaggactttgccccgataagatttgtaggccgccngattttcggacggcttccnacattcgcaactacgtgtgcnaaacgaagcagntcggcgtgatcgtcgaagaatggcaccggaaagaggggttccccccgatttctctatttctatgcgcgcatgtgggggaccctttttaaggggcgggggagaccctaacccttttaaataattggcgaattagaaatcaatttttaggggcccttttcctttcccaaagggggggcgtttaatggtgatccacgttgcaccattggggttttgttgt

>CONTIG3564

aacaccaaacaaatcgctctcgataaaaaaatttcggaagagatcgaagcacttacttgcctgggtaacatcattgattaccaaggaagacatggtgcaaaagtcatggtactaattagcaaagcaagggaagaattcatacaattaaaaaatatccggaactcaaaataactgtaacagaagaccatagtcaggatgttcaaaactaatgttaaactgttctataatatgaagctggaatccggagaactactacaacaattgacaaaaagcacaggtacttataaacagttctctacacaagataccctagatccactggtcagacaacataaataaaaatctagtttgacggagaaaaaaaatctatcaggtgaggaggaaattagatgctggagatggatatgacacactttacggaaactactaaactttatcacaagcactggttcagaattctgaaaatcatactagaggaggctgatcaaagaacacatggtgcagggaagtggaagaagacaccaacaccatgaacagaacttagcagcaagtggaaaggagagttcattatagtgttgtttggcaatccctgtctagaattcagtgtcccaagaggggataccaggttcgaataagtaaaccagataatctttgtaaaacaaactctggatc

>CONTIG11000

gctcctcgacagaagatggaagtagttatttatcagcagcactgtattctcggtcttggataggttcataaaacaaatttgcatgtttcaaggtgtttatctagttgtacgcgatagttgtgttcacgtggagatactgctttaacgtcttgcatagtttgtattagtcgactgacgaaacgagtaggatctcctggagttgtagatttatgattaactagttggccgggtaatgttaatcatgtgccgtaaactaattcggcagatgtacatcctatgtcttccttcacagtggcacgaatcccaaggagaacaagcggcaaggcatcactccattttgatgaatcagtttgagccatgagtgagctttttaattgacgatgaaatctttcaaccataccattcgctgccgggtggtaagctgtggctctgatgtggtttacgcccagaagtcttgtgaactcctgaaacaggatagactggaactgaggaccacgatcagttgtgatggttgtcggtacaccgtagtgcgatatccatcggtctagaaaaacggatgctacagtctcagctgatgtgtctttgacggggcatgcaatggcccatcgagtgaatctgtctactgccgtgaggatgtgggtaaaagaatttgacgaaggtaaaggtcctactaaatctaaatggatgggctgaaaacgtctatccggaataggaaattttccgattggactaatagtatgcttttggatcttactacgttgacactgaaggcaaccacgtgtccaacgtttaatatctgaattgattttcgg

>CONTIG11078

cctttcaaccttcataaattagccagtgatccttatcagtttgctaacaaatgcaaaagaagcaccttagatgccgtcgcggttctgcatcacaatatagtgttcggtttggaaaagggtaaaaagtatattcgatgcgcttttctgaactatacttctgcttttgattctatcccaagacaactttttcttagtaagttaatcagcgtcaacactgacagctggataaataaatggctatgctcccacctctctaaaagagaacagtacactgtgtttggaagaaagtgttcaacgtctctactgtgtaatgaaggtgtgccacaaggagctgttctcccacctctccttttctctttttttctgcatgatcttccacagaaaacatttttgtgaaatatgcgaacgatttcaccgtatgtatgccgatttctacctctttacatcccatagaaatgaatgagtttttgtttcgtattgaatggtggtctgctggtaatggtcccatacttaatccgtgtaaatgttaagctgctaactttagcccgagacatggacagcacctatacactattttaggatcccataatgcttgtgtcattggagactctttgataaacacagtgtcgaaggtcaattatcttggtgtgaccttttcctctgatctctcttggtcttttcatgttttactgtaatcgaagaaagttttccgtctgacttactacataaagaggctgcacgtttttgggattactcgacattta

>CONTIG11047

aggctacgtacgggagagaagctgctgtaatacggtcacgatgagganaatgcttcgcacactcggagtgttgctctgatgatgtcaaaggaagcacgtattacacttatacgattgggaatctcacgggtccaggatcatcacagcatccttaaaaaaacaaagaattgaatcacgatgaatgttatccagtgttatgcacccaccaatgatagcaacgacgacaataaagatcagttttatgagaaaccgcaatcaattatagcgaagtgtccacgaaaagacctgatcatcctgatgacagaccttaaaggccaaagtcggaatggacaacaccggatatgaagataccatgaaacgacatggactgacgaggagaaaggaacgagaatagggacaaattcgcaaatttatgtgcattcaacaaaatgattataggcggcacaacattcccacacaaacacatacacaaaacctacaagggtatcaccggaccacaatacacagaatcagaccgactatatttgcaccaataaaaaattcagaaggtcaatggaagaagtaagaactaggagaggagctcaaatagcttcagatcaccacttggttgtggccaagatcaaatcgaagctaaagaaacactggagagctatggaaacagcagtaaaaaagttcaatacagacttcattcaacaaactgnacanacacaggttccaac

>CF500464

gcgcactgctgaggagtcccataataggacgaaacggccgtccagtgtttccaggttttccatggtggtctagcttcaattgactcatgctttctactatgaaaaatactaaatctccacaagaccccttctggaaataccattaagcctgttaaataaacattttatcaaaccccgtttgtattgcacagggcaataactataataactaaggtattgacccatccatatcggttttcgaaaaatggatcgtttgattgagccatcttctcgtctgcttagaagtatatccaggaagggaagctgatcgttgttctcctcttcacgtgagagactgatgtggttttgaatagtgttaagtttattcaagttcatgtcatcctttctttcacagacaactaagatatcgtccacatatctcttataaaatgacacgttttcgattaagtcttcagacagatttccgacaagtgccttaaacatatccgctaataatggtcctaacggact

>CD091392

ttttacgtacctggactgcatcatcgataaagaagaagattattatacagatgtaaatacaaggattggcaaagcacttgatatgtatttataaacaattgtctacgcaagatactcagtgttcgtcaacagtctactgtggaagagaacaagtcagcttacaactgtagaggaattcagcaaagcacactaaaagtggataggatgtacattgaggaaagcatcaaaatgcatcacgaggcaagcactaacttgaaatcctgaaggggacatgtaaagtggaagaccaagaaacacattgcgccgcgactggaagcagacatcaaaagaataaataacagctgaaacaaatggagagaattgtataggtcatagttggatggagaatgcttgtggacggcctatgctcctccacgagaggtagaaaacacaagtaagtaagtggttaatatgattttctccacatgactggttgaactcataaatacctatttggacgataagaccaggaaacatattcttcaatttangaactggaaaacgacaaatagatattatctgcatacgaagtctttttcacatccctgcttagtctatcgtatcaaacatgactagnt

>CONTIG3407

aaaagtggaaggccaaagaacacattataccgggaaatagaagcagatatgaaaaggatgaatgttaactggaaagaactggaaagaattacccaggacaggattggatgaagaatgcttgtatgcggcgtatgctcctccacgaggggtaacagtcataagtaagtaagttaattgcgatcaatgatgcataagagaatcattctaattccctacaacttgcttacatcatataaataaattctattcattatacactaaaaacacttgactacattattcaactacttatataaagtgtgaaaccattcaaaaaaagaagataaacagaaaactattcaatgatcatttgttcaatcattcaatcattgaaacattcaatgttaaatgtttattcaacagagtgagacaaacaaaacaacctacaatctgattatctaaacattcaacaataaaatctagtaatcaatacattcgtatacctttttataacctgaatagctttattcaataataaataatcaaatctataggaaattaaataaaaaagcgttcatagttgctaggatatatttcgatatttaccattctacttcagtttattatccttaatagtatctatgataaaatacaaacaattattatgcattgtaatattgacatttaaagtaatgattg

>CONTIG3698

ggatgaatagcaactggaaagaactggaaaagaaggctcaagacagagttggatggagaatgctggtgagcggcctatggtcctcgacgaggggtaacaggcgtaagtaagtaattgagcacggtcaatcgttttctaacttctatgaattcatggttaaaaactctacctcaacacggatcacgtttcacaaatctccgtcataatcaattttcaacttgcattatgtaatatactactgtaacattgatattaagaaatagcatattttagtaaatacttataatcaatcatatcaaatttcattattccttcaatttatttttattcttatgtttttctttttttagaaaaatgctcatggaattcatttatttgaaaaagtttgcaaacaattagatttacaagaaactgaatattttggcttgacatatcgaactagcttacgtgtagatgtaagtatatcacaatactattatttaaaaaacgaagtgattatacgtaattatgtaattcaatcctaataattaataactgacattggattggttgaagttagacattaacaccgttggatgccggccggctgagtggtctagaggttaaacgctcgtgcgcgtgactgata

>CONTIG7033

ttttacccatctgttcttgaatctcagtgattatctttccttctctgtccttgataagtatctctgattgaccttattttaccctgccagtttcttcgttgtatcatatggttgtttcatattcccttctctggcaactctntccgccgtcgttgctagttcgcccatgtatttctgcttgttagctttaatggtcttcttcacttccttgtttgcttctgcgtactctgcttgctcgttgactttctctgctcatgttcgactgttgttaattcctgtcttcttgttcttcctttcttcaatcttgtccagggtttccacagagatcaattccatatgatgatgatgtttcatgcggcccagaacctcgtgacacgttaaagttaaggcctctttgattcctttccagttgtcctccatcttagtttcttctttcattagatcctgtagagattggaatctgtcgttgagagttatcttgaattctttgagtttgttagtatctcgaaagaaggctgtattaaatctttgtggtgccgttacaccagttgtccagcgtttctttagcttcagtttcatcttgac

>CD085433

ccatctggacggtcatttaggttactgggcacttcaccatgatcgattgcagcctctcttaaagctgatctttaccgtcatcgttgctatcattgctgtgtgcatattattgaataacattcattgtgattccctctttgttttgaagggtggatccatgagattccaatcgtataagtgctctgtgtgcttcttgtgacggcggaggacttcttcgtgactagaaagtagcaaattctcttctgaatgtatgtttttctgtctagctggggttaaatgggcctcacttattcagagcacatccaaattgtatctcctcatttccgtagctatttgattaatcttcccgatcgcctatattgtccggacattctatgtacatataataattgttgctgtggttgttagaaggggcatcggcatcgtgactcccgaagaatctcggctttcactatgtgttataattcttctgaaagaagatccgtcccctcggagggcatagtttagatggtttaaattgtttattctggctagtgtttttctagcaagattgttttctgggggatgggattgctgaccccatacctaacc

>CD187909 (CL634)

cgaacaatatggcatacatcaactgctaatgaatttatggactgatacgacttcttggtctgatcatcttcagttatcttccatcttaatcctttactgatcaggattacacatcaaatagacggtgaccgggtttacgcgacacctgtacgtcccatttcagttgaaaatctacagcctcataaactgacttgccatctgtgtgtagtacaacatcagcattattcactatgtcatcctaaagtacttcagtaactctccgaagacactcacaatcagaaacaaggaaaagaactagcatagcttcatatctctatctggtggttgccaagatgaaactgaagctgaagaaacactggagaactggagaaacagcattacaaagg

>CD178757

ttatgcgatgattgtattgtctactactgccggtgctaaaaagctgactaaaaaactgcacaatcagtattagatatcacaattcgataagtatttcgaggattgcacgacagcgaccgatggttagagaccctgagtgatatggctcaaactcgtttgcaatggtgcaggtgcatttactctttgtgctctcccaaattcttaatggccctttttttctctttccaaatttatttcattggattatactccttgaataacatcttcaaaccctaat

>CD194497

tgacagcgaacaaggctgcaagagagggaaacatgagacaactgtaagatacaacaaagaaacttgctggaaattaccgtaagccagaaagaccagtgaaaagcaaggaaggcaaagtaatcaccgacattgaagaacaacgaaacaggtgggtagaacacttcaaagaactcttgaatcgaccagctccactgaacccacccaaaatcgaagcagcacccacagacctcccaatcgatgttggcccaccaacaattgaggagatcagcatggccattagacaagcagcaactgccaagatactacacatcctcttcagtaagatttgggacgaagaacaagtaccaatagactggaaaaaaagacttctgatcaag

>CONTIG13236

cagagcatcgtgcagtagctcagtagtaatactgtgacannccaatanaatccagttcgttagagatcaatgtgatatctgaggctgtatctaatngtaatcgacatgaccnanccatannncactanttccacataccttcttttatctgccatacttagagcccgtgtagaatatatactgttaatatatttgttttgaaactggtgaggacgcttcgtctgctttgatctcaaattttctctagtccggcaaactgattctttatgaccaagtttattgcacaccgtgcatctatgatttctaaatctgcaaaatcttgagaagtgccatgcgccacaataccaacatttcgttgaaggtttaggaatttttgtgtgatattgataacttttggcacttttttgtcgttggctcgtcttaacagcatggacagaatgattagatggtgcagcacttttggtttctaacatagccgaatcctgtttcaagtttattaatcgttgacactcaccagtaacagtttgtagcgtcatttcttcgtcatgttccatacgagataataacttcatgcgaacatcagcatcgctgggagaattcagaccagaaataaataataagcatttaaattgatcactggttaacacatttaatttaaatcgttccgcttgtaaatttactcttccggcatacgtagtataatcctcatcagcttctctagtcagttgtagacaattataccggatacgaaatagagacatctgttcacagaacattttatttaaaatgttaacagtttcatcgaaactaatctctcgtggatgtttaggcaggatatggtttttatacttctgatgttcattaggaccaagcttctgtaagagcaaccggactctctttgtgtcatccatagcagcaaattctactcgaaaaatatcttcaattctgccaaaccaggcttcaaaagtgtgaccgctgtctgcatcgaaaataaattctggaattggattaagatatgactctatgcttttacgttctggaatatcttgctgaatagacattttccccaaaagtgcttccaggagttgttgttgacaagctaacatctgttgctgctgctgctgcaatattaactgtagctgttctggagaaattgacatttttttatttttttaaaatgagaataataataataaatccaaaaaaacaccggcaaatattatggattatttaattccaaaatgacgtcaccgactgctgtatttttttccagaatcacgtcccaattcttgtatatttttttttaccgaaatccccgtcgccaatttgttatgatttgacctaggctgtagccaattgttatgacttgaccaaggtcgtcgctgattcgttatgaccttacgaatttaacaaggacgtaattcgcgtaaattatccaccaatagaatacgacttctacgaccttcacactgtgacaatgacgttcgactagtatgagtaacctagcgtccttattggtccttatccgcccaacccgtccacttgagtccaaaacaccaatatcagcctcggcaatacgaatcgtttatttcaagcatacttcgtttatatatcagacagacaggccacatcataccacagaagcgttacgaattcactagtagcgaacggaacaaagacacgtgaccagagaccgataactagctatactgacgcgac

>CF503126

gggtggggttagaaaaggtcgacactaaaaatgcacacctcgccttatcccacggatttccgtctccggcagtaaggactcgtgaagagcagactagagaaaatcaggctggttttcgacctggacgtggtagcatagaccagatattcaccctacgtcaggtcctagaacatagacacaaattcagacgtcccacaatagtagtatttcttgaccttaaggcggcatttta

>CONTIG10685

catcgtcttttactcgaatagcatagcatgcaaagccgaaaatccaccaaactcaatagatacctacctacttatttatttattcatttactaggctacttacttgggcctgttatccttcctgaaggagcatatgctgctaactggcactctccatccaactccgtcctgagaaatgctttccgactctttcccgtgactattcatgataaccatgtctgcttccaattctcgatgcagtgtgttctttagccttcctctatccctttctgtccagcttggaactaatgtgtttcactgattccgaaaaccgtcaagttgtatctcttcatttccgtagctatttcctttgtccactaggacctccgaccgttc

>CONTIG10681

gattcacaaagtgaaaatattttaaatgaactactttatcttatataatataggaacgaattgactgatgaggtaggaaatatttatcaactgaggtggttgttatatgtgttacgtatgcctaaccaccgcctacttcgacagatgacgttacctgatgtagtagtaggctgaaaaaagatacgagcagtctgtccaaaacatgacaccagtccatgaattcatagataattagaacacagatatatgaatggaggatattcttctcagcaaattctctacaataacttacatacccctgttacccctcgtggagtaacataggtcgactacaagcactgtccatccaaccttgtcctgggcaatcctttccagttgctattcatccttttcatgtctgcttccaattctcagcgcagtgtgttctttgaccttcttcttttcacgattccaagttagggactacctcgtgatgcagtttgatgacttcctcaatgtatcctatcctatcccacccgttcctgcgtttattcctaatttcgtcttcacctggaacctggtttgttctctcccataaaacgctgttgctgatggtattcggccaatggatattgaatatcttacgtagacaaatgtttataaatacttgtatcatttcgatgatggtcgtagtagttgaacaagtttcagctccgtacagtagaactgtcttg

>CONTIG5876

ggacgaaactcaactggtcgctatcaaagacccgaacgatttcagaatttccagaaaatctcacagaacgtgttcataacttatctaacttcaatctagacaaggttcacctagaagctctttcaatgggactaaactacaaagtaccaccgaaaagaatcgacaagaatttggtggacgccaatttcgaaaacctctacagccagttctcaaatttgactccaacatcagctgagaatgaggcatggttcaaagccaaattagttgatatatcccagcaatttgcaaacactccaatcaaccaaaaatcttgtctaacgaatgagcacagggaagcattgaaggaacttaagaacaattccacaacaacaatctccaaaccagacatatgctcgctctccaggtaccgagctcgattga

>CONTIG10830

agagaaagccagagtggctcttgtcaatctgggtcatctttgtcgcctttgtgattgtcggatttacaatgcgtcggtgatagttttgctttatgcttacaaaacctgagttgtggatgttagacaaccttctgtgtctagtcaccactgtctccaatggattgctgacattcagtggcaacatgttagtaatgcaaaggttcggcaacgtgtattcggacgcaaaaacgacaattcaattggcgtcacaatcttgaaacaccgaattcggtgtcttgaacatgttctacgaacgccgtaccagagaatcccacatcgcgcattacttgccgacacagggaccggttggaaaaagcggagaggtggtcagtatatgacatggtgtcatggtatgaaagaaagctgaaaaggactagcttctgttggtccttcacgactccctggctagggtccgagagatggtgcaacacagtggctagagaagttatcagatatggctcagagtagaagccgatggcgatctcgctgtaaccttccttaacaagaacaagaccaaatctttaaaatttcgaatttatctaatggtttttaaatcagtcatgttcaatgccttcaatttcagtataaactagatagaacacatttcttagttgagattatgagtcaattgaagctaaaccaccatggaaaacctagaggcactggatgaccgtttcgtcctattgtgggactcctcagcagtgcgcatccacgtctgttgtgagataagaactcactgaagacaattagtgaacggccaacctagggtagttggaaaaccctgactccaaaccaatggtgaatatgggcaacacgatcc

>CD061105

ctacttttccgttctctatttgtttaatagtgttgctgatttttttctgttgttggttagataatgtttaacgggaagtctgtgtatgctgctgcaatgcccagtagctttagtgactctgttctactcaggagttcttcaaagtgtcctacctatcttgttccactgctcttcagcctcagtgattggtgctcctttgtttttttgactggttgctctgatctactatattttttgcaaacatcctggttgtatcacatagttctttcatatctccttatcttgcagcctgtcctactttgattgccaggttttctacatatttccacttgtctattgccatgctcttttttactcgtttgtttgcttccatatattcagaccataa

>CONTIG11125

gtctaaaattggtctcaaggatatcaggtttatgtaccttcggtagtccgtataacctaggtaaatgagtacctgtcggcttaattgattcatatatgtctggtggaattatctgttgatctctgagcttttttaaggatctggtcagttcattttctatcttattatttacatctttttggttattcaatttctgaaattttcggtgatcattcaagatgttattcatcttgtcaatgtagtcatttctattaagtaagacagttcctgttcctttgtcaggttttgtgataatcaggttctcgtcattaataagttcactgatagcctccttatgttttttagtcaaaatacttctgtagttatatctactattcttaaactgatagcaacactcaacg

>CONTIG4765

gataaaattctataggttagttgataataaattcttttttcagacaaaccatttaacttggataattcagtatctctacaaaaaaaacattaatttttttatgcaaacttgctacttgtattagtaagttatttaacttcataataagaatatttatttcatcttactctattcatatatatttatgatacattcttcattattacttacgcctatcaaccctcgtggaggagcataagccgcacaccggtattctccaactaacctagtcctgggagatgctttccagttctctccagttgttattcatccttttcatatctgcttcaatctcctggcgtaatgtgctattcggccttccacttttccgcttcccttcaccattccaagttaaagattgcctcgtggttctgtttggtgatttcctcaatgtatgtcctatccacttccaacgtcttttcctaatttcctcttcagctggaagctggtttgtcctctcccataaaatgctgttgctgatagtatccggtcagtgaatgttggatattttgcataaacaactgtttataaatatttgtaccttcttgacgatggatgtcgtagctctccacgtttcaactccgtacagtaacaatatcttggcgttcgtattgaagattctcactttgatattggttgacagtcgttttgagtcccatatgttcttcaattgtaggaatgttgtccttgctttgccaatcctcgccttcacatctgcatcagatccaccgcgttcatcaatgatgcttctcaattacgtgaaagtttccacatcttccagagtttcttctcatttccgttgctatttgactggtcttcccggtttcccacactgtccggacgtttcacgtacctataaagagtgttgctctggttgttggaagggacattggcctcgtgacttccgaagaatcttggcttccatcatgaggcgtcatatttctgccctcaactcccagggtagagtttaaatgatttactttgtttaatctggttggcgattttttcctacgggatgtggttgccaaccccatgtacaaccctcctcctttacaagggcttgagaccggcagcaactctagaagagctacaggcggagtttcatgcagccatggtgccggaaaattgtgtacgaataagtcagtatagttaactttgaatgcaacaggtaactacaaaactgattaaccgtgtttaaactcagacactttaaaaatggtaacctcgatcaacattagtttagatctaggtgtcatcgagttatttactgtgaaca

>CD085444

tgaacagactcgaagatttaatttcaccattgatcaagacaatacttcagacccgctatttcgatgtggatagcatgctcattaccatcaaacggaatgtgctgaaaagtacccacaactccgttaataacgtcttccattacttaaaattcacaatggaaatgcaggtgaacaactagctaaccttcctccacgttctgatcaccagaacaaatacaggaggactgaaaaatctagtgtttcgcaatacaacaaaaacaaaccaaactctcgactacacttgtaacaaaccaaacacttacatgatcaactacgttcaaaccctattcaatagtgtgaagatacactgtagtacaactgcctaccatacatacaaaggaaaagt

>CD167839

catacactacggaaatcatcaaaacgcattacgagacaagccccaacttgggatcccgaagggaaaatgaggtgaggaagaccaaggaacacattgcgtcaggaattggaagtaaaaatgaaaaggatgaatagtaactgagaacaactggtggattgttcaggatagttggatggagaatgatggtgggcggcccatgctcccctacgaggagcaacaggcgtaagtaagactgtcaataaaatgacaaacgcgaaatagactaaattagagttgggattccgattggtgacttggcgcgtgatatcagtcagcggccccaagacagtacttattattacaatcgttttaccttgcaagtagctgataaactcctaaaatgaaaacatccctcttttaatcaaccggaatcgctctttggcaataatttacaaaagagctttaagtgattgccttaatatgatactaacccctataacaacctgacgcctgtctagatatatatgaacgtcgaaccaagaaagttccaagtagtttcagacctgat

>CONTIG3877

tatgtatttctttataaccccttactgtaatttattgaagttattaacttgacttaatgaaattaaagtgaagattcctttcttttaaaactatcgctacatctataaaatattttttaatgaagcatatccttacttacttatttacgcctgttactcccaatggagcataggccgccgaccagcattctccaatctactctgtcctgggccttcctttctagttctacccaattgttgttcattcttctcatgtctgtctccatttgtcggtgtaatgtgctctttgatcttcctcttttcctttggccttgaggattccatgtgagggcttgcatataactaactctaatttctgaagtgtttttcttaaatcatactattcaatattagcatatcgctttataatgaggtataagttgagatactgcatttgattcagtacatattccaaaaatgttcataaagtatgaaatttacattaacagggtcggaattatgagtcacgtcttcattgttaaagttgtgcattatattcaggacttctagtctttttcccgt

>CD062022

ccgcaccacttagaaagggaacagaaacgggaagagagtaagcagagttgcatgataaatatcagttgacctccattcacaacataccagtgtattccgtcttatgaattccgactgatatcgacgatcttctcaggtacaccataatgtcgaagaacgttacatcaggttcttttctacacaatgtcaaacaccttatcatagtcaaggaaagagatgattactgacgaattccactaaatcaattcataatcattggtcagtaatgtcacattatgatccatacacaaccaatcctcacggattccagtctgttgatctcgatgttggcaatctactgaatctcatcttgttctacaacactctgttagggagttttccgaatactgacagtaccg

>CONTIG12369

gaaaggtcggaagactagacgttatctgcgtgntgcttccgatgggtctatagcccataataataataatattatnnatgtccatatnnatcatacagttgtttatcaatacacttacttactcttgttactcttcgtgggatatagggcaccgacttggattctccaaccaactctctctcctgtgctctactttccagttgtttccaagctctattcattcttttgatgttttgcctccagttcccagctcaatgttatttgttatacctcttctcctttacccttcagaattccaagtcaaggtttttcttgtgatacagcttgataatttccgcagtgtatatcagatcaacctccagcatcctttcctaatttcctcttcagctagaatctggtttgttctctaccacagtaggtcgttactgataatatccagctaacggttctgcattatcgtgcgtagacaattattcataaatacttgtatttatttgatgatggttataatagttctccaagttttagctccatacagttgaactgttttgacgtgtatgttaaagatcctgactgtacgcaaatccatatattttattttgaattatactgactgaaaaataggtcaaaagtgttatatctgaagcgtttcgaattcactagttagcgaacggaacaaagacttgtgaccagagaccgataagctagctattctgacgcgtcccagacccagctaactagagtaagaagtccaagttggaagcggagaaaatatattccgtagcagccagaagcacagcaatcgtaaggggaaaaaatcaaacatatatatacagcatgaaattgtccttgtgaagcattacaaaatagcacactaaaaaaatacaacggactaatagcgtttaagatatttccgagtgggaaatacgactcgaaggtgaaaggagcgcttttggcgcgaaaacaaaggaattcaaattttcaaaataaaattacaaaaataaatcatttactaagtgagttctagggatttaacatccccaacaaatagtgattttaaaatttattttaatatgtgccattaaaaaaattgaacttgttcacatcattcaaatgaccaactatcataggaatagattgtaacttctaacgcttttttataattccctattttaacattttaagaaatccccatgacaaacgttttgtttgtattcgattttctgtgcctcgattcctttttcttagaatattaaaatgactatttttattattctgtaatatctttaaactagtaagcagactgattgattcatatgcatacaacattgaatactttttatatcttcaaagtttattagatttcgcttgtttgatctggttacttgaatatggaaacctcttacgactagttcattaagatctatcttatcaataaccatttgaccaaaaacacggatggccaacctcaaggttttatcaatccaacgaggttcccttattagtggaatacaataggccaatattccttccattacttcatagtcctgattcttatccttagtcaaaaacaaaggtttatacatatcaattacctgatcataatttttacataactatgttaacttactataaattaatcacaataagccctcgtgccgaatnttggctcgaggacggac

>CONTIG1389

gatataacttggcggttctcgggatcagcgaaacagattggatgtgtccaaagaaccacgtatagcacttataagataggagtctcatgaactcaagatcatcaaagcatctttcaaaacaaaggggggggatcataatgaatattatccaatgttatttgcccactaatgttagcaatgacgacgatagagattagttctacgagaggctgctaatcaatcacagcgaagtgtccaagaaagaatctcaccaacatgatgggatacctaaacgccaaagtcggaatgggaaacaccggttgtggagatatcatgggaggacatggactaaataggagaaagggacgagaatagtgacagatttgtaaatttatgtgcattcaactaaatggttatagacggcataatactcccacacaaacgcatacacaaatctacacgggttttaccagatcacacttcacataaaccagatcgaatatacttgaaccactgaaaaattcagaaagacaatggaaaacatgagagccaagacaggggctgagatagcttcagatcaccacctggaggtcatcaagatgaaaacggagctaaagaaacactgaacaaactggaaaaacagcattacaaaggttcactacaaccttacctagaaatactagaaaaccc

>SMLC53F03

aattatttcaaacaaattgtttacgtattatttcatattcattcatagtttaacattgattcataactgaaaatttatggataatttaaactagaaagaggtcaatgaagaaaaataattccttccatatataatcataaacaaatagaataacaattgtttctgttcaatcaacagtgaatatccgaactcgtgataagaaaaaatgctggggcgtgtgaggataatctatttggttgaactaagattttggttttttcaaatttttcagcatcacaagtgttgatccacatgtcggagatatattccaaccatttcaaatgattattcaataaaattttgtacataatttctagtttgtaaattattatttaacttcaatcttattacttatcataactgcatatataacaatgaaattacaatctgtacagaatacttctagaacaattctcaaggggaaataggacttctgttcattaacacatggatatccacaaccttgagattttgtttgatcagaacaactatgcaagacgcttggaacttttatggacttcacagatgacctggtccttctatcccatacacgctaacaggtgcagatgaaggcaactagtgtagcatcagcctctgcatcagtaggcctcaacatacacaagggataaaagcaagattctcaaatacaacatggagaacatcaacacagtcacacttgatggagaaactctggaagatgtagaaacattcacgtacctgggaaacatcatcgatgaaacc

>CF501162

ttcaattggtgttactgtttaggattaagtcgagtaaaacaagtacccttggctaagaccctaaagtcttataccattgatgtatgctgtgtctccgaaacacacatacaggatcctagtgtggtcattcactcgacgacgagatacaccccccgtttatctggtgacccgatggtcaattctcgtggactggatgtaggcatatcactaattatgagggcagaacaagcactatcagaatggactactgttaacagtcgcttatatgctattcgggcaaacagctccttaagaactcggaaggatagggacacacgtcgttgcctcttcgtcgtttctatctaccctcccac

>CONTIG12031

ttttcctcgacccgagaccatattccgaatgcggcctcacaaaatttgtttggaagcgaaatgaaatacttgattcccaaaatctaattgcatgaacctattttggctgtttaaccataaaattttactaatacgtcttaaatatttgcaggtatattggagattatggttaaatgaattttggtcgtatatcagtttaaaatgctgaagtatattttacggtttacgaggttttatcgttatcgtgttcacaagtatgtcgaagtttaggttattaatggaactttctgtctctaccttcagccaaaaatttattttgttagaattagcacctaagtggttgcatatcattcttaaaatttctttatacttttgtaaatgcataaatacattcaagtgaactcccgtttttgtagttccatataaataaagatggctgctaagacattgctactaaaatcaattcatttgttcgtgttatttatttatttatttgaacacaaatattggtacaaagaggcactgaatacatatgcaccacacgagtcacttgatttgtttgagggttgtgatactgcccagatgcccacactgtagcaagtggttttcttaggggaccacacccggagccttcgacctaaaggtttgatttacaaggcagtggagcatcataaggagatgcagtccaatggtagccggtgaccagcaactgattgatataccatttgttccctcaggatactggagcccatgtgcaccactagactggaatcagggttttccaacgccactaggtgaaccctccatatccgccaacccgagtggagtgccggacagtcgctttccgtcctctcaattctgtaaacaacacccccgccacgagaaggtggtgagtaggacttccctggcagaggctatatacgcgtagccatgtgagagcatttcgagagggagggcggactctccccactctcggccgtaccagggcatttgggttgtatataaggatatagtatagggaggaagaaagatataaagcaattttaatctcaagctttaagagaaaataaagagtatatacacccacgccattgtgatcgattctgagccatgtcacacacagtctccaaacattagttacgatagtcacacggaccccaaacaagtagtctgcaatctaccaacatggctcaaaccagaagttagtgacttcaagcactgatgccacgttttggtttggctgcccctaacttacctgacttaccatcaatctctaatactctacgtctatcctcattattacttacctgctgattacagcagatacgagcgaaattcctaagacatttgtggtcaaatactagtaacttacgggtattctctactcttaatggccacgcttcgtagccgtaaagtagaacagagcgaactgttgcgcagtatactcgt

>CD095657

aattaggaaaacacgttggcaattgataggacatacattaaggaaatcgtcgaactgaatcacgatgcaatccctaacttggaatccggaagggaagcggaaaagaggaaggcctaagaacacagatatgaaaaggatgagtggagactggaaagaactggaagggaatgcccagggcagtgttggatggagaatgctggtctatggcctatgctcctcgacgaggggtaacaagccacaatatcaataaggagaagttataaacccttaatattaattctatctgtcctacaaagtttattctttcttcattaatttttcatatccattagagattacacgtaaatatctaaccgaagaaatattagctgcgacaatatttgttgaagcagtttatcctactctacgaattactgatattcatggttcaggaagtttaaataatactagtccagtggaactttggcgaagtttggatatagacaggtttgtctttattgttcattaatataaattttatcatgacgagtattttaaattgtcttttggtacagcagaatgaagttaccaaaatanaacgaagaggatttagattaagaaatta

>CD132681

ctgtactacttgaaataattttcctacttttggttttataccagtagttgttttgttatttgcatttctaattgcgcttatgggaatcttcttcatgtaaaccgataagtatttgttctagactctctcttggttgtttctgtgtgccagaaatatcaaacatccttgcaagtaaagctaccttgtatctatactttttacacaatatattccaattcgttttacagcatatgacactgtggccacagagacatttgtacattcatatcaatatccttttatcagcaacaagttactgtgggagacaacaaaccagattccagtggaggaagaaatcaggaagaagcgctggaagtggataagacacacattgaggagatcacccacaagacaagccctcacatggaatcctgaaggccaaaggagaagaggaagaccaaagaacacataccgagaaatggagacagacatgagaagaataaacaaaaactggata

>CF502072

tccacgagaatggtgagagatttgcaaatttatgtgcattcaacaaattggttataggcagaacaatattcccacgtaaacacacacaagaagctacatgggtctcaccggaccacgcctcggaggacctgatagatcatatttcagtcagtaatatgttcaggtcaatggaagacttgagaactaggaaaggagctggtatagtttcagaccaccacctggttgtggccaagatgaaactgaagctaaagaaacac

>CD091606

ttgctgatggtatccggtcaatgaatgttaagtattttgtgaagacaactatttataaatactcttaccttcttgagggtggttgtggtagtcctccacgtttcagatccatgcagtaggactgtattgacgttcatattgaagactctcactttgatattgatttccgaacagttgttttgagtcccatacgttctttaattgtagaaatgctgcccttgctttgctaatcctcgcatttacatctgcatccgatccaccttgttcatcaatgatgcttctcaggtacgtgaatatttctacatcttccagagttaatacatcaaatgtgattgtgttggtgttctgcgtgctgtatgtacttgaggatcttgcttttttcgtcttgtatgttgatgtctactgctacagagattggtgctacattagttgtcttcacctgtatttgttggtgcgtatgagatagaagggtcacgtcatctgcgaagtccaaatcgtcttattgattttgaaatgttcattgtattccgtgcttcccctcagatgtcaaagtcttcacaac

>CD117910

ggacatctgtttactcaggaaagcaacaaccaacaacagccgagtaagagcagataaagccaaggcacagaccgaatacacggaagcaaacaaacgagtgaacaggagcattggagctgataaacggaaatacgtggaaggcctagcaatgacgatgaaaaaagcttcaagaaaaggaaatagaagacaactgtaagatacggcgaataatttggcgagaaaacacagtaaatcagggcgaacagtcagatacaaaaaaggctagtcattcattgcgattcaagaac

>CONTIG12596

gccagtgtgctggaaatgcgccattgatgaacgaggatctgatgcagacgtaaaggtaaagattagcgaagataggggagcattcctgcagttggacaacatatgggattcaaaacaactgtcaactaacatcaaattcacaatcttcaataagaacttcaagacggttctattgtgtggagctgaagtgtgcagaactaccacaaccaccatcaaaagagtacaagtgtttataaacaaatgtctattggtcgggtacaatcagcaacagcctattgtggaagagaacaaatcaacttccaggaggaaattaggaaaaggtgttggaagtggataggacacacgttgaggaaatcttcaaacagcatcatgaagcaagcgctatcttgaaatcctgaagggaaacggaagagatgaagacaaagaccacaatgagccgagaattggaagtagacatgaaaaggataaacagcaactggaaaagactggaaagaattgcccaggacagagttagatagagaatgccggtgggtggcatatgctcctctacgaggggtaacaggcataagtaattacattgaagcagaagattcaagaaaaaattacgtagtttttttcacaagttatttaggtaggatgaggtagtagtgagaaaaaaaatatctttttgatgtttatttcaaccacaaaactattaaaacaaaaaaaatctagttagttattataagactaagatacgaaccagatgacttcagaaccaaggtttctaagcattcgtaggaatttctttattcgatcacgaattgtagcaattttttcctaaaagataataataatgagtagcaaaaacctaatagattttacatacagttatcttgtagttcacatatcacaaaagtcaggagaacgatcaaacttgaactagaatttaatcatatatcgataaaacaaagcgaaaataaagtttctaaaaaaacaaagggaataaaaataaactcatagactgaaataatagaatagctaaccatagatgggaaacaatgcgtttgtacaactgtgttccaacttatctgatcacatttcttacaccagactatttttctctccacttgtaaacaatagtaagcgttttaattgactgactcgcgacatcattaacgaaatcaaataagacgtgtagaaatatacagttcggcgaaccagcacatactac

>CONTIG6095

atgggaataatgttaacgttaacgaagacttgattacatgaaatgtagttcaacagagaacgtaaaataaaaagtatgaagagataaacttaatattttgaaaaagatgaatactcagtgtatttgtgctactgcaattaatttcggagaatgtcacttaatagttctaactataaataaagatgatcccataaattcaaacttgaatgtctgcatcgaacgacatagatcagtacagtaatcattgactttacaaagtaaatcatgtctattttaattacttacttacttactcctgttacccctcatggaggagcataggccgctcaccagcattctccatccaactctgtcctgggcaatcctttccagttctgttctgttctgaataatgctttgattatttcggatccatgagattcccattctacaagttcatttcgtgcttctctggacagcattagagcgactccctgagtgtgtggagcattttcccc

>CD132327

aatctttaattcggttcaacaacatttggttgtaaactcttcttggtactaatagtagtatgattcctctgtttttttcgcacttgctgagatctttctttggtatcttgatgagatatccttctttcccgtctgccggcacttctccccccccctcccaaagctttctgaatagaacttggagtatgtttgcagttacttctatgtatgacttcattgctttggctggtttgttgtctagtgctgctttcccacttttggtttgtcagatgactggccatcctgatttcttctgtcgttgttgagataagatgtattggaatgtctgtttgtgctgcttcgaagtctagtggggctagttttatcaggagt

>CONTIG7048

cacgcacctggacagcatcgccgatgaacaagaagggtctgatgcagacgtgaagatgaggtttggcaaagcaagggcagcattcctacaatcgaggattacctggaattaaaaacaaccgtcacccgagaccgaagccataatcttcactatgaacgtcgagaaatttctattgtgcgtagttgatacttagagaactactacaaccaaaatgaaaggtgtacaggtattcataaacagctatctacgcagcatactcaatgtttgttgaccgcatactatcagcagcagtgtactgggagagagaacaaagcagcttccaactgaatagggaatcaggagaagacgctgcaggtggatatgtcacattgcagaattcatcaaactgaatctcgaggcaagctctaacttggaattttgaagggaaaagaaaaagaggaagaccaaggaacacattgcgtcggaaatagtaagcagacattaaaagaatgaatggcagctggaagggattgtccaggacgtagttcgattgagaatgctagttggcggcttatgctgccgcacgaggtttaacgggcgtaaggaagttataattttgtatgtgtgattattcaaaaagtcaaaagagcttctattttttcataaccagcctttgtataactttttcaatctgtgttaacttattgctacattcaaatacctactatgattcatattgatcaatgatttcattgctctttgtctctaaattgtctgcatttatataccccgtttgtgtgtcttgtagctttcctttcactcctgatatgattggatctgcgctatctttatcactaattggtcattcacaagtaagttggaaattaaatctgtcatccattatctatatgatggaagttcagttcagttactttgttgaaatttatcgagggagtttctca

>CD094437

gaaattttaatgcacctgatactagttggacagaaatagccactgtaggatctgaaaactttcgatagtagcctcttgatgacgtaaatggagcattccttagtatagcatgtctccaaagccacacgtcttggtccaaaccaaggatatcccttgttagacttagtaaccactcatgcgactgaggttatcgcctatctaaacatcctctcaccgttagctaatagcgattatgccgttttgttattaatgttttagggcttttgacttaatacactatcaggttagacctcgcctaaatgtgttgtgagcaagcacaacagccatacaagagtgtgctgcaacaacagactggtcagtaaata

>CONTIG12968

gccagtgtgctggaaagcttggagttcacccttactctttgttggcgcccaactaggaagtcttttatccacatcaatagattgcctccaatcccgatattccttagcttatataacagccggttgtgcggaactttgtcaaaagctttgccgaaatcgatgtaagctacgtctataggtaacttttgatccttaagagagcaccagctttcacaagcgactagtaagtttgtgagacaagagtaacctgttctaaaaaccatgctgcttttccgagaggaaccggttttcatcgagatacttaaacagctccttccgaataatcttttctaagattttaacaaccacactagttaggctaatgggtcggtaattttcaggcttatgttttgtacctgttttaaagacttacattatttttcttcctcacattacgcaataatatatcagtccagatgatagcggtgatacttatcatggatagaactgtttacgactgtatattaatggttcatatcgtaatgtagcacgttatatattgacgtttacttggaagcacgagcgatctgaaacaacaacactacagtcagaaccggtaagtgccgttaaagttagaacacagtgtactggcacaacagatatatttttaaaactgaaagacacggttcgagtacaaaaggggaaacttactacaaaatagttttattcattgttctgctcgttcagatcgcgtagggcttcttcatcattttccagtgcgtttactctttgttgccctgcaatagaaaaacaaactcgcattagaaacgtcgcatgaaacgattattataaccattacaaggttcatgaatagtttgacacttaattgcttcaattacaatttataaacaactaccaacacctatgttcatcactaccataataggggaggcgagtcatatgtaaattattttacagcttgtttttgcctgaaactatagtttcggtccaatttttgactcgataaatgacgaatttcactcacctgagtttgtattgttacaaattaccaaataaggctgactaaacattcattcaggttgtcagctcaaaatacgtcaggcaaccaatagactgaaataaatgccatgtaacacgcataaatattacaaaaattaacatgtgacggaaaaatgctgtgattagccgataaaaaacgacattaaaaatccgcgacaacatgtaacgtatgagcgagaatcaatgacatgacttttcgaccatgccatcncaaacggagccttgtgtttgcgagatttcgcatcccaattggagtttaacaattctaagcctttttaagcatgaataaacaatataagtatcaagccgatgatcgaattcgtcagcaacaggctaaagttcaggaagttgttggtataatgaaagacaatgtgaatctcgtattggaaagggaaactcgtttagcagaaattgacactcgagctgatgaattacaagttcaatccaaacagtttcaggcagttgctggtcgtgtacgaagaaaatacttttggcaaaatatgaaggtaaattgatagggagaagaaaaaccaaaagttgtgttagccataagttaattgtctgctgaagctttctaattttttaccattttaatagaatgttccaaaccgaaatgtatcattagattataaacattttctatttgtctcaaaaaaagtcagtcagttagttacaacgtagaacttcgtacgtacgtacatcagttcgagttgccataccacattagcacagagatccagttgtcgattcaaatcccataatggtagaagtagtaagagtattaagaattgaagagataatgttttatataagatgatttacaaataaagatatatgcctttgatgataaatcaaaacagattttattctagttctcgttgttgaaaaaagtaatgatagaagctataaagaaaagcaaaaggaataactgcctatcactgaatgtatttccttttaatcattatgatcttctagttgtgaatgataatcagaattattctccgataatgaactaatcatagattgtgtactcataaaattagctatactatgcctttttgaaaatttaataaaactggtgcaacaaattgttctttattattaattcttacaggatgttctttacaagtatttatatcatttctattaaatacttgtaaagaacatgtaggaggggtaagaagaggtgatggaggtgataataatggtgaaggaggaacagaagtggttaaatttttagcttgtgatatatctattatcgattgttgagtaaactgattcggatcactattatttctgtgcataggaaaagtagcatcgaccactgattttcgatcaccaactgcattggttatatcgttcaaatgtgatcctctatgttgaacactgattggactattatactgactatgttgatcaacagatgaatcgtcagtggcagagactaaatgtgcatttctaaatagaagtttacgtgctgaactactaccagccattatccatccagtaataatagtgaaaatcattaatattgcagagaatattaaacccaataatgaacaaattactaaagcatgactgacagtattggcctcgtgccgaattnttggctcgagggccaata

>CD149639

ggacactcgaaggaggtttaaaaaccgagatcaaatcgtctacttccggagttttgtttctcgcactgagtagtgaattactggaattacctacaagaacatatcccattacctactgttgatatattcaaaaccagattggacctccacagtgttacaaactgcaaggaataatataggtcgatggacctcctatccttcttagtgaggactgaaattgtatatacttctttccccagcggattgtgtcgaaaatttggcacacactgcttgagtagttaatgtcattcatcccctaaactagagggtctagtgaaggtcctgcggatattatgacacttggcagtgtagctgagaatcttttccaacattttaacattgataccaaacagtggtcatactcacgcataactgtcgcgttattagctcatgaatctttgggattggtttagctgtgggtttctggataattgctataaaaagcaaccaattaggta

>CONTIG10887

ttaacatgttacactctgatgaaaaaatcaagttcaccaaggagttggaatctcctgaacacaaacttaccttttctagactgtttggttgaaaggaaatataatgattgtttaaaatttagtattttccgaaagccgacatattccgcgaaacttcttgattttaagtcggtatatccacactcgactaaagtgacagtggccaaaaaaatgaccacaagagctcaaaaacttgtcactgaaccaaatgatatagaaatgaaaatgaatttaactacatccaatctaatgatgaacaattatccgaaggattttataaaaataattaagaacggaaaacaaaacgatattattaaaaaaagagttaagaagaaagaatgggttaacacagtggtgatcgcatatcgtaaaggtatttcagaagagatcagaaagatcctaactattcaaaatataagagtattcttttgaacaaacaatactttaatatcaaaactaatgagaatcaaagatccaatacactaggagggacaacaaaattgtgtctatgaaatcaaatgtagtgactgtaatgcaaaatatgtagatgaaacatcaaggaacacaaactatgcttaaagcacattcctgagtcctcagatgatgtatgaaaacttgagagcagtcagtgctagcatt

>CD152740

atcgagatatcaagcagacgacatagaggacaaagaaacactctcaaggaaaaaaacgaatcaagatttcaaagccacccatagaagaaacacaaacaactgtgtggtcattcttgcaaaacaacctctgataaagaaagaagaaaatctccgaaaagcatttaactacaatacacgtgacgtttcaaaaaactagaattctccgccgcattagagtcatcactcaaaactgtcgggatacaggaaaaaactcagtaggaattaagacaaaccatagtgccactaactcgacagaagacaaaacccaaatgaccgcacaagaacagacagttttaaagaaactcagaactagagaagatattaccatagttccagtgcacaaaaacgcgctacggtagttatgaacaaggaacaatatatcaggaaagcaaagaactgcttgaggatgaaaggatatac

>CF495603

ttaattcaaatatagtgaaggctagagcaattcatactgatctaggccatttttggtgccttcatgatgctagtttggatgtaaaaggttggatatataacgcgctgatgagagcagtttagctgtatgtttgtcaaacctgccccctccagttgagggtgtgaagtgactatctgcgtttgattaccgttgtcccctaaaggactgctgatattcagtagtgacaccgtgttattagtgcgtaggttcggtatcgtgtgttcgaacacagtgacgataattcaattggtgccaccatcttagaatcccgactttggtggcttggaaatattctacga

>CONTIG10909

atacgcagaagcaaacaagcacgtggagaagatcattagagctgacaaacgaaaatacgtggaagacttagcaataacagaggaaaaagctgcaaaggaaggaaatatgagacaactagcaggggaaatatagtaaaccagagagaccggtcaaggacaaagaaggcaagccaatcactgggattcaagaacagaggaacagaccttcctgtagacgtaacttcacaaacgacggaggtaatcaaggtggacaattcaagattgggaaagcagcaggacctgacaatataccagcttaagcactgaagtcatacataaaacaactgcaaacatgctccacgttttattcaggaatatttcagagagaagaacaaatgccactgacagactggaaagaaggaaacctcatcaagataccaaagaaagaagatctaagcaaatgtgagaactacaaatacatcacattactgtcagtacaaggaaaagtttacaacagagtgttgctgaaccggatgaaagactcagtagatgcccaacttcgagatcaacagagtggattccgtaaggatcggtcgtgtacagaccgaatcacgacattacggatcttcgttgaacaatcagttggatgtaactcatcaccatacatcaacttccctggctatgacaaagcgtttgacagtgtcggtaggaggatcttatgaactcttcttccacactatggtgtgcctcagtagatcgttcacattatatggaattcgtacgacggaccacactgcgaagtggtgcacagatgacagctgacagatgcatttcgagtgaggaccggcttcctaacatcgtcttttctctttcttctcgtggtcaactgaattatgaagagctaaacgcctcgtgntatactattagggagagcaanntacctgggaagtttnatgacaaatatatgatcactgaggnntatataggcgaaataccacgggtgnacagaaatccttgntcatgctccctacat

>CONTIG10995

gatgcccattctccataggcagtttgctaatttctttaaattcatcgcagcacaaacaagcatcgcctgcatctggtttctttccaatccacggtaattcgtccagcgcagaccatgcttttctttcaaatctgcaaaatttcgttcgattgtctcttttcttcgttcatacaactcccgatgctccggcacatatcgtaagtgctcggcttcctcgtagtaatcttgccaaacatggcgtgtgaccactttcgtgtggcttttgctttcggtgcatttttctagataaggacacgtcttgcatacagatggatttgatttatattctcgat

>CD062747

gagattgatatgagaggaatgaacaaaagttggatacaactagaaaggaaggttcaggacaaagtaggttggagaacgctggtcggcggcctatgctccatgggggcttacagacgtaactaataactaactaacttgtttaaactcaacaatttccatatttgcgtgctcttggacctcacttcggttgttgataaagtgttagaaacattagccccagatactgtaataaacgataacacacagagacaaccaactgtgtcttactataacattacagaatgtcttggtaaaatctgagaaccatacagtaaagacttcgtttgcaaggtatccatcgattgtctcacattatgttgttccttagtttccataacaatcactctactctttgttcttgatctcttcgattttctcaaccttctgtccccatg

>CF500489 (CL313)

gaagacttgaagtttagaccctgactttaaattttctggctttctactcctccatgaaataatgttgggtcaggctttctggtacttctgaagctattgggcttctttttttgacaaaatccctctttattgcccacagctttacacttcctgcaccgatgttgtttataaggacgccttcgaacgtaatgccatgcaccgcggtgccatcatggagcaggagggtaaggtcatataagtcgttgttctgattggctaccttgtcacataatatttgatgagccacaggacataacatcttccatttatgaaaccaatccttcaacagttatcattaacgaaaatcgtggaaaaaggcataaaacacgaagaatacgaatgctatacaaaaactaagtgtaagtagacaatcaccaaacaaaggataaaaataactaanaaaaggggcggncgc

>CD127317 (CL982)

cctgggctcgaatctcgcgaggcgggatcatggatgcgcactgctgggaagttccacaatagaacgaaacggccgtccagtgcttccaggttttccatggtggtctagcttcaattgacttatgatttcaactataaaaaaagcattattgagaaagtcattaatcagaatttattagcacatagtattagttaaaaagtacaaagaccaattctctttccttacaaacgcattaaagcatctgaaaatgcagataaaccatatatcctgtataatggcacatttataaacgactctgaaacagttgtaaaactctccagtggtattttttgagaacaataacgaattcctagatgatactggctaataaattgtaactccataaagcttttttttcagtaatctaaatattagtaaagctataaactcccataaggcctcagacagtcatgctgtagatggcatttcacccattctctataactatgattgatctcatgttccactacttcatctaaagctgtttcaacacacacacgcacactctctctttcttcctcactctggagacaggtacttacccctaatgatgaaaaccgcgcacatcataacatgttacaaatctttagataacgctgagatgaacaattatcggtctattaatactattctagttatctctagaataatgaaaaaattactggtgtcgaat

>SMLC54G08

tttttttttgcatgatatactccatttaattaacattttattggaataaagaaaattataaatatgtacattaaaatatcgaaatgaatatatatactactgaaaaatactcaatttaattccaataacataagcctcatatgtacatgttacaatcatagttcatttgaaataataaaatatttatgtatttcgataattcagcaacaagttactgtgggagacaacaaaccagattccagcggaggaagaaatcaggaagaagcgctggaagtggattgggcacaccttgaggaaatcacttaattgtgtcacaagacaagccctcacatggaatcctgaaggtcaaaggagaagaggaagaccaaagaacacattacaccgcaaaatagagacagacatgtgaagaatgaacaaaaaaatggatagaactagaaatgaaggcccaggacagagtgggttggagaatgttggtcggcggcctatgctccattgggagtaacaggcgtaagtaagtaagtaagtatttcgatcatgtaataaggagagacgaagattatcagaagacctatgtgatgagatattggcgtaatacatttcgaacaatctgatcacacataatatacttgtgaagaacactcntatataaaaaaaataaaaagtcaactagactggaaatatggggggaaagggtaaggggagaccaggataaaaatataatatgaaaacattttgacccctatcactctagataatgagctaatatgacatcaaggataantttaaggattatacctaatttattagtacccttccctcctaataaatattt

>CF499612

gaaatggtcaaatagagaaactgaacagtaccctttggaaagctataaccttagctttacactcccaggacttgagtacgtctcagtgggaactagtattgcaagacgcgttgcactcaattcggtcacttctgtgtacggcgacgaataaagctccacatgagagactctttggatactatcgcagaagtacttctggaacatccctaccaacgtggttaacatctcctggccccgtgttgttaaaaagaaataatagatcctctgagtatgaccctttaacagaggaggtagagctggtgaacagcaaccctcaatacgctcctatcaaaacatctacgggccgagaagaaacagtttctttaagattgttggcgcccaaagaaaaagactgtttcacagagaatgtgagaacccctgtgctgaga

>CONTIG8929

tatgaattttcactttcgtgatgtttcgaccaaaacttctaatcaaacgctgattggttaaaatgcttcttaatatttcctgagcatttcttgtctattacgagaaattttcaagatcggcccaacaggggcataggccacccaccagcattctccattgaactctgttctgaacaattgtttccggttgctattcattctttcaatgtctgcttccgattccaggttcagtgtgttcttcgctgttcctcttttccgtttccctccaggatcctaagttagcacttgtctcacgatacagtttgatgattaccacaacgtatgtcctatccacttccatcgtcttttcctaatttcctcttcagctgaaagctggtttgtcctctcccacagtagtctgttgttgatggtatccggacaacggatattgagtatctcacgtagacaatggtttagaaatacttgtgatagttatccaagtttcagctccgtacagtagaactgtctggacgttcgtattgaagattctcattctgatattggtcgacagacagttattttgagttctagacgtttttcaagtgtaggaatgcagttgttgctttgccaatcctcacctttacgactacattagatcctccttattcatcgatgatgctgttcaggtaggtaggtgagtttccacattttccaggtcatctccatcaagtgtgattgagttggtatcatctgtgttgcatttgaggcctactgatgcagaggctgttgctacactagttatcttcatctgcatttgttggtatgtatgggatagaagagctaggtagatgatttacgaagtccanatcgtccagttgcatccatgttgtccagtgtattccgtgcttcccctc

>gi|2636685|gb|U66335.1|SMU66335 Schistosoma mansoni SR1 non-LTR retrotransposon, internal fragment H1, pol pseudogene

AAGCTTAGGCGATTGGGTATAAAACTCCCTTTGATTGATTGGCCCTCTTCATATTCCGAAAACCGACACT

TCAAGGTCAGGGTTAACTTCACTCTCTCTCAGGCTATGGAATGTCCTAGTGGGGTCCCCCAGGGCTCAAT

ACTAGGACCTCTTCTCTTCTTGATTTACATTAACGATCTTCCTCAACAAGTCTCATCTGACTTATTGCTT

TGTGCTGGTGATGTGAAACTTTGGAGAGAGATACGTAATCATAATGATATACTAGTTCTTCAGGAGGATC

TGACCCGACTTCAAAGTTGGGCAGACGACAACGGACTTACCTTCAACACTTCAAAGTGCAAAGTAGTCCA

TCTGAGACATGTTGCAGACCATAGTTATAACTTAGGTAACTCCCCTCTAGAAGTTTCCCAAGTCGAATAA

GATTTAGGAGTGTTGGTACCCTATGACCTGAAATCGTATGCGAACTGTGACAAAAACGCCTCTCAAGCAA

ACCTTGCACTGGTAACATTGAAGCGCATTTTTGGCCAGTTTGACGGTAGAACCTTCCACATAATCTTCAA

CAGTTTTATTCGTCCCCATTTAGAGTACGGAAACATAGTATTTCCTCCCTCCCTCCAAAAGGATAAGGAC

ACTCTGGAACGTATACAACGTCGAGCCACGAAATCAGTTCGGCGACTCAAATTCAAACCTTACGAAGAGC

GCCTCCAATCACTTAACCTTTACCCGTTAGAGTACAGGCGTCTTAGAGGCGACCTTCTTATGACTTACAG

TATCCTTGATACTTCTGGTCATCCCCTTAAACATCTTCTTAAACTTAGTCATAACACTAACCTAAGAGGT

AACACCCAGAAATTGGAGACCCTATATAGCAGAACAGACTGCAGACACAACTTCTACTCCGTTAGAGTTG

TCAAGTGCTGGAATTCGCTGCCGACTGAGCTAGTCCAAGCGACCTCCCAGGAGTCCTTTAAGAGGAAACT

TGACTTATTCTTAAGGACTAAGGATAACATATTATGATTTACCAAATTCTTTTTTTGCCTCTATTATCGT

TCATGTACCTAGGTTTTTGCCTGGAGGTATTGGCGATCCACTGCTACTAGACACGGAAGCCCGTTAAGCG

AAAGCTT

>CD171047

cttcaagttgactcatgagttggtatcttcgtggtttgatggacgaattttcagctgttaaaatgaaaagattctgatcaatggatggtatccaatgatactatgtgaccgaatgatggctactcaggatcgaattattaacgtaagtctctgtataacttgagataaaaggcaagacctagtttgataagccctggtgggttggtgtttgacgaaatctcgatatgaattcagaaaacactgttggctaatgtcaatttacgtcgcttgaggcgtaggtgagatatccctctaccaactaagggatgaattgactgcgcagtggttctgtacatcctactttaagtctgcgaaggatggccattatgagtagaggatatttgtaggttactagtattcggtcatagatgtcttcgtgtgctttagtacaat

>CONTIG8196

ccacatttgcatcagtaaaaagttcagaaggtcagtgaaagacgtgagaacggagagagaacccaatatgaattcagataaccgcccaatggtgaccaagatgaaactgaagctaaagaagcactgggaaactggagaaccagtattacttggtttggtacagccttccttcaagatactgacaaattcaaggtggctttcaaaaacaggttccaagccttacaagatatactcagagaagcggaaacgactgtggatgacaaccggaaaatgattaaagaagcagtaagttcaacatgccaggaggtcctggggcgcaagaagcaccaccatagagaatggatgtccgtcggtacactggacaagattcaagaaaggaaaaacaagatgacaaccgacaacagccgaacaagagcagagaacgtacaaggcaaatacacagaagcaaataggtatttaatttatttattttatttgaacacataaatatt

>CD093874

atacgacagactgcaaggcaaagttgtgcatggaggacagatgacggatgcgttccaagtgtggaacggtgtcaaacaagtctgcctattctattcctttttctttcttctggtagtcgacaggattatgaagacctccacatctaacaggaagcacggaatacaatggacaggttggatacaactagattatttggacttcacaagtgacctggcccttatatcccttacatacaatcatatgtaggtcaagacatcagtgtagcagacgcctctgcatcaggaggcctatgcatacacaaaggaaaaaccaagatcctcaaatacaacacggagggcaccgacctaataacacatgatggagaaactctggaagaggtagaaacttccacatacttggacagcatcatcgatgaagaaggaggatctaatgcgt

>CD166893

atatagctgtctcatatttcctccccttgcagcattttccactatcgttgccgggtttcccacatagttctgcttgtcgactataatgcttctcttcacttgcttgtttgcttctgcgtatt

>CD117558

taaacaaatattgcttatctctctaaccaattcttggtatgtctggatccccttattactctatagcaaagtgactagcagatctcctagaacccttacactatagtcctgagttcattgaatatgtaaaagaagtggataaaaatgatgaacgtattctctttcttgatgttgaatcagtatacaataatgttccacacacagagaacatattttttatccgcaagtacataaataatattgagatgaatatcggagtaccaaaagcttatatgaagggactactattacgatgcacttgtaatgcacaaactacattcaatgatatgattcatatgaaaattgatagcataacgataggatcccctcttggttcattactagcaaatgtctttatggccatatagaaaacaaccaacctaaaacgactatctcaaactcccacttgtatgaaaggtacataaatgatatttttatcatttgtaacaaaaatgtgacttgagtcatctaaagacgtttaataactatc

>CD202013

aaagaacatattacgccgggaaatagaggcagatatgaaaaggatgaatatcagctgaaaagaactggaaaggattgcccaggacagagttggatggagaatgcttgtgagcgccctatgctcctcgacgaagggtaacaggcgtaagtaagtaagtaatttgtttagaaataaaccacaaattatcatcccaatcaatgatactacagagttaatgaaattaaaaataagaaaaaaacattggaatttctttgttttacatgacacacattttcagattattccaatgatacttcaagagaaatatataataaaactgaattttatcttattaaaaagttacactattactcactgcattatataaagacatgaaactaagattatggaaactttaaagtctagtcaccctttaggataatctttatttataacaataaactagtctgattttgaattgcacaagcaagtggttatcaggactcagtggccgagtagataacgcgatggcgtttgaaacgaaaggtactgagttcgagtcccagagtgaaaatcaactctgagtcccaaataggacgaaacgcgcgtcanactggattccactgctagccactantcatctttgcttagtctgg

>CONTIG11905

ctaacacataggagatttttcttctcattgatgattatacctataaatgcatacatttactaacaatcttcaaagtacttatcaatggacaagataatatttgaaataatggtcacgtttgattctagagaaatgtagatcccaattaccctttatgtgaaatgtaggtcattatgatactgatttcgtgtattagaatgtgtaatccgcttgtttagaattgtcttcaggcctgatgctaaaatacaagaaacaattaagactgctaataaactacaccaaatacgtgagatgtttcttagttataatttaaatgaacacagtagaaaatcaaactggaatcaaccataggaaagttcatttaggaagtatctacggttgaaagaaagttaggggcgaccaaaccaaaacatagcatcagtgcttgaagtcactaacttctagtctgagccatgttggtagagcagactacctggttggggtccacgtgactatcgtaaccaatggtcgaagactctggatgacatggctcagaatcaatcacaacggcgtaggtgtatacactctttgtcttcccctaaacagtgagattaaaattactttatacctttatttctacgaactacttctttcttcctgtattatatcgttacacactttcttttatacattactaccattgaagtgactacttctgggaatttggtgttcatcttgttgtgctaatgaggtgtgtcaatgtgg

>CD094441

ctagggactgttaatttacgttgtcaggaaaacatctagcactaatggtcaggctctcagctcccaaaatgtttcggttgatataagcaaacattttagacagctaacaatgaacaaacttcttgtctcggataatttttagaaggcgtttaatgtatttcaacccgtaagataagagtagtttaacttctttatagactacttattctcactatgttcgagttcagagttagaagattattgttaactaatctcaacacaagtaagaaagcagtttctataatgatgtgcaaatccaggcttccttatgaaatcagtctgaataattattagaaggtgtattgtcacgtactttcaatgctacatagggtattccctatccaaatattcttctactgttagcaaatagcgataattctgttttaacattcatgtttaggtcttgtaacataatatattatcagattagacctttcacgaatatatagagaacaaacgtaatagcaatacaacggtttgctgcaacaacgcacttgtcagtaaatactgcccca

>CD163844

caaatcgcaccactacgtattatttttaaacagtcaattgaacgaaactcatcactatacatcaaccttcttgactatgagaaattatttgacaacgtgtgtaagagaaccttatagatcctctttcggcattatggtggactggaaaatgtggaaactttcacgtacctggtcagtattattgataaacaaaagggttctagagcagaaatgaagacaaggaatgacagagcgatgacagtactcttacaagtgagtgacttgtggaactcaagtcaactgtcagcaatata

>CF500921

cgatcgtgcacagaccaaatcgcgacagtacggatcatcgttgaacaatcaattgaatggatctcgtcactgtacatcaacttcgttgactgtgagaaggcgtttgatagtgtggtccggagcacattatggaaacttcttcgacgcttcgtgtacctgagaagatcctcaacatcatccagaattcatactacagtctacactccaaattggtgcacagaggacagctgatagacgcattctgagtgaggaccggtgtcagacaaggcttcttactttcaccacttctctttcttctggtgataggctgaattatgaaaaccttcatatctgatgggaagcgcagcgcacaatggacagcttaaatgcaactagacgatttggatttcatagatgacctagtccttctaccaatacacaccaacaa

>CD162597 (CL1090)

cttgtgcatggtttcgatagagatgtattccttatgatgcttcttgcaacccagcatcttctgatacgtcgaagttactgcttctttgatccctttccagtcgtcctccatcgtattttcctcttccttaaacagatcctgtaaggcttggaacctgttgttgagagttatcctgaattctttgagtttgtcagtatgttaaaggaaggctgtattgaacctttgttatgctgtttctccagttttccagtgctttttcagcttcaattttgtctttgcaaccaccagttggtagtggtgatctaaagctatgtcagctcctcttctggttctcacgtcttccattgatcttctggattttttagtgatgcaaatatgatcgatccttttagggatgtggaattccgaaacgtaacctatgaggatgaaaatcgatatacatttcaaaatgaactgaaagtggatacaaagtcaactntcaaaatgttcttcactacattaatttacggattttaatgcgttttcactgtttttactcagtgacaaattgtcattcataacttataatcattgtaatggatatacaactgattgatctaaatacacttctaattctacttagggaaactgcacttcctgctcaagtgggcagacnacgaatncagtgntatattaanaaatctgtatttaccaaaaaggggaatcnnttagatcgatggtacacccataantactggattttattagaaattcgaggat

>CONTIG3453

tggagaggcacaggaggagatgaaaagaatatctaatgtgtcgaaagaccaataatgatattaataacagtaataattataaatacgaacataagcagactttgtatattcagataaatataaaataaggtgaaatagacatgaaaagtttcaaaggtttcactttgcttatgatagtggttatgatagtgggatattgattggattaagcatgttttatgcatgatggtgttgctgcgcgctgattggttaattttaaaccgaccagcccgggcatattattgcagaaaaatctataagcttatgtatatactataaatatatgaacattatttaaactattgattgccgttcactgatcattattattttgaatacaatttcattcctgttcaacgtgttctgattttggggtcttgtcgagtgtcatcgtataagaatactaagcaataggaatagctgggtcgtttatatagcaaaaatacaattataacagtggcgacgaggaaaaaggaaaattcattcaaaatgcctatatcagatgaccagctcagccgaatactacaacaacagcaagcacaacttgatgcacaaatgcggttgttggagactctaacgcaacaacttaacatccaaaactcgagttcacacgctatttcaagtacttcttgtgatgcagttgctagcagcatcccagaagtcaactatgaccctaatagtggaaacactttagtcatgtggttcaagcgttgggaagatacatttagaacagaattctgtaaacaagataacgcgtggaaaactcaattgttgatgcataagttgggaatcaataaatatgcacgttatgctgaccatatattgccgaagttatctcgagagctcagtttcgaggacacgataactcaattgtcagagatattcggt

>CONTIG11198

ctcgcaaccagatgcacgttttgcccatgtgcatatcgacttggtaggtcctttaccacgttcaaacggttttaattatctttttacatgcatagatcgattaaccagatcccctatagccatcccgatagcggacatcacagcggaaacagtagccaaagttttcatgcagaactgggtaaccatttttggtacacctataacaataacgacggatagaggagcgcaattcgaatccgaaccatttaataacttggctaaacttctaggctccaataggatacgctggtttctttgctgctttgtgtgcttcggtgcgcgttgtggtgaggttactggactctgacccgggtcgatcgtgggcgggttcagctgttagtgtcgtgcagctagcaacgcgcgaggacatgttccacgttgcgtcaagcggctgtacttccatggt

>CD096910

gaaggtgagacttgtagatactgtttttttagtcacacctgtcttgttttctttaggtccctcatatttattaataaatttctgtgagtagcagttgtctcttagatatttttctgcgtttatcaatccttctagtttgttggcggtacatttcttttctactaaataaaagtgttttgatcaactctttctcgttactgattgggaaaaaactattgaaatttaggtaaagatcatccttagtcctttttcaataaaccgaacgtaaaagtgccttctcccctcctttgtatggcaatgtcaagaaagtggaacatgtcattattctcgtgttccatagttaaatagttctaacaggtggactgcacggtgtttactgttggagacaatatcggtgtcatcaacatatctggagtattccatcgttttgctaatcgcttgtttaaactctctgttttcaagataacccataaaaatttctgccataattggatctaatagacttcccgttgctacaccatcaatttgacggtatatagcgttattgaatcgaaattgaacgtattttgtgcacataagtagtacttttttgg

>CD068500

tggaagggttaggtcatctacgaagtccaaatcgtttagttacattcaaactgtccattttactccgtgcttctcctcaattgtggaggtcttcatgatccagtcaaccaccggaaataagaagaatggaggagagtaagcaaccttgtccgacacctatctttgttttgaacgtatctgtcagatgtcctgctttcacgactttgcagtgtaatctgtcgtatgaattccgtatgacgttgacgattttccctgacacagcatagtgtctaaacggttccattcacttcgccgatctacactgtccaacacttacttatagtcagggaagttgatgtatagtgatgacttttattcaattcgtttctgcacaatgatggtccgtattgcacagtataatcagatgtgattacaatgtaattttacttacttacttacgcctgtt

>CD195288

ttcggtctactcaaaggactagaagaccgtatgtttaaacctgattttctaggcgactgtaaacgatattttttttaacttttccttcgttaatttcgatagaactgaaatgaaagtttagttatcaggatctaggttttgtgatggatgtaaacatgtgaaacacttgaagactgatgttcactttgagaatccatttacgcagacacaagacttggtaccctcgacaaaggtcgaataagaaagagttaagactaaactggcttactgctataaccaatgtagaagtaacaagcaacacacaaaggctatattgactgataaacatcgagaggccatctggaaactaaaatcgaaggaacacacatttgggctcaagccttctggaacacgcacccttcgactctatggcctacttaacgttcagaaaccgggttttcttcacgtctaattctgaacatgtcaaactcaccataccattcaaaagcgaaatggttaatggaattattagaaccattacaccaagactntattaagtacagcattaaaaacgtatttaaatttctgaaatacaaaaaaacaaaaaaacggcgctatcactagacataatatctntggtc

>CD066558

agaacacgtggtggaagtggataggatgcacattgatgaaatcacacaattgcatcacaagacaacccctcacatggaatcctgaaggtcaaaggacaagaggaagacctaagaacacattacgccgagaaatagagacagacatgtaaagaatgaacaacaactggataaaactagaaagaaaggcctagaacaaaatgggttatataatgctggtcggtggcctatgttccactgagagtaacagacgttagtcactaaaaaacctattaacagcttacaaaaaagaatatcaaattcaggagattctacctcatatatagaatgtttcgtcttactgtacacctccctcagatggtcttatctataaccaacctccctaaccactgataatacttggctcaatcctaatatattttttaagtattttaaatatttacctcactctatcaatcttaatgaatatataccccactattcatgtacaataatgcccccaaataccctggtaccaaccggaggtaggagaaaccgctctccctc

>CD186839

gacaactggagaaacagcgctacaaaggtctaatacatccttcctttgagatactaacaaactcaacgaacttacgatagctcttagcaacaggttccaagctttacaagatctactcaaagagaaagaaactatggaggacaactggaaaagggttaaagaaacaatagcctcaatgtgttaggaggatcagggcctcaggaagcaccattataaggaatggacctccatcgaaaccctgagtagcattcaggaaaagaataaagagaagatgacaatcagcatcagccgcacaagaacagagaaagtcaaggcacaagctgaataaacagaagcaaacaagcaagtgaagaacattagggccgacaagcagaaaaagctgaaagagaaggaaatatgagacagctatatgacacaacaaagagactagcaaggaaatatattaaaccagagcgattaagtca

>CF499420

catctgcattacttacccggtggtcccaggatatacgagcaatgcgtcgaaaacacctatgatcgaatactagtagcctacgaatatcctctactcttatcggccatgtttcactaccataaagtaggacggaacaaactgcagcacagtaaactcgttctttaggttgctagacggatatctcgcctacgccataaatggcgcaagttggcgaaagctagacgagccttctgtatccgtgctgagatttcgtcacacactagaccacaagggctgatgagacttccaagataagtgaagctgtcaacactctcaactacttcactccctatcattagttcaggtgtcgatgcaacccaatcctggagtaacattctgcacttcgagggagagaatcgcatcccgaacatgcctgcattgctgcttatagtggtcagaagactctgcattttgtcagcgtcttcaccaaataaaactatgtcgtcggcgtattctaagtcagcctccaaatgccctggtacagccgagagtggggagagtctgctccccctctcgaaatgctctca

>Contig5706 EST cluster from Schistosoma mansoni 28/06/2002, 292bp

ttaatatggtattgtgatagacggaggatcacgaaagttaatagacggaa

acactaagttatccgtttgcgtaattcctttttctggttgcaggttatcc

ccagtcacaatcaagcacaccatagaccccctatatcaaccattactgga

caaatactccaggatataccaaccgcagccaaaattgccttgtgtaacca

gtaatgttgcacatcacatctcgactacaggaccacctgtattctcgaaa

gcccgccgactcgctcccgaaaagcttaggttggctaaaaat

>CD091443

gtcccatggattcaatgcagttggtccattcaatagttactcaaaatcttctactcatctgttcctctgtcatcgactctcactgattgtcttgccttctatgtccttgatcggagtctctagttagccatatttccgttccactttcttcgttgtatcatatagttgtttcatatttacttctcttgtagctttttccactgtcacttctacgtcttccacgtgtttcagtatatttcagatctaatgctcctgaccatttgctagtttgcttcagtgtattcaacatgtgccttaactttccatgctcgcgttcggttgtagataattactgtcttcttgttcttgcgtacatcaatattgtctggggttctaacagagatccattcctcataatgatgcttgttgcggcccataaccccctgacacatagaagttagtgcttctatcatccgtttccagttgg
