# Supplementary figures and images for: Use of Genomic DNA as an Indirect Reference for Identifying Gender-Associated Transcripts in Morphologically Identical, but Chromosomally Distinct, Schistosoma mansoni Cercariae
Source: PLoS Negl Trop Dis. 2008 Oct 22;2(10):e323. doi: 10.1371/journal.pntd.0000323 (PMC2565838; doi:10.1371/journal.pntd.0000323)

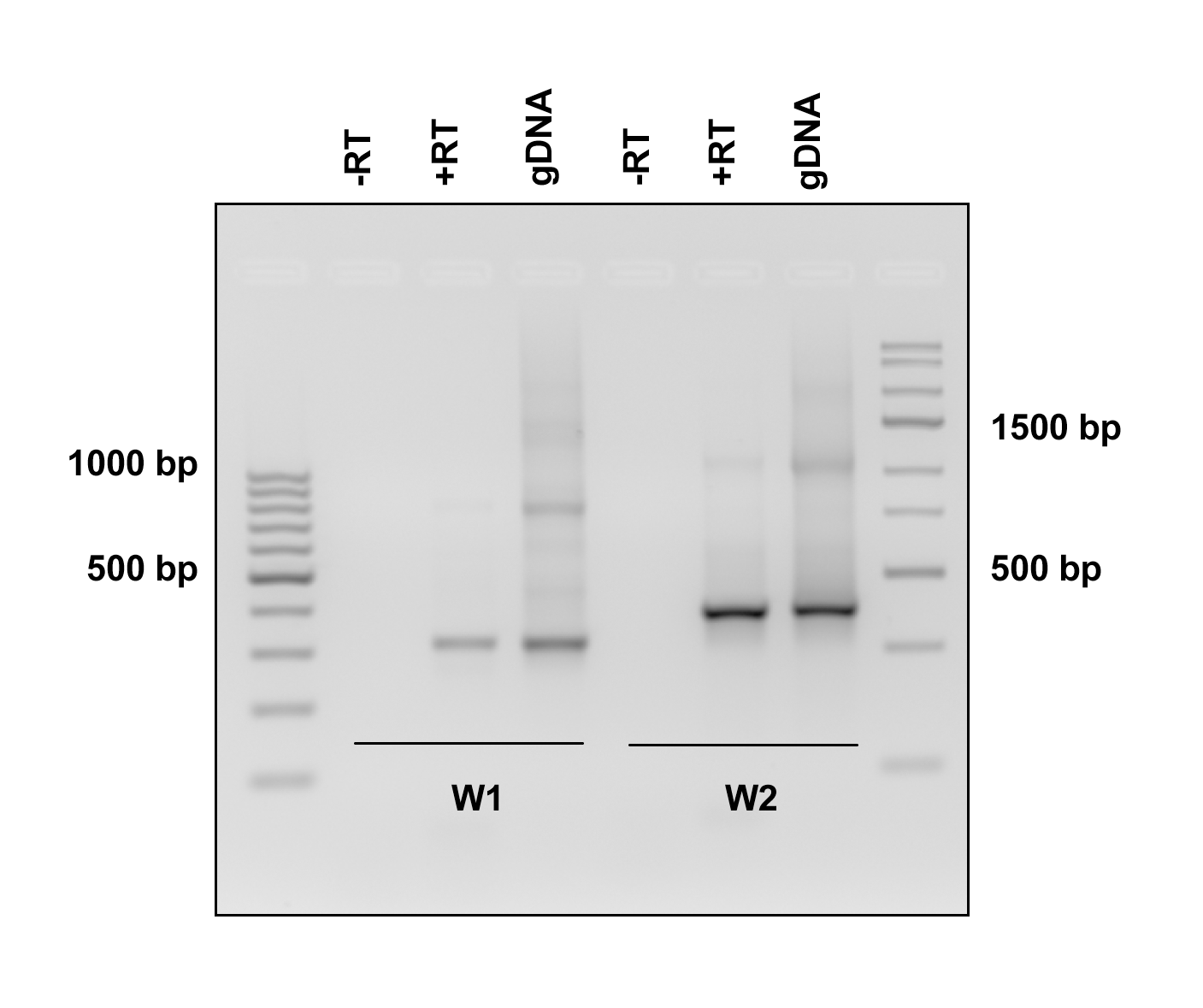

Supplement: Figure S1 — RT-PCR confirmation of W1/W2 from mixed-sex cercariae cDNA. Mixed-sex cercaria RNA was used in a reverse transcription reaction as described previously [20]. -RT indicates PCR performed from cDNA samples prepared in the absence of reverse transcriptase, +RT indicates PCR performed from cDNA samples prepared in the presence of reverse transcriptase, gDNA indicates PCR performed from mixed-sex cercariae gDNA. PCR utilized the following cycling parameters: 95°C for 3 min, 94°C for 1 min, 59°C for 1 min, 72°C for 1 min. Steps 2–4 were repeated 39 times (total of 40 cycles). PCR amplicons were electrophoresed on a 2.0% agarose gel. W1 and W2 amplicons were subcloned into TOPO 4.0 (Invitrogen). Representative clones were sequenced at the IBERS gene sequencing service unit (ABI3130xl sequencer and BigDye chemistry). W1 and W2 expressed sequences were deposited in GenBank (W1, still awaiting and W2, EU980106). (7.00 MB TIF) [file pntd.0000323.s006.tif]
